# Presynaptic accumulation of APP-CTFβ may contribute to synaptic dysfunction in Alzheimer’s disease

**DOI:** 10.1038/s44318-026-00899-6

**Published:** 2026-09-01

**Authors:** Akshay Kapadia, Fabian Schuhmann, Ezgi Daskin, Jochen Walter, Isabell Lindahl, Neda Rahmani, Tjado H J Morrema, Annemieke J M Rozemuller, Baayla D C Boon, Weria Pezeshkian, Anne-Sophie Hafner

**Affiliations:** 1 Donders Institute for Brain, Cognition and Behavior, Radboud University, Nijmegen, The Netherlands; 2 Niels Bohr International Academy, Niels Bohr Institute, University of Copenhagen, Copenhagen, Denmark; 3 Department of Neurology, University of Bonn, Bonn, Germany; 4 Department of Pathology, Amsterdam UMC location VUmc, Amsterdam, The Netherlands; 5 Department of Neuroscience, Mayo Clinic Jacksonville, Jacksonville, FL USA; 6 Alzheimer Center Amsterdam, Neurology, Vrije Universiteit Amsterdam, Amsterdam UMC location VUmc, Amsterdam, The Netherlands; 7 Interdisciplinary Institute for Neuroscience (IINS), Université de Bordeaux, UMR 5297 CNRS, Bordeaux, France

**Keywords:** Molecular Biology of Disease, Neuroscience

## Abstract

The study of Alzheimer’s disease (AD)-associated mutations has implicated dysregulation of amyloid precursor protein (APP) proteolysis in the disease. Brain recordings have revealed synaptic hyperexcitation during asymptomatic and early stages of AD, reverting to overinhibition as dementia progresses. Here, we show that endogenous APP and its proteolytic C-terminal fragments (APP-CTFs), the precursors of amyloid-β (Aβ), are enriched at excitatory synapses. Pharmacological modulation of endogenous APP metabolite levels suggests a role for APP-CTFs, in particular APP-CTFβ, in regulating glutamatergic synaptic transmission. Presynaptic accumulation of APP-CTFβ promotes its oligomerization, increases synaptic vesicle docking, and causes vesicle release defects, accompanied by enhanced neuronal network activity. Examination of post-mortem AD patient brains yields consistent results, namely, elevated APP-CTFβ levels at synaptic compartments and enlarged excitatory presynaptic boutons. Strikingly, acute application of Aβ preparations enriched in monomeric species counteracts APP-CTFβ-induced hyperexcitability. Our findings indicate a role for presynaptic APP-CTFβ in modulating excitatory synaptic function and network activity, suggesting that amyloidogenic APP processing intermediates may contribute to early synaptic alterations in Alzheimer’s disease.

## Synopsis

Dysregulation of amyloid precursor protein (APP) proteolysis is involved in Alzheimer’s disease. This study shows that APP processing generates functionally distinct intermediates at the synapse, i.e. presynaptic APP-CTFβ, accumulation of which causes neuronal network hyperactivity, and extracellular amyloid-β (Aβ), which can counteract APP-CTFβ-induced network hyperactivity.

- Endogenous amyloidogenic processing of APP occurs at excitatory presynaptic active zones.
- Presynaptic APP-CTFβ accumulation promotes synaptic vesicle recruitment, neurotransmitter release, and neuronal network hyperactivity, independent of Aβ.
- Acute Aβ treatment attenuates APP-CTFβ-induced hyperactivity.
- APP-CTFβ oligomerization regulates PIP2 interactions and exposure of its C-terminal domain, providing a mechanism for enhanced vesicle recruitment.
- Alzheimer’s disease patient brains exhibit elevated synaptic APP-CTFβ and enlarged excitatory presynaptic boutons.

## Abstract (web summary)

APP-CTFβ, the precursor of amyloid-β, can accumulate in the active zone of excitatory synapses and promote neuronal network excitation/inhibition imbalance.

## Graphical abstract

**Figure:**
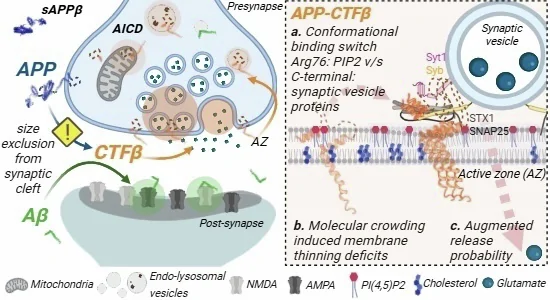

## Introduction

Decades of research have centered on amyloid-β (Aβ) as a central component of Alzheimer’s disease (AD) pathophysiology, owing to its aggregation propensity and extracellular deposition as plaques, supported by extensive genetic, biochemical, and clinical evidence (Walsh and Selkoe, 2007; Hampel et al, 2021). Genetic mutations in Amyloid-beta precursor protein (APP) and presenilin (PSEN, catalytic component of γ-secretases) genes that alter Aβ production or its relative abundance have been implicated in disease initiation, wherein soluble Aβ species have been shown to correlate with synaptic dysfunction (Cerpa et al, 2008; McGeachan et al, 2025; Wei et al, 2010; Takahashi et al, 2004; Rowan et al, 2004; Sehar et al, 2022; Wilson et al, 2025; Martinsson et al, 2022). APP, a transmembrane protein highly expressed in neurons, is critical for neuronal and synaptic function (Chen et al, 2024); it undergoes complex proteolysis. Amyloidogenic and non-amyloidogenic processing pathways generate multiple fragments beyond Aβ and P3 peptides, including extracellular soluble APP species (sAPPβ/α), membrane-bound APP C-terminal fragments (APP-CTFβ/α), and cytosolic APP intracellular domain (AICD) (Appendix Fig. S16; Ling et al, 2003; Haass et al, 2012), whose contributions to synaptic physiology and neurodegeneration remain relatively underexplored (Müller et al, 2008; Nhan et al, 2015; Tyan et al, 2012). Nevertheless, emerging evidence suggests that APP-CTFs, in particular APP-CTFβ, appear capable of triggering neuronal dyshomeostasis independent of Aβ (Tian et al, 2014; Sergeant et al, 2002; Lauritzen et al, 2019b; Lee et al, 2020; Luo et al, 2025; Bretou et al, 2024; Tamayev et al, 2009, 2012; Vaillant-Beuchot et al, 2021; Bécot, 2019; Lauritzen et al, 2019a; Lee et al, 2022). Understanding the precise and complementary roles of these fragments in physiological and pathological contexts remains an important challenge.

AD transgenic animal models, which often incorporate multiple familial AD mutations, have been instrumental in advancing our understanding of Aβ biology and plaque pathology. However, these models frequently involve APP overexpression, altered processing dynamics, influencing the relative abundance, localization, and interactions of APP-fragments and metabolites (Kokjohn and Roher, 2009; Tai et al, 2021; Mckean et al, 2021; Granzotto et al, 2024). Similarly, while the use of synthetic APP-derived peptides has provided valuable insights, they may not fully recapitulate endogenous concentrations, biological conformations or (sub)cellular localization or distributions. These methodological and technical limitations can complicate the interpretation of functional studies (Randall et al, 2010; Reinhard et al, 2005), particularly when aiming to disentangle the roles of individual APP processing products under physiological concentrations. As a result, despite substantial progress, there is a persistent knowledge gap regarding the physiological and pathological roles of APP proteolytic products beyond Aβ, despite their important contributions to neuron (patho)physiology.

Therapeutic strategies targeting Aβ production, aggregation, or its clearance have been extensively pursued in AD and faced significant challenges in clinical trials. Aβ-directed antibody therapies have recently demonstrated measurable reductions in amyloid burden, but for now, only very modest clinical benefits. This highlights the complexity of translating these effects to a functional outcome. Moreover, APP and its cleavage products have been shown to play roles in synaptic function (Müller et al, 2008; Nhan et al, 2015; Tyan et al, 2012), and targeting Aβ or modulating its levels possibly influences broader APP-dependent processes that are essential for synaptic/neuronal homeostasis. Addressing these concerns requires a more comprehensive understanding of APP processing and its diverse functional consequences, particularly at the synapse, in order to better delineate the multifaceted AD pathology.

In this study, we investigate the differential localization, site of action, and function of endogenous APP and its proteolytic fragments in synapse subtypes. Using a combination of fluorescence-activated synapse sorting, biochemical fractionations, high-resolution imaging, functional assays with in vivo and in vitro models as well as in silico computational modeling of the human APP-CTFβ (C99), we examine how APP processing products influence synaptic activity and neuronal network dynamics. Finally, we validate the presence of APP-CTFs/β species and their abnormal abundance in excitatory synapses in postmortem AD brain samples. Our findings reveal distinct presynaptic roles for APP-CTFs/β, in modulating synaptic vesicle dynamics and network activity, suggesting Aβ-independent mechanisms in shaping early synaptic alterations in AD.

## Results

### APP and enzymes of its amyloidogenic proteolysis predominantly localize in excitatory presynapses

Using biochemistry and immunofluorescence assays, APP and its processing enzymes have been shown to be present and locally synthesized at synapses (Restituito et al, 2011; Schilling et al, 2023; Groemer et al, 2011; Cefaliello et al, 2020). Nonetheless, efforts to localize those proteins in different synaptic compartments of animal brains have been challenged by the small size (a few micrometers) and density/proximity of these compartments in tissue (Appendix Fig. S1). In addition, because APP and its proteolytic products share substantial peptide-sequence homology, making it challenging to use proteomic approaches to infer the relative abundance of APP versus APP-CTFs versus Aβ.

**Figure 1:**
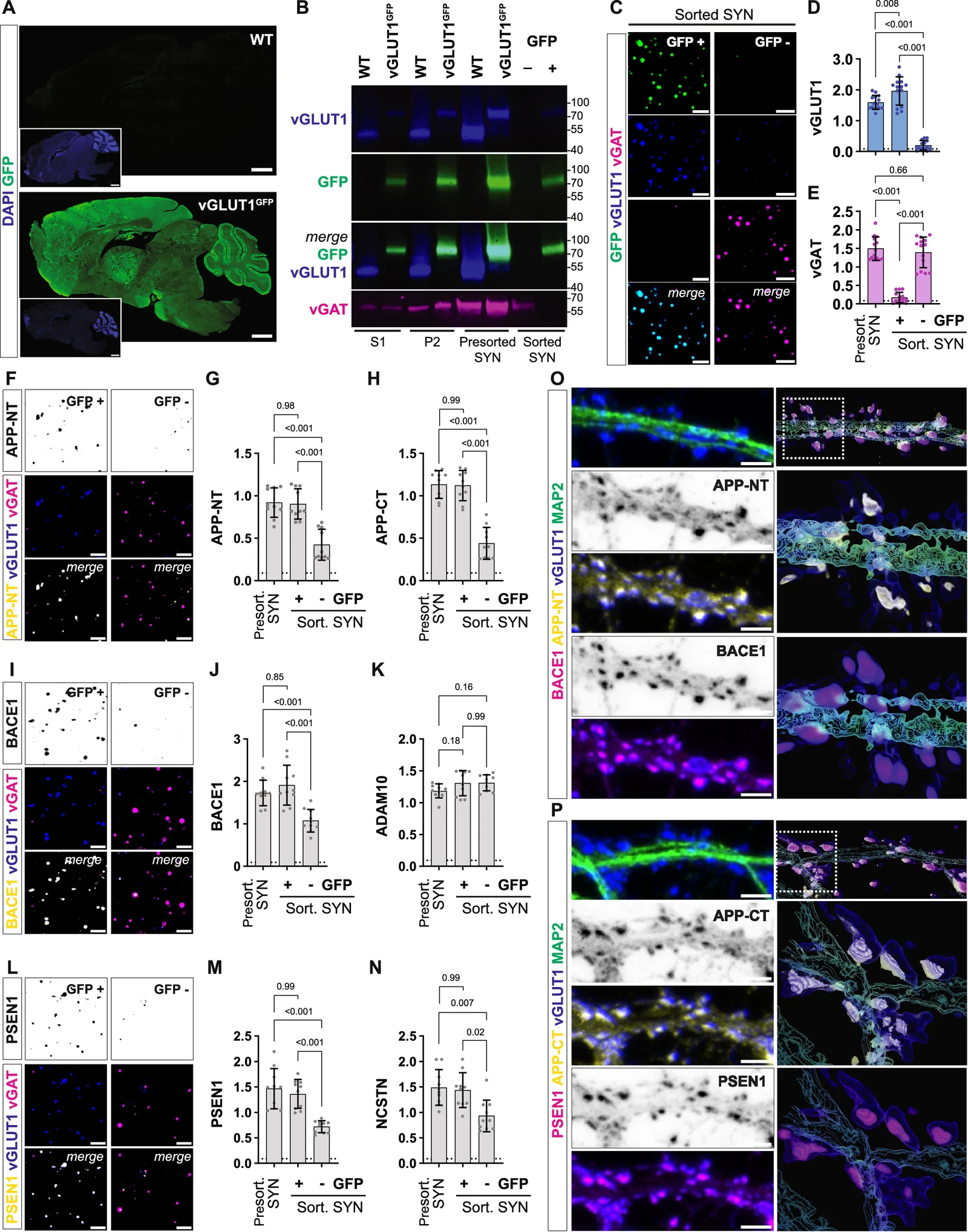
Amyloidogenic processing of APP predominantly occurs at the excitatory synapse. (**A**) Immunohistochemical analyses of wild-type and vGLUT1^GFP^ transgenic mice sections (male, 6 weeks) showing GFP-positive vGLUT1 (vesicular glutamatergic transporter 1, green channel) signals. Sections were co-stained for DAPI to visualize the nucleus (blue channel). Scale bar = 100 μm, *N* = 2. (**B**) Western immunoblots depicting the differential stages of synaptosome enrichment from WT and vGLUT1^GFP^ mice forebrain (male, 1 year); and sorting (Fig. EV1A, *scheme*) of GFP+ (GFP-positive) and GFP- (GFP-negative) synapses. Blots were probed for α-vGLUT1 (blue), α-GFP (green), and α-vGAT (vesicular GABAergic transporter, magenta). Protein load- S1, P2, presort. SYN = 20 μg, Sort. SYN = 1 μg. *N* = 5. (**C**) Immunocytochemical (ICC) staining of sorted synapses co-stained for vGLUT1 (blue) and vGAT (magenta). Scale bar = 1 μm, *n* = 10, *N* = 5. (**D**, **E**) Indirect ELISA analyses depicting enrichment of vGLUT1 in GFP+ synapses (**D**) and vGAT in GFP− synapses (**E**), respectively. Bar plots represent mean ± SD; *n* = 15, *N* = 5. (**F**, **I**, **L**) ICC of sorted synapses stained for APP-NT (beta-amyloid precursor full-length protein, clone: M3.2; **F**, gray, yellow-merge), BACE1 (beta-amyloid precursor protein cleaving enzyme 1; **I**, gray, yellow-merge), and PSEN1 (presenilin-1, catalytic component of γ-secretase complex, **L**, gray*,* yellow-merge) along with vGLUT1 (blue) and vGAT (magenta). Scale bar =  1 μm, *n* = 10, *N* = 5. (**G**, **H**, **J**, **K**, **M**, **N**) Indirect ELISA analyses of presorted synapses (*P3 fraction*), and sorted GFP+ and GFP− synapses quantifying relative levels of APP-FL and APP-CTFβ (**G**), APP C-terminal fragments (APP-CT, clone: 140-rb, **H**), BACE1 (**J**), ADAM10 (metalloproteinase-10, α-secretase; **K**), PSEN1 (**M**), and NCSTN (Nicastrin, component of γ-secretase complex, **N**) in presorted and sorted GFP+ and GFP− synapse fractions. Bar plots represent mean ± SD; *n* = 10, *N* = 5. (**O**, **P**) Immunocytochemistry and Imaris 3D reconstruction of rat primary cortical neurons (DIV21) depicting localization of APP-NT (clone: M3.2, **O**, grayscale, yellow-merge); BACE1 (**O**, magenta) and APP C-terminal species (clone: C1/6.1, **P**, grayscale, yellow-merge) and PSEN1 (**P**, magenta) in excitatory synapses (vGLUT1 + , blue). Neurons were co-stained with MAP2 to visualize the dendrites (green). Zoomed-in sections have been indicated with a white box. Scale bar = 5 μm, *N* = 3. ns (*P* > 0.05), *(*P* ≤ 0.05), **(*P* ≤ 0.01), ***(*P* ≤ 0.001); one-way ANOVA. Source data are available online for this figure.

**Figure EV1:**
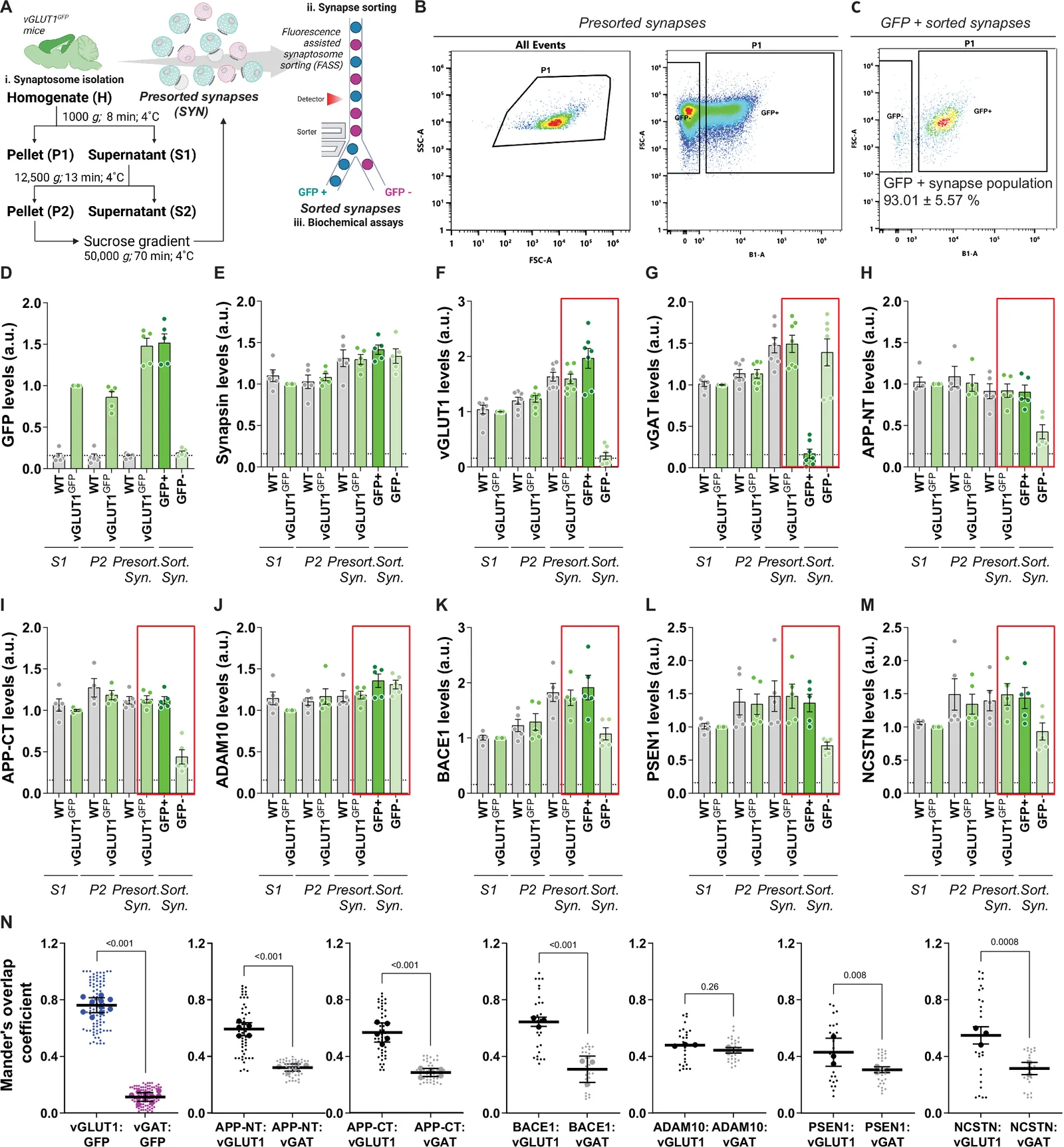
FASS and biochemical analyses of GFP+ and GFP− synapses from vGLUT1^GFP^ mice. (**A**) Schematic representation depicting synaptosome isolation (i) and FAS sorting (ii) of GFP+ (GFP-positive) and GFP− (GFP-negative) synapses from vGLUT1^GFP^ mice forebrain (m, 1 y) for further biochemical analysis (iii) using ICC and ELISA. (**B**, **C**) Sorting of synapses (singlets, all events, and gating of GFP+ and GFP– synapse populations). Sorted GFP+ synapses were rerun to determine the purity of the sorted population (**C**). Values indicate the mean and standard deviation of 5 experimental replicates, *N* = 5 animals/experiments. (**D**–**M**) Indirect ELISA analyses depicting levels of GFP (**D**), synapsin (**E**), vGLUT1 (**F**), vGAT (**G**), APP-FL (**H**), APP-CT species (**I**), ADAM10 (**J**), BACE1 (**K**), PSEN1 (**L**), and NCSTN (**M**) in all fractions starting from S1, P2, unsorted synapses and sorted synapses. Values were normalized to the S1 fraction of vGLUT1^GFP^ mice. The dotted line indicates average values from blank wells. As seen in the graphs, GFP signals are specific to the transgenic mice and the sorted population of the synapses. The red box highlights data depicted in Fig. 1D,E,G,H,J,K,M,N, respectively. Bar plots represent mean ± SEM; *n* = 10–15, *N* = 5. (**N**) Mander’s overlap coefficient of overlap determined from ICC analysis of sorted synapses. vGLUT1/vGAT: GFP *n* = 20, *N* = 5; APP-FL/APP-CT: vGLUT1/vGAT, *n* = 10, *N* = 5; ADAM10/BACE1/PSEN1/NCSTN1: vGLUT1/vGAT. Scatter plots represent individual values; smaller dots, per image; larger dots, experimental replicates; bold line, median ± SEM. *n* = 10, *N* = 5. ns (*P* > 0.05), *(*P* ≤ 0.05), **(*P* ≤ 0.01), ***(*P* ≤ 0.001); one-way ANOVA. Source data are available online for this figure.

To circumvent these challenges, we optimized the method of fluorescence-activated synapse sorting (FASS) (Hafner et al, 2019) to obtain two pools of synapses, respectively enriched in excitatory (GFP-positive, containing vesicular glutamate transporter 1 (vGLUT1)) and excitatory-deenriched synapses (GFP-negative, containing vesicular GABA transporter (vGAT)) from 1-year-old mice and investigated their content using western blotting and ELISA assays (Figs. 1A–E and EV1A–G). The “presorted” and “sorted” synaptosomes constitute resealed presynaptic compartments, sometimes associated with “open” postsynaptic membranes (Appendix Table S3 and (Hafner et al, 2019)). We validated the enrichment of both pools using immunocytochemistry on synaptosomes plated at low density on coverslips (Figs. 1C and EV1N) and ELISA assays (Figs. 1D,E and EV1D–G).

Using these approaches, we then investigated the relative enrichment of endogenous levels of membrane-bound APP, APP-CTFs - precursors of Aβ, as well as enzymes involved in APP proteolysis in presorted and sorted synapses (Figs. 1F–N and EV1H–N). We found that full-length APP-FL (detected using APP-NT, AB#3), APP C-terminal species (detected using APP-CT antibody clone: 140-rb), and enzymes of the amyloidogenic pathway β-secretase (BACE1) and *γ*-secretase components (PSEN1 and NCSTN) are present in excitatory synapses. While some signals were detectable for vGAT-enriched (GFP-negative) synapses, immunocytochemistry revealed that most vGAT-positive synapses did not contain those proteins as opposed to vGLUT1-positive synapses (Figs. 1F,I,L and EV1N). Interestingly, the metalloproteinase ADAM10, which cleaves APP *via* the non-amyloidogenic pathway is equally abundant in all synapse types (Figs. 1K and EV1J). We further confirmed the presence of APP-FL, APP-CT species, β-secretase, and *γ*-secretase in synapses of dissociated rat neurons (Appendix Fig. S1E–I) by performing immunocytochemistry (Fig. 1O,P; Appendix Fig. S1C,D). Our results confirmed their accumulation in excitatory synapses detected using an antibody against vGLUT1.

### Accumulation of APP-CTFβ is associated with network hyperexcitability

**Figure 2:**
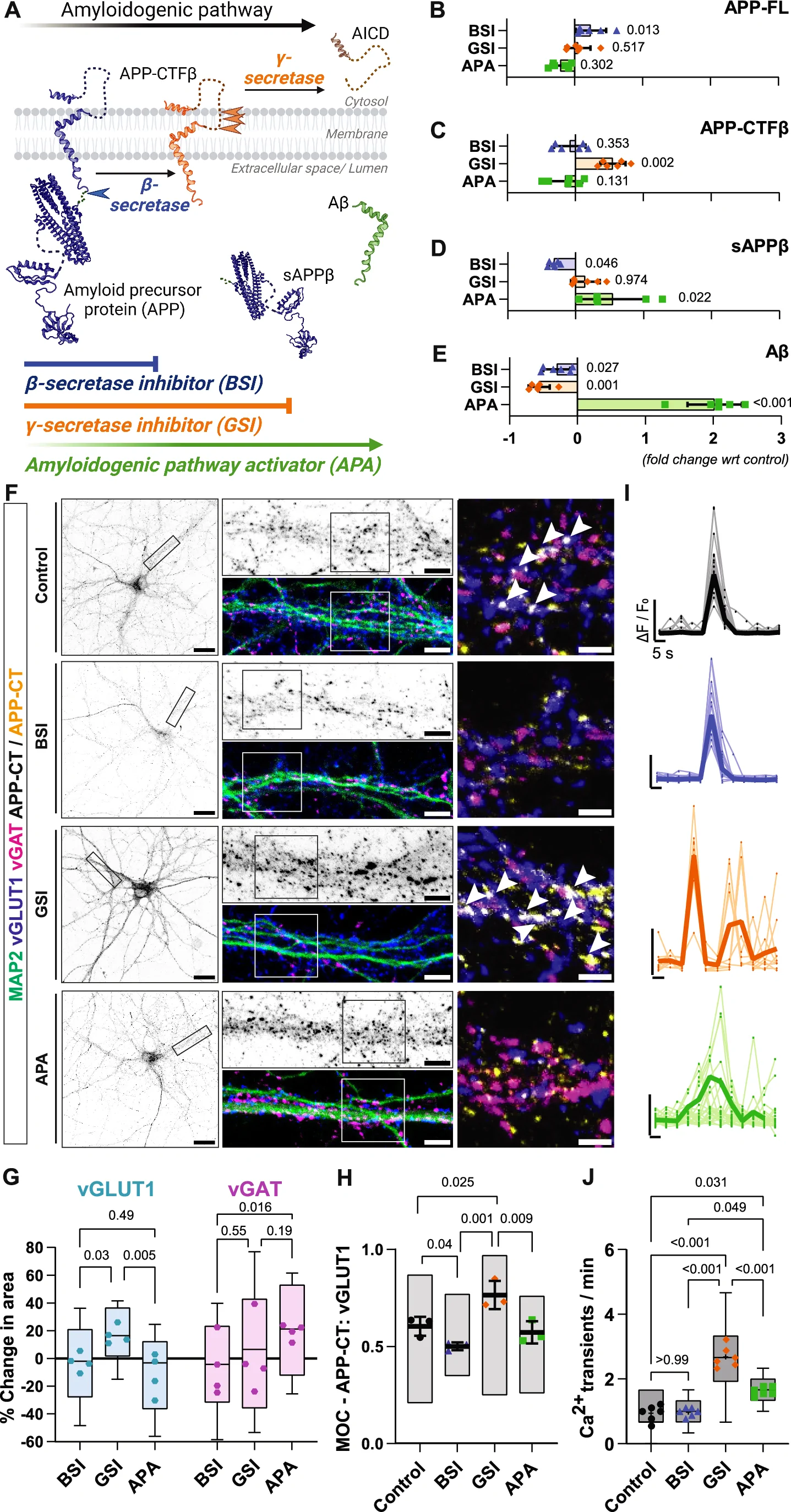
Pharmacological manipulation of amyloidogenic proteolysis has a differential impact on synaptic activity. (**A**) Schematic representation of APP proteolysis *via* the amyloidogenic pathway. Initial cleavage by BACE1 results in the secretion of soluble APP species (sAPPβ), leaving a membrane-bound C-terminal fragment (APP-CTFβ). γ-secretase cleavage and sequential trimming of APP-CTFβ additionally release Aβ peptides in the extracellular space or lumen and intracellular AICD (amyloid intracellular C-terminal domain), respectively. Rat cortical neurons (DIV21) were treated with LY2886721 (1 μM; BACE1 inhibition, BSI), DAPT (10 μM; γ-secretase inhibition, GSI), or Aftin-4 (5 μM; activator of APP processing, APA) for 4 h, to pharmacologically manipulate various stages of APP proteolysis. (**B**–**E**) Quantitative analyses from western immunoblots (**B**, **C**), ELISA (**D**, **E**), and depicting fold change in the levels of membrane-bound APP-FL (**B**), APP-CTFβ (**C**), and secreted sAPPβ (**D**), Aβ (**E**) detected in neuronal homogenate and culture media, respectively. Bar plots represent mean ± SD; *n* = 6, *N* = 3. (**F**) ICC depicting relative changes in the levels of APP C-terminal species upon treatment with different compounds (clone: C1/6.1, gray, yellow-merge). Neurons were also co-stained with vGLUT1 (excitatory synapses, blue), vGAT (inhibitory synapses, magenta), and MAP2 (dendrites, green). Box selection depicts the zoomed-in panel. Arrows depict colocalization of APP-CT species with vGLUT1+ synapse. Scale bar = 1 μm, *N* = 4. (**G**) Percentage change in vGLUT1 and vGAT-stained presynaptic bouton area following pharmacological modulation of APP processing was quantified and presented as a box and whiskers plot; center line, median; box boundaries, 5th and 95th percentiles; whiskers extend to the minimum and maximum values; *N* = 4. (**H**) Scatter plots depict Mander’s overlap coefficient of colocalization between APP-CT signals and vGLUT1-positive boutons. Box extends to the minimum and maximum values; center line, mean; *N* = 3. (**I**, **J**) Neurons expressing Camk2-GCaMP6 (AAV transduction, DIV3) were subjected to treatment with different modulators (DIV21, indicated above). Spontaneous calcium transients were recorded in the absence and presence of Ca^2+^ in Tyrode buffer. Ca^2+^ traces per minute have been depicted, respectively, as (individual traces—thin lines; average—thick lines; **I**). Box and whiskers plot (**J**) depicts the average number of spontaneously evoked somatic or dendritic Ca^2+^ transients per minute, following pharmacological modulation of APP proteolysis. Each data point indicates an experimental replicate; center line, median; box boundaries, 5th and 95th percentiles; whiskers extend to the minimum and maximum values; *n* = 6; *N* = 3. ns (*P* > 0.05), *(*P* ≤ 0.05), **(*P* ≤ 0.01), ***(*P* ≤ 0.001); one-way ANOVA. Movie files have been provided in SI. Source data are available online for this figure.

**Figure EV2:**
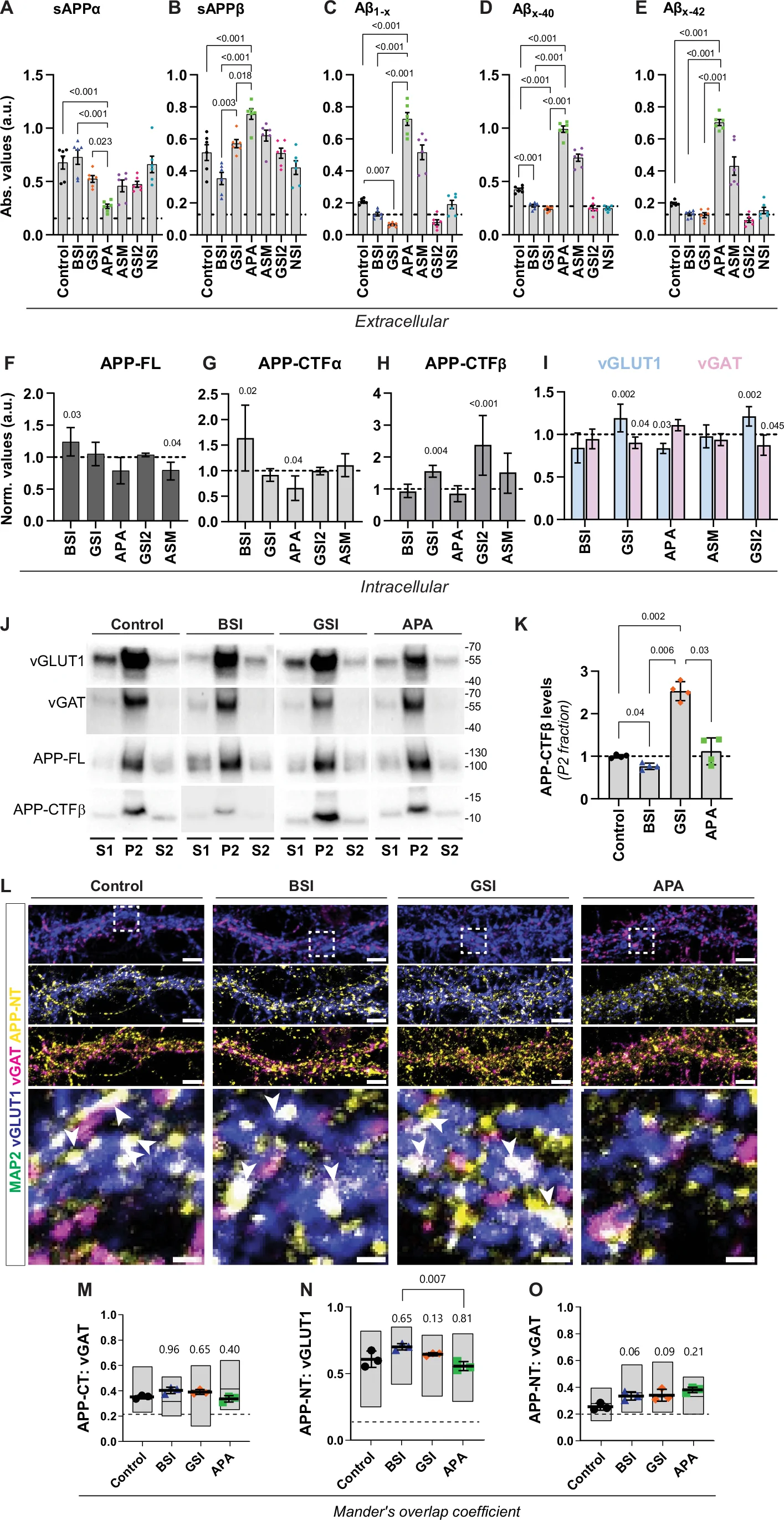
Pharmacological modulation of APP processing alters the distribution of synaptic proteins in cultured rat neurons. (**A**–**I**) Rat cortical neurons (DIV21) were treated with LY2886721 (1 μM; BACE1 inhibition, BSI), DAPT (10 μM; γ-secretase inhibition, GSI), or Aftin-4 (5 μM; activator of APP processing, APA) for 4 h, to pharmacologically manipulate different stages of APP proteolysis. In addition, we tested GI25423X (1 μM; α-secretase modulator, ASM), Compound E (10 nM; γ-secretase inhibition, GSI2), and FLI-06 (10 μM; inhibitor of the notch signaling pathway, NSI) for 4 h. Post-treatment media were used to detect levels of sAPPα (**A**), sAPPβ (**B**), Aβ (**C**), Aβ_x-40_ (**D**), and Aβ_x-42_ (**E**) using indirect ELISA. Bar plots represent mean ± SEM. Neuron homogenate was subjected to western blotting to examine changes in the level of APP-FL (**F**), APP-CTFα (**G**), and APP-CTFβ (**H**) along with vGLUT1 and vGAT (**I**), respectively. Bar plots represent mean ± SEM; *n* = 6, *N* = 3. (**J**, **K**) Neurons post-treatment was subjected to synaptosome enrichment, loaded on western blots to examine changes in levels of proteins between the fractions. While APP-FL remains relatively comparable in the P2 fraction, differences in levels of APP-CTFβ are significant. The dotted line represents the average levels of APP-CTFβ within P2 (synaptic) fraction in untreated conditions. Bar plots represent mean ± SD; *n* = 4, *N* = 2. (**L**) ICC depicting relative changes in the levels of full-length APP (APP-FL) upon treatment with different compounds (gray, yellow-merge). Neurons were also co-stained with vGLUT1 (excitatory synapses, blue), vGAT (inhibitory synapses, magenta), and MAP2 (dendrites, green). Scale bar = 5 μm. Box selection depicts the zoomed-in panel. Arrows depict colocalization/overlap of APP-FL with vGLUT1+ synapse. Scale bar = 1 μm, *N* = 3. (**M**–**O**) Box plots depicts Mander’s overlap coefficient (MOC) of colocalization between APP-FL: vGLUT1 (**N**). MOC values between APP-CT (**M**, images depicted in Fig. 2F) and APP-FL (**O**) with vGAT-positive boutons show no significant changes among treatment groups. Box extends to the minimum and maximum values; center line, mean; *N* = 3. ns (*P* > 0.05), *(*P* ≤ 0.05), **(*P* ≤ 0.01), ***(*P* ≤ 0.001); one-way ANOVA. Source data are available online for this figure.

We found the amyloidogenic proteolysis of APP to be occurring at excitatory synapses; thus, we investigated the effects of manipulating the relative abundance of those products using pharmacology to systematically disentangle the role of each component. We note that our manipulations rely on endogenous levels of rodent APP, without overexpression, allowing functional dissection of presynaptic effects under a plausible physiological range. Our treatments consisted of a 4 h incubation with one of the following drugs applied directly in the cell culture media: (i) LY2886721 to inhibit β-secretase (BSI), (ii) DAPT to inhibit *γ*-secretase (GSI), and (iii) Aftin-4 to promote the entire pathway in order to increase the production of Aβ (APA) (Figs. 2A–E and EV2A–H). BSI led to a predicted decrease in soluble extracellular products, Aβ, and sAPPβ; and a small, yet significant accumulation of intracellular APP-FL. Modest changes in APP might result from its endogenous high expression in neurons and/or compensatory mechanisms. In our experiments, β-secretase inhibition decreased synaptic but not global APP-CTFβ levels (Figs. 2C and EV2G,H,J,K). GSI led to ~50% reduction of extracellular Aβ levels (detected in media) and an accumulation of synaptic APP-CTFβ (~ 2.5-fold increase, EV2J–K) in excitatory boutons (Fig. 2F,H). Finally, APA led to an increase in sAPPβ and Aβ (~ two-fold increase, Figs. 2D,E and EV2B–E). Importantly, each treatment produced a distinct molecular profile (Figs. 2B–E and EV2A–K), and impacts synapses differently (Figs. 2F,G and EV2I) along with spontaneous network activity measured by imaging neuronal calcium dynamics (GCaMP6 response) (Fig. 2I,J; Appendix Fig. S2A–C; Movie EV1).

BSI had no strong effect on synapse size but slightly altered calcium transients in individual neurons (Fig. 2F–J; Appendix Fig. S2A–C). The lower synaptic APP-CTFβ levels correlated with decreased colocalization of APP-CTF species with vGLUT1 (Figs. 2H and EV2J,K). On the other hand, GSI was associated with ~20% increase in excitatory synapse size (vGLUT1-positive) without affecting inhibitory synapses (vGAT-positive) (Figs. 2F,G and EV2I). To ensure that excitatory synapse enlargement was due to APP-CTFβ accumulation and/or Aβ decrease and not to another substrate of *γ*-secretase, we repeated this experiment while knocking down APP using siRNA (Appendix Fig. S7). The effect was not observed in neurons transfected with siRNA targeting APP, indicating that APP-CTFβ accumulation and/or Aβ decrease are driving the increase in excitatory synapse size (Appendix Fig. S7C,H; GSI alters synapse number in the absence of APP). The large vGLUT1 presynaptic boutons observed after GSI had elevated levels of APP-CTFβ when compared with control conditions (Figs. 2F,H and EV2J–O). Using puromycin to label ongoing protein synthesis, we also found an increase in protein synthesis after GSI (Appendix Fig. S3). Since only excitatory synapses were affected by GSI, we predicted an excitatory/inhibitory (E/I) imbalance that should drive the network of neurons toward hyperexcitation. Indeed, when recording calcium transients after 4 h of GSI, we observed an increase from 1 to almost 3 calcium transients per minute (Fig. 2I,J). While APA still led to a slight increase in calcium transient frequency, mild compared to GSI, we observed no significant change in ∆F/F0 (signal to baseline) intensity of those transients (Fig. 2I,J; Appendix Fig. S2C). When compared to GSI, APA had strikingly opposite effects, impacting inhibitory synapses while leaving excitatory synapses unaffected (Figs. 2F,G and EV2I). Altogether, our results indicate differential effects on the E/I imbalance of GSI and APA. While accumulation of APP-CTFβ in Aβ-reduced conditions is associated with increased network activity, overproduction of Aβ seems to decrease network activity (Movie EV1).

**Figure 3:**
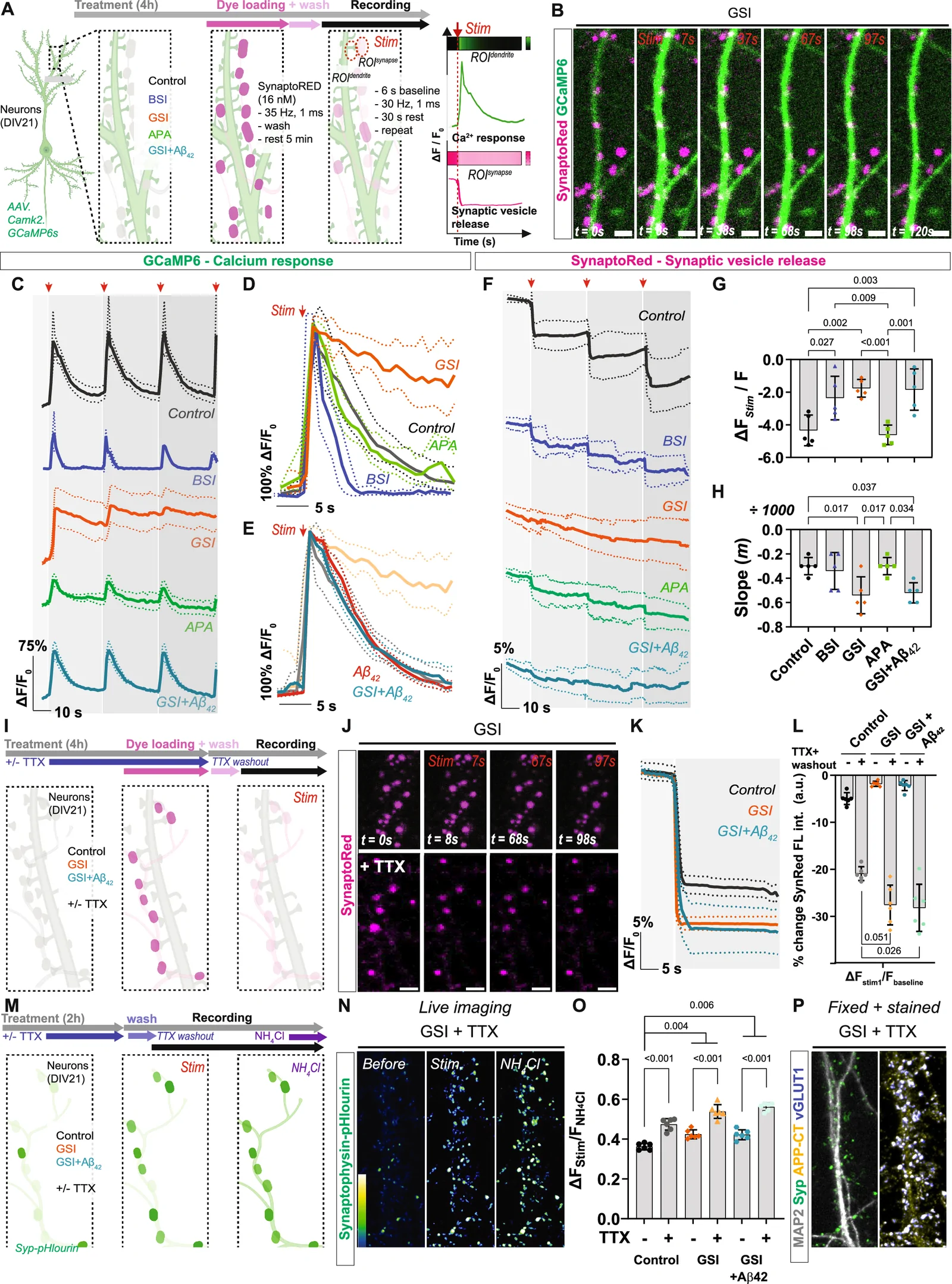
APP and its proteolytic fragments have distinct *yet* interconnected effects in regulating neuronal network activity. (**A**) Schematic representation depicting experimental flow for dual imaging of neuronal Ca^2+^ response and synaptic vesicle release events. Neurons expressing AAV.Camk2.GCaMP6 (DIV3) were subjected to treatment with different modulators (DIV21). Post treatment, synaptic vesicles were loaded with SynaptoRed (SR), excess dye was washed off, and neurons were rested for 5 min for stable baseline recordings. Neurons were stimulated with 30 Hz, 4 repetitions, starting 7th s, with 30 s recovery period. Data was recorded for ~2 min with 125 ms intervals. (**B**) Representative snapshots showing changes in GCaMP6 and SR response in neurons treated with GSI, post-respective stimulation train; snap time, black, stimulation time point, red. (**C**–**H**) Changes in fluorescence intensity of GCaMP6 presented as neuronal Ca^2+^ response (**C**–**E**) and SR as synaptic responses (**F***,* quantified from multiple selected ROIs) were quantified and depicted as line plots, respectively; mean (bold) and SEM (dotted lines), *n* = 15, *N* = 5. (**G**) % Decrease in fluorescence intensity of SR (ΔF/F) depicting synaptic vesicle release (just after first stimulation) has been shown as a bar graph. Bar plots represent mean ± SD; *n* = 15, *N* = 5. (**H**) SR fluorescence values recorded during the initial 6 s before stimulation were linear fitted, slopes calculated, and plotted as bar graphs. Bar plots represent mean ± SD; *n* = 15, *N *= 5. (**I**) Schematic depicting experimental paradigm for SR-only imaging of control untreated, GSI (4 h) ± Aβ (5 min) in the absence or presence of TTX (500 nM) applied simultaneously. (**J**) Representative images depicting the change in SR fluorescence response (magenta) in the absence and presence of TTX ( + TTX); Scale bar = 5 μm, *N* = 3. (**K**) Line plots depicting the % change in SR fluorescence response post first stimulation in respective conditions following TTX washout; bold lines, mean; dotted lines, SD, *N* = 3. (**L**) Bar plots depicting % change in SR fluorescence intensity after first stimulation (normalized to the baseline, ΔF_stim1_/F_baseline_) for the tested conditions. Bar plots represent mean  ± SD; *n* = 6, *N* = 3. (**M**) Schematic representation and timeline for imaging synaptic vesicle exocytosis using Synaptophysin(Syp)-pHlourin sensor. Transfected neurons were used either untreated (control) or treated with GSI (2 h) ± Aβ (5 min) in the presence or absence of TTX (500 nM); subjected to the similar stimulation protocol described above. (**N**) Representative images depict snapshots before stimulation, immediately after stimulation, and post NH_4_Cl quenching; Scale bar = 10 μm, *N* = 3. (**O**) Bar plots represent the activity-evoked pHluorin fluorescence normalized to the total fluorescence measured following NH₄Cl quenching. Bar plots represent mean ± SD; *n* = 6, *N* = 3. (**P**) Neurons post-live imaging were fixed and subjected to immunocytochemistry to examine the localization of APP-CT signals (clone: 140-rb, yellow) and vGLUT1 (blue), in neurons co-stained with MAP2 (gray) and synaptophysin (green), respectively. Scale bar = 10 μm, *N* = 2. Source data are available online for this figure.

To further examine the mechanisms by which local APP-CTFβ and Aβ affect E/I balance at the network level, we conducted live imaging experiments during which we measured in parallel calcium transients and synaptic vesicle release using SynaptoRed under stimulation conditions (Fig. 3A; Appendix Figs. S4–S7 and Movies EV2–4). The entire culture was bath-stimulated using an external electrode at intervals of 30 seconds. Whole-neuron and dendritic calcium transients (Fig. 3A–E; Appendix Fig. S4A,B and Movies EV2 and 3) were consistent with the general trend of our findings recording spontaneous calcium activity (Fig. 2I,J; Appendix Fig. S2 and Movie EV1). Nonetheless, BSI, which did not affect whole-neuron spontaneous calcium transients, resulted in a sharp reduction in the peak amplitudes (Fig. 3C; Appendix Figs. S4A,B and S5A) and decay times (Fig. 3D) of individual stimulation-induced transients. Interestingly, under GSI conditions, transients’ decay-time became so slow that GCaMP6 calcium responses appeared not to go back to baseline between two consecutive stimulations (Fig. 3B–D; Appendix Figs. S4A,B and S5A). Since amyloidogenic APP proteolysis appears to take place in excitatory boutons, we investigated the impact of the modulation of APP proteolysis on presynaptic function. SynaptoRed (SR) integrates into membranes and accumulates into synaptic vesicles (Iwabuchi et al, 2014). After it is washed from the cell media and in the absence of stimulation, SR fluorescence slowly decays in synapses as labeled synaptic vesicles fuse with the active zone membrane. Under control conditions, SR fluorescence decreases immediately after stimulation (~ 5%; Fig. 3A,F,G; Appendix Fig. S4C,D and S5B). Surprisingly, under GSI, SR fluorescence decay was independent of external stimulation (Fig. 3B,F,G; Appendix Fig. S4C,D and S5B), as depicted with the steeper slope of SR fluorescence baseline before the start of the stimulations (Fig. 3H). Here, stimulations failed to produce the normal evoked drop in SR fluorescence as seen with the control or other treatment conditions. These observations imply that GSI-induced neuronal network hyperactivity is due to an increased synaptic vesicle release probability (Pr), such that after 4 h of treatment, the readily releasable pool of synaptic vesicles could be depleted (Appendix Figs. S4C,D and S5B). To assess this, we repeated SR experiments in the presence of Tetrodotoxin (TTX) to suppress evoked vesicular release during the 4 h of GSI treatment (Fig. 3I, *scheme*). In control conditions, stimulation after TTX washout resulted in ~20% decrease in SR signal. In GSI-treated neurons, the evoked SR decrease was only ~2% without TTX treatment but increased to ~25% immediately after TTX washout (Fig. 3I–L; Appendix Fig. S6A,B). In fact, the evoked SR decrease is stronger after TTX washout in GSI-treated neurons compared to control (Fig. 3K,L). This suggests that GSI-associated molecular changes in excitatory presynapses increase Pr, and supports the hypothesis of a depleted presynaptic vesicle pool during the 4 h GSI protocol.

Furthermore, to confirm the specificity of the effects induced by amyloidogenic APP products, we also treated our culture with alpha secretase modulator GI25423X (ASM) and Notch signaling inhibitor FLI-06 (NSI, Fig. EV2A–I). The effect on calcium transients and presynaptic vesicle release (Appendix Figs. S2D and S5E–I) induced by either treatment could be attributed to alternative signaling pathways (metalloproteinases and Notch), plausible independent of APP proteolysis. In addition, compound E, another γ-secretase inhibitor (GSI2), showed comparable results to DAPT (GSI), validating our previous observations (Fig. EV2A–I; Appendix Figs. S2D and S5E–I). Importantly, GSI-induced effects on neuronal and synaptic activity were absent in APP-deficient neurons (Appendix Fig. S7C–G). Altogether, this strongly supports an APP-dependent effect on presynaptic vesicle release. However, are the observed effects due to the accumulation of APP-CTFβ or to the reduction in Aβ?

### Compensatory effects of Aβ_42_ on APP-CTFs/β-associated phenotypes

We observed that 4 h of GSI increases APP-CTFβ concentration and decreases the extracellular Aβ (>50%) (Fig. 2B–E). To further examine the relative contribution of APP-CTFs/β and Aβ, we exposed GSI pre-treated neurons with Aβ_42_ preparations enriched in monomeric species (see “Methods”) extracellularly applied acutely for 5 min, before recording calcium transients and vesicle fusion again using SR. Notably, Aβ_42_ treatment attenuated the effects of GSI on calcium transients and reduced neuronal network hyperactivity to normal/control levels (Fig. 3C,E; Appendix Fig. S5A,B). Despite a slight decrease in ∆F/F0 intensity, most parameters were comparable to control untreated/Aβ_42_ alone treated neurons (Fig. 3E; Appendix Fig. S5A and Movie EV4). However, here Aβ_42_ did not significantly affect synaptic vesicle phenotypes associated with APP-CTFs/β accumulation at the presynapse (Fig. 3F–H; Appendix Fig. S5B). We assessed the effect of Aβ_42_ alone and found that it did not significantly alter the intensity of the calcium transients nor affect their shapes (Fig. 3E; Appendix Fig. S5A,E,G–I and Movie EV4), comparable to recent findings that Aβ effects on synaptic function are concentration- and context-dependent (Siddu et al, 2025).

To confirm our results, we measured vesicle fusion dynamics using another complementary assay—pHluorin, wherein sensor fluorescence increases upon vesicle fusion due to pH changes (physiological pH, Fig. 3M, *scheme*; 3N,O; Appendix Fig. S6C–F). Using pHluorin, we also observed elevated vesicle fusion probability after GSI-evoked pHluorin responses were increased compared to controls (Fig. 3N–O; Appendix Fig. S6E,F). Additionally, post-imaging immunostaining showed accumulation of APP C-terminal species within vGLUT1-positive boutons (Fig. 3P; Appendix Fig. S6D). Importantly, Aβ had no effect on APP-CTFβ-induced increased presynaptic function (Fig. 3N,O; Appendix Fig. S6D–F). Together, SR and pHluorin data indicate that GSI-induced APP-CTFβ accumulation is associated with increased synaptic vesicle release, which within 4 h leads to the depletion of the readily releasable pool-consistent with the observed network hyperexcitability. In sum, acute reduction of γ-secretase activity leads to synaptic accumulation of APP-CTFβ, enlargement of excitatory presynaptic boutons, and increased synaptic vesicle release, thus leading to a pronounced network hyperexcitation. These observations support a presynaptic role for APP-CTFβ in regulating neurotransmitter release and disrupting E/I balance.

**Figure EV3:**
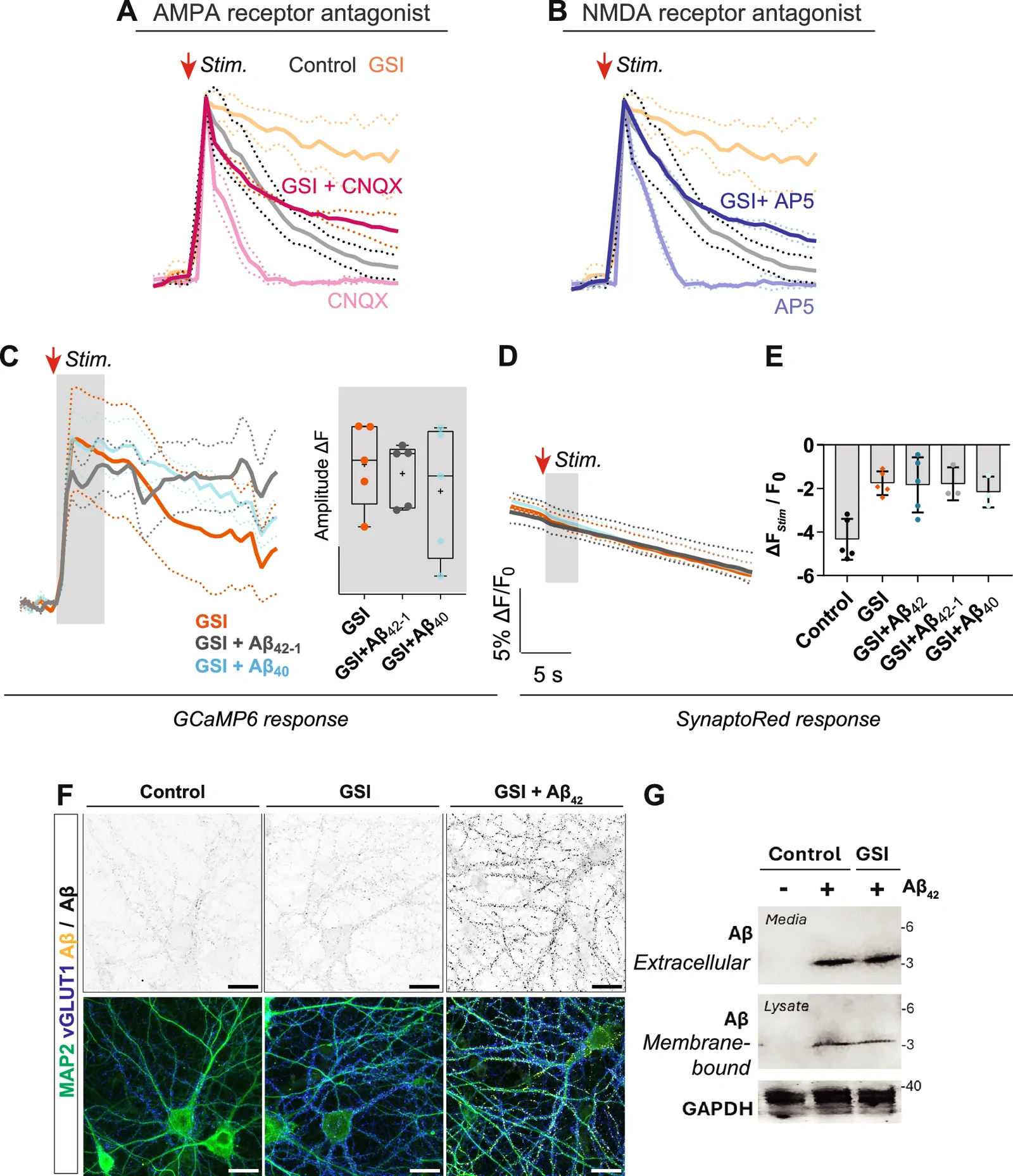
Effects of post-synaptic receptor antagonism and Aβ42 binding to neuronal membranes. (**A**, **B**) Neurons expressing Camk2-GCaMP6s were treated with GSI (4 h) were further treated with CNQX (10 μM, 30 min; blocking post-synaptic AMPA receptors), or AP5 (5 μM, 30 min, post synaptic NMDA receptor antagonist) to examine whether changes at postsynaptic signaling influence GSI-induced network hyperactivity. AMPA and NMDA receptor antagonists were added 30 min before the imaging experiments concluding the GSI treatment time point. Experimental values were compared to neurons treated with the inhibitors alone (without GSI) for the respective durations. Traces (**A**, **B**) depict mean (bold) and SEM (dotted lines). *n* = 6, *N* = 3. (**C**–**E**) As observed in Fig. 3, exposure to 100 nM of Aβ_42_ (5 min) results in rescue of GSI-induced network hyperactivity. In addition, in a similar experimental setting, we examined Aβ_40_ as well as Aβ_42-1_ (reverse sequence, control peptide), and recorded changes in calcium (**C**) and SR fluorescence response (**D**, **E**), *n* = 5, *N* = 2. Here, no significant change in GCaMP6s response, amplitude of the Ca^2+^ transient, or change in SR fluorescence intensity was detected. (**F**, **G**) Control or GSI-treated or untreated (control) neurons additionally treated with or without hAβ_42_ (100 nM, 5 min) were further analyzed *via* immunocytochemistry (**F**) or western blotting (**G**) with an antibody against Aβ epitope. Neurons were further co-stained with antibodies against vGLUT1 (excitatory synapses, blue) and MAP2 (dendrites, green). hAβ_42_ is mainly detected extracellularly bound to the neuronal membranes. Scale bar = 10 μm. Western immunoblot analyses of protein-enriched samples show that Aβ_42_ in the treatment media and bound to neuronal membranes is monomeric in nature. Also, GSI-induced APP-CTF accumulation did not impact the binding of Aβ_42_ to neuronal membranes. GAPDH was used as a loading control. *N* = 2. ns (*P* > 0.05), *(*P* ≤ 0.05), **(*P* ≤ 0.01), ***(*P* ≤ 0.001); one-way ANOVA. Source data are available online for this figure.

Application of monomeric Aβ_42_ preparations (see “Methods”) can restore acute basal network activity in GSI-treated neurons. These effects were observed following short-term application and may reflect acute, context-dependent actions of Aβ on synaptic activity. Many reports have shown that Aβ regulates neuronal activity by binding to postsynaptic glutamate receptors (Campos-Peña and Meraz-Ríos, 2014; Proctor et al, 2011; Dinamarca et al, 2012; McGeachan et al, 2025). Supporting these observations, we could mimic acute Aβ treatment effects on GSI-treated neurons by shortly exposing them to CNQX (AMPA receptor antagonist, Fig. EV3A) or AP5 (NMDA receptor antagonist, Fig. EV3B). Of note, this effect was only seen with Aβ_1-42_ species, wherein Aβ_1-40_ and Aβ_42-1_ did not show this effect for the acute 5-min acute-treatment timeframe examined in this study (Fig. EV3C–E). ICC and WB analyses post-acute exposure of Aβ_42_ to untreated or GSI pre-treated neurons showed that Aβ_42_ peptides remain unaggregated and bound to the neuronal membranes, wherein negligible signals are seen in the juxtanuclear space, independent of GSI pre-treatment (Fig. EV3F,G).

### Presynaptic APP-CTFβ interacts with synaptic vesicle release machinery

**Figure 4:**
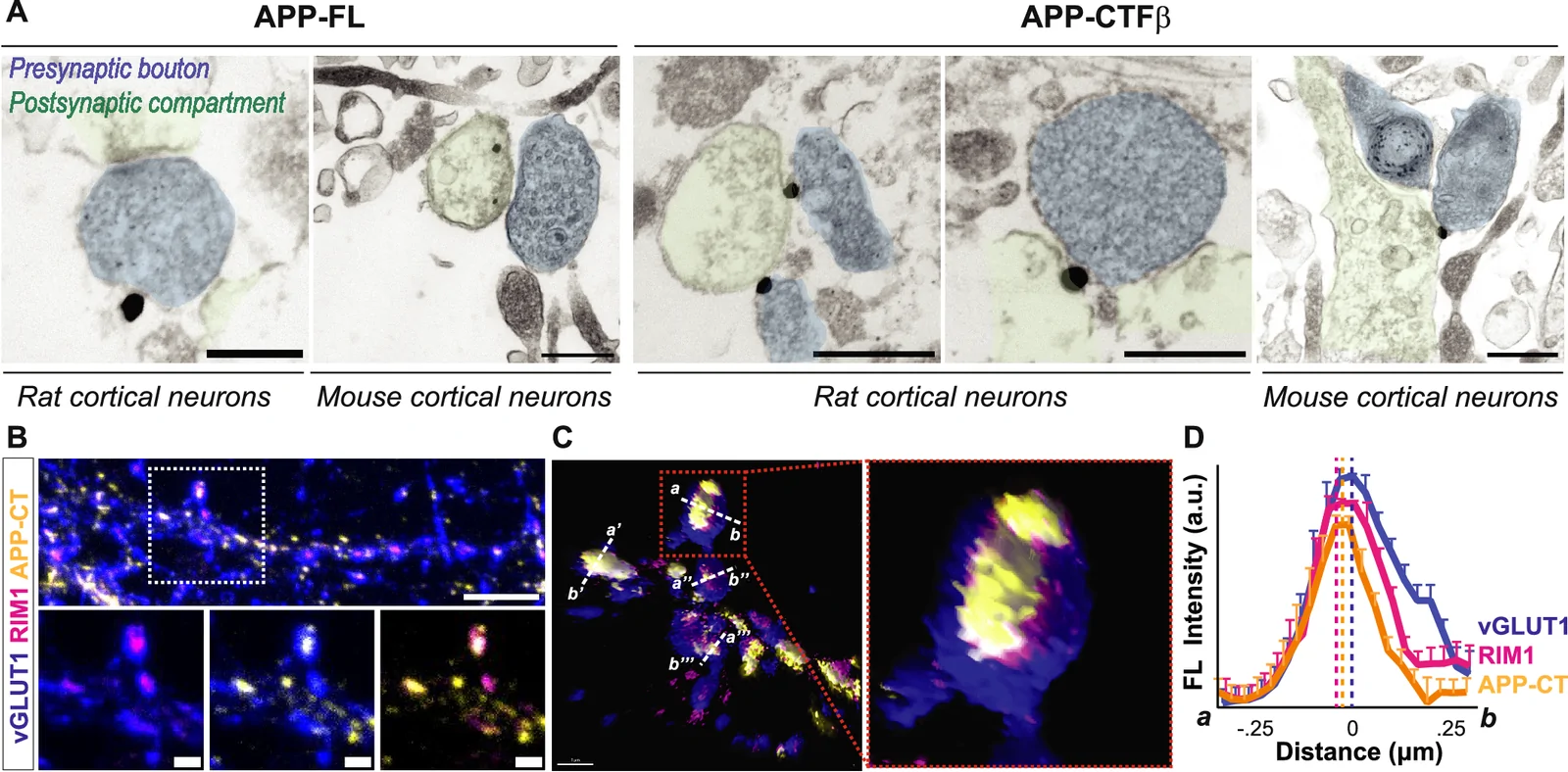
Presynaptic APP-CTFβ is present in the active zone. (**A**) Electron micrographs of immunogold-labeled full-length APP (APP-NT, detected with antibody AB#3) and β-C-terminal fragments (APP-CTFβ, antibody clone: 82E1) at synapses in cultured rat and mouse cortical neurons. Presynaptic boutons, blue; postsynaptic counterpart, green. Scale bar = 300 nm, *N *= 2. (**B**) Immunocytochemical analysis depicting colocalization between APP C-terminal species (clone: C1/6.1) and presynaptic active zone protein RIM1; scale bar = 5 μm; zoomed-in image, white dotted box, scale bar = 1 μm; *N* = 3. (**C**) Imaris3D reconstruction depicting presynaptic APP C-terminal species is detected in the RIM1-enriched active zone. (**D**) Line plot depicting distribution of APP-CTF and RIM1 signals within vGLUT1-positive boutons. Data represent mean ± SD from four ROIs and are representative of three independent experiments. Source data are available online for this figure.

Experimental evidence from excitatory synapses, calcium recordings, and synaptic activity pinpoints that APP and APP-CTFs/β are functionally associated at the presynapse. We therefore examined if there are key differences in the synaptic location of APP-FL and APP-CTFs/β. We performed immunogold electron microscopy (EM) after live-labeling with antibodies targeting the APP-FL ectodomain (detected with an APP-NT antibody, #AB3) and APP-CTFβ epitopes (detected with antibody clone: 82E1) in rat and mouse cortical neuronal cultures (Fig. 4A; Appendix Fig. S8A). APP-CTFβ was more frequently detected at the presynaptic membrane, particularly within the active zone, compared to full-length APP. This was further validated by ICC of cultured neurons, wherein presynaptic APP-CT species colocalize with the active zone protein RIM1 (regulating synaptic membrane exocytosis protein 1, Fig. 4B–D). What is the function of APP-CTF at the active zone? Does it interact with other synaptic proteins?

**Figure 5:**
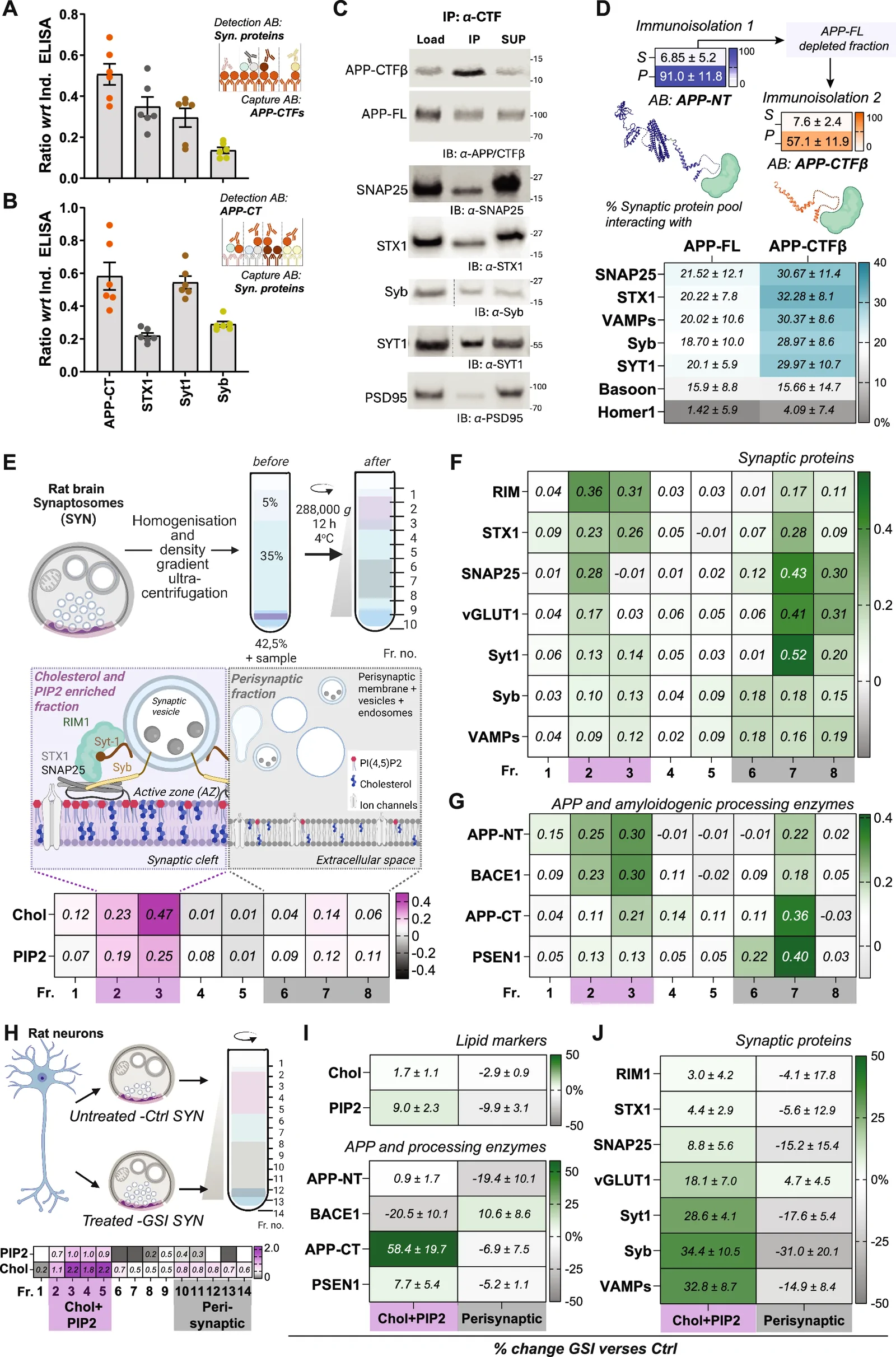
Presynaptic APP-CTFβ interacts with vesicle release machinery and facilitates docking of synaptic vesicles at the active zone. (**A**, **B**) Quantitative analyses from in-house sandwich ELISA showing interaction of APP C-terminal with different synaptic proteins examined from synaptosome homogenate. Bar plots represent mean ± SEM; *n* = 6, *N* = 3. The inset scheme depicts the capture (APP-CT, clone: 140-rb, **A**; STX1/Syt1/Syb, **B**) and detection AB (STX1/Syt1/Syb, **A**; APP C-terminal species, clone: C1/6.1, **B**) used in the experiments. (**C**) Rat synaptosome homogenate was immunoisolated with α-APP-C terminus antibody, and interacting proteins were detected *via* western immunoblotting. *N* = 3. (**D**) ELISA analyses dual immunoisolation from rat synaptosome homogenate, first with α-APP-N terminus antibody (AB#3) and later with α-APP antibody (clone: M3.2, to specifically pull-down APP-CTFβ) depict % relative abundance of synaptic proteins interacting specifically with the C-terminal domain of APP-CTFβ and compare it to that of the full-length protein. *n* = 6, *N* = 3. (**E**, **H**) Schematic representation depicting fractionation of synaptosomal membranes into cholesterol and PI(4,5)P2-enriched (highlighted in magenta) and the perisynaptic membrane fraction (grays) from rat brain (**E**) and cultured neurons (**H**). The heatmap depicts the normalized levels of individual lipid in each fraction. *n* = 8, *N* = 4. (**F**, **G**) Heatmaps depicting the relative pool of APP-FL (AB#3), APP C-terminal species (clone: 140-rb), and its processing enzymes (**F**), synaptic proteins (**G**), as well as within isolated fractions. *n* = 8, *N* = 4. (**H**–**J**) Cultured rat neurons were either treated with GSI (DAPT, 10 µM, 4 h) or untreated and subjected to synaptosome isolation and lipid fractionation. Collected fractions 1–14 were analyzed for cholesterol and PIP2 content (heat map, **H**). Heatmaps (**I**, **J**) depict the percentage change of lipids, APP (AB#3), APP-CT species (clone: 140-rb), and processing enzymes (**I**) and different synaptic proteins (**J**) in the two distinct pools. *n* = 4, *N* = 2. ns (*P* > 0.05), *(*P* ≤ 0.05), **(*P* ≤ 0.01), ***(*P* ≤ 0.001); one-way ANOVA. Source data are available online for this figure.

APP has been shown to interact with SYT1 (synaptotagmin-1) *via* its intracellular luminal C-terminal domain (Kohli et al, 2012; Del Prete et al, 2014; Groemer et al, 2011; Gautam et al, 2015; Guénette et al, 2017; Barthet et al, 2018). Using sandwich ELISA (Fig. 5A,B) and immunoisolation (WB, Fig. 5C) techniques, along with SYT1, we additionally identified STX1 (syntaxin 1), Syb (synaptobrevin 1), SNAP25 (synaptosomal-associated protein 25) as interactors of the presynaptic APP C-terminal domain within synaptosomes isolated from adult rat brain. Here, again, because APP full length and APP-CTFs share the same intracellular C-terminal domain (Fig. 5C), they are indistinguishable in proteomics and mass spectroscopy methods. However, specific antibodies immunoreact with APP full length and APP-CTFs/β (Appendix Fig. S16 and Appendix Table S2). Thus, we set out to examine if there are any differences between the two, using a dual-immunoisolation-based ELISA technique (Fig. 5D, *scheme*; Appendix Fig. S8B-J). We primarily enriched the full-length protein using an antibody against the ectodomain (APP N-terminal, APP-NT; Appendix Fig. S8B). The APP-FL depleted material was then enriched for APP-CTFβ (clone: M3.2, Appendix Fig. S8B), and the levels of synaptic vesicle proteins were then compared between the two fractions (Fig. 5D, *heatmap*; Appendix Fig. S8C–J). Quantitatively, these results depict that synaptic vesicle proteins preferentially interact with APP-CTFβ (>1.5-fold enrichment) as compared to the full-length protein.

The next question we asked, does the localization of APP-CTFs at the active zone and its interaction with synaptic vesicle proteins influence vesicle docking? We examined this by homogenizing synaptosomes and separating lipid domains using density gradient ultracentrifugation (Fig. 5E). We validated lipid fractionation by probing the specific enrichment of lipids and proteins in the various fractions. As expected, cholesterol (Chol) and PIP2 (Fig. 5E, *heatmap*) and FLOT-1 (flotillin) were mainly found together and in fractions 2 and 3 (Appendix Fig. S8K,L), while TFR (transferrin receptors, Appendix Fig. S8K,L) was enriched in fractions 6–8. Enrichment of RIM1 and STX1 within fractions 2–3 indicated that Chol and PIP2-containing fractions are relatively enriched in active zone membranes. SNAP25, vGLUT1, SYT1, Syb, and VAMPs (vesicle-associated membrane proteins) are significantly enriched in TFR-positive fractions (Fig. 5F; Appendix Fig. S8K,L). Thus, we consider the large synaptic vesicle pool, as opposed to the docked vesicles at the active zone (fractions 2–3), to be part of the perisynaptic fraction (fractions 6–8). Interestingly, the levels of APP-FL and APP-CT species show differential distribution between the two fractions, a comparable distribution to their immediate cleaving enzyme -BACE1 and PSEN, respectively (Fig. 5G). Of note, APP-CTFs/β are known to be primarily detected in vesicular endo-lysosomal membranes (Bretou et al, 2024; Luo et al, 2025; Tian et al, 2014; Lauritzen et al, 2019b, 2019a), which are enriched within the perisynaptic fraction (Fig. 5G). This suggests that APP-CTFs are a transient resident of the active zone. Next, we isolated synaptosomes from untreated (control) and GSI-treated neurons (Fig. 5H, *scheme*) and used a similar protocol to separate Chol and PIP2-enriched active zone and perisynaptic membrane fractions (Fig. 5H, *heatmap*; Appendix Fig. S8M). Comparing the protein levels between GSI-treated and untreated conditions, we observed an approximately 60% increase in APP-CT immunoreactivity within the chol+PIP2-enriched active zone fraction, while APP-FL levels remained unchanged (Fig. 5I; Appendix Fig. S8N), consistent with our previous observations. Notably, synaptic vesicle proteins—Syt1, Syb, and VAMPs, that interact with APP C-terminal domain, showed a ~ 30% increase in the active zone fraction, whereas levels of RIM1, STX1, and SNAP25 were comparable across conditions (Fig. 5J; Appendix Fig. S8O). These findings suggest that the accumulation of APP-CTFβ at the active zone enhances the pool of docked synaptic vesicles. Yet, the question remains: how does the accumulation of APP-CTFβ directly enhance synaptic vesicle release?

### APP-CTFβ oligomerization is associated with an increase in vesicle release probability

**Figure 6:**
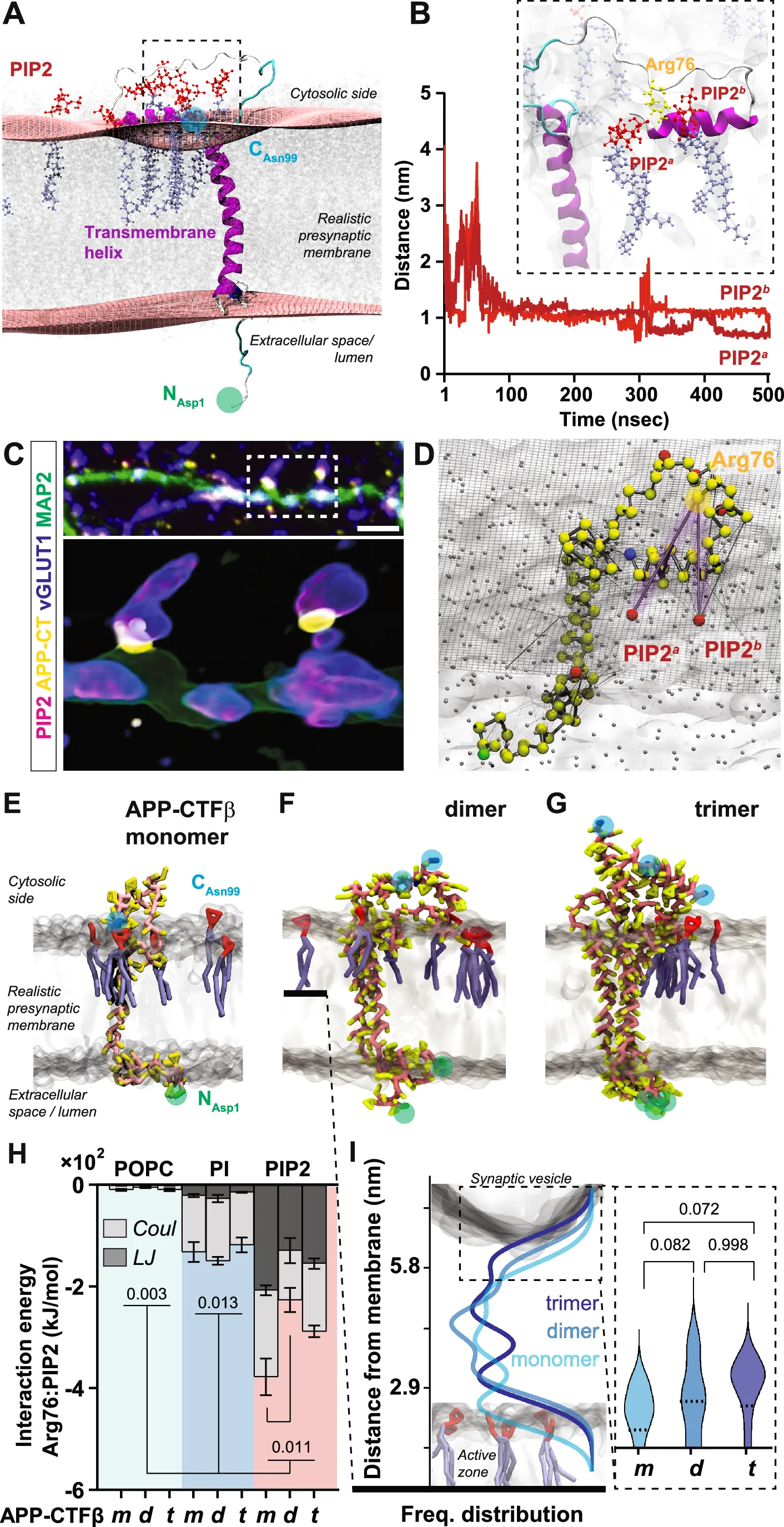
Interaction between Arg76 of APP-CTFβ and PIP2 head groups facilitates its C-terminal cytosolic exploration. (**A**, **B**, **D**) All-atom MD simulation of APP-CTFβ (C99) in a membrane that approximates the realistic presynaptic lipid concentrations (Appendix Table S5; **A**, snapshot). The surface of each monolayer was obtained by fitting the phosphorus atom positions of each lipid to an analytical function (Chol not used in the fitting). The protein is shown in secondary structure representation, and motifs are colored, respectively. α-helix, purple*;* turns, cyan; coils, white. N-terminal Asp1, green; C-terminal Asn99, blue; PIP2 head group, red; and tails, blue. (**B**) The distance between the PO_4_ headgroup of the two closest PIP2 head groups and the furthest carbon atom (C*Z*) in the side chain of Arg76 as a function of time. The results were consistent across three independent replicas. A simulation snapshot depicting the interaction of PIP2 with Arg76 of APP-CTFβ. (**C**) Immunocytochemical analysis and Imaris3D reconstruction depicting presynaptic APP-CTF (clone: 140-rb, yellow) colocalizes with PIP2 (magenta). Neurons were co-stained with MAP2 (dendrites, green) and vGLUT1 (excitatory presynapse, blue). The inset depicts a zoom-in (red dotted box), *N* = 2. (**D**) Interaction energy network mapped onto the all-atom structure. Every residue is shown as its center of geometry. The width of a connection correlates to the strength of the non-bonded interaction energies. Arg76 (yellow circle), two closest PIP2 (red) Arg76:PIP2 interaction (highlighted in light red). (**E**–**I**) Coarse-grained MD simulation of 1, 2, or 3 APP-CTFβs in a realistic presynaptic membrane. Renderings of monomer (**E**), dimer (**F**), and trimer (**G**) APP-CTFβ in the elastic network model; protein backbone, light orange; side chains, yellow; PIP2 head group, red; tails, light blue; N-Asp1, green, and C-Asn99 terminal, blue, analogous to (**A**). (**H**) Total average interaction energy contributions of Arg76 to all lipids of a specific type, split into Van der Waals (LJ, Lennard-Jones) and electrostatic (Coul, Coulomb) contributions. The contributions have been scaled/averaged by the abundance of the lipids. Note, since some of the PIP2 lipids are located far from Arg76, the interaction energy with the bound PIP2 is higher. Statistical analysis compared between the lipid types, two-way ANOVA, interaction of Arg76 with PIP2, bar plots represent mean ± SD; *N* = 3; two-way ANOVA, *P ≤ 0.05, **P ≤ 0.01. (**I**) Line plots show the height of the last backbone bead of the C-terminal to/from the center of the membrane leaflet as obtained from a fitting of the PO_4_ headgroup for all simulation models. Distance between the active zone and synaptic vesicle membranes depicted as a cartoon representation. The inset depicts the cumulative distribution of the C-terminal last backbone bead, between the distances of 5.5 nm and above, one-way ANOVA. All data are representative of three independent simulation runs (three replica simulations), depicted in Appendix file. Source data are available online for this figure.

To further expand on the presynaptic role of APP-CTFβ and understand the link between its accumulation at the excitatory synapse and increase in release probability, we modeled human APP-CTFβ (or C99) for the first time in a realistic presynaptic membrane context, approximating lipid concentrations at presynapse (Binotti et al, 2021; Lauwers et al, 2016) (Fig. 6A; Appendix Table S5 and Movie EV5), or POPC membranes, and performed all-atom molecular dynamics (MD) simulations for 500 ns. The simulations indicate that the transmembrane domain (TMD, residues 28-52) remains suspended within the membrane bilayer, the N-terminal arm remains unstructured, wherein the C-terminal helix dips and remains at the membrane surface (Appendix Figs. S10 and S11), consistent with previous works employing simplified lipid bilayers (Pantelopulos, 2022; Pantelopulos et al, 2018, 2022). Our interest was piqued to notice that Arg76 interacted with PIP2 head groups (stoichiometry of 1:2), only present in realistic presynaptic membranes, within the first 100 ns of the simulations (Fig. 6B, replicas 2 & 3 in Appendix Fig. S12 and Movie EV5). For all our simulations, this interaction remained stable throughout when established (Fig. 6B; Appendix Fig. S12 and Movie EV5). Immunostained primary neurons show the co-presence of APP-C-terminal species and enriched PIP2 lipids at the active zone (facing the dendritic arm) of vGLUT1-positive excitatory presynapse (Fig. 6C). These experimental in vitro observations support in silico findings that APP-CTFβ and PIP2 are present in close proximity in physiological conditions. The strength of this interaction was examined by computing the interaction energy, indicating the strength of the non-bonded interactions (Fig. 6D). Strong energetic non-covalent bonds are measurable throughout the protein between not-bound adjacent residues (amino acid position ±2). Notably, the interaction energy between Arg76 (yellow circle) and the two closest PIP2 head groups (*red circles*) is comparable to the observed intramolecular interaction within APP-CTFβ (Fig. 6D; Appendix Fig. S13A). Thus, we hypothesize that the Arg76-PIP2 head group interaction is relevant in regulating the relative position of the C-terminal helix of APP-CTFβ toward or away from the inner leaflet of the plasma membrane. This could influence its accessibility for binding to synaptic vesicles *via* Synaptotagmin-1 or synaptobrevin, as shown previously ((Fig. 5; Appendix Fig. S8) and (Kohli et al, 2012; Del Prete et al, 2014; Groemer et al, 2011; Zoltowska et al, 2017; Gautam et al, 2015; Guénette et al, 2017)).

Recently, there have been reports about the propensity of APP-CTFβ to form dimers or higher-order oligomers (Pantelopulos et al, 2018, 2022; Lauritzen et al, 2019a; Badot et al, 2026; Perrin et al, 2020). To examine this behavior of APP-CTFβ, we additionally employed coarse-grained MD models using the Martini 3 force field (Kroon et al, 2025; Souza et al, 2021; Pedersen et al, 2024) to capture longer dynamics. To model APP-CTFβ secondary structure, we used two popular approaches: (i) the elastic network approach (Periole et al, 2009) and (ii) the GO model (Poma et al, 2017). For both membrane types described above, we introduced 1, 2, or 3 motifs of APP-CTFβ (Fig. 6E–G; Appendix Table S6) in a single membrane created with TS2CG 2.0 (Schuhmann et al, 2025). During the course of 5 μs simulations we observed the spontaneous formation of dimers and trimers of APP-CTFβ (renderings of mono-, di-, and trimers shown in (Fig. 6E–G and Movie EV6)). We computed the interaction energy contributions (Schuhmann et al, 2024) of Arg76 to the different lipid types, i.e., POPC, PI, and PIP2 (Fig. 6H), and other lipids (Appendix Figs. S13B,C and S14). In line with all-atom simulations, we observed for a single motif of APP-CTFβ that Arg76 made the strongest interaction with PIP2. Notably, dimerization as well as trimerization of APP-CTFβ significantly decreased this interaction. This reduction in Arg76-PIP2 interactions further influences the positioning of the C-terminal helix- away from the inner membrane leaflet (Fig. 6E–G, *blue circles*).

Next, we examined the distance of the C-terminal residue (Asn99) of APP-CTFβ to the middle of the bilayer membrane (Fig. 6I; Appendix Fig. S15). During our coarse-grained simulations, we observed that the C-terminal domains of APP-CTFβ fluctuate between locations from within the plasma membrane up to 9 nm from the center of the membrane bilayer, exploring the cytosol. Interestingly, C-terminal domains of APP-CTFβ dimers and trimers spend more time further away from the membrane (Fig. 6I), during the simulations, than the ones of monomers in which Arg76-PIP2 interactions are stronger (Fig. 6H). This is consistent with APP-CTFβ oligomerization increasing the likelihood of C-terminal exposure towards the cytosol (Fig. 6I; Appendix Fig. S15), which may facilitate its interactions with synaptic vesicle proteins, influencing the recruitment of synaptic vesicles to the active zone. Indeed, the distance separating the docked vesicle membrane from the surface of the inner side of the plasma membrane is approximately 5.8 nm (Ogunmowo et al, 2023), within the range of APP-CTFβ C-terminus cytosolic exploration (Fig. 6I, *inset*). These observations raise the possibility that APP-CTFβ oligomerization favors its C-terminal helix cytosolic exploration, which influences synaptic vesicle recruitment and/or fusion. Taken together, these observations suggest that GSI-induced molecular crowding of APP-CTFβ favors its oligomerization. Here, the C-terminal interactions may influence vesicle positioning at the presynapse, consistent with the experimentally observed increase in network activity and enrichment of synaptic vesicle proteins at the active zone.

### APP-CTFβ accumulates and localizes in excitatory synapses in the human AD brain

**Figure 7:**
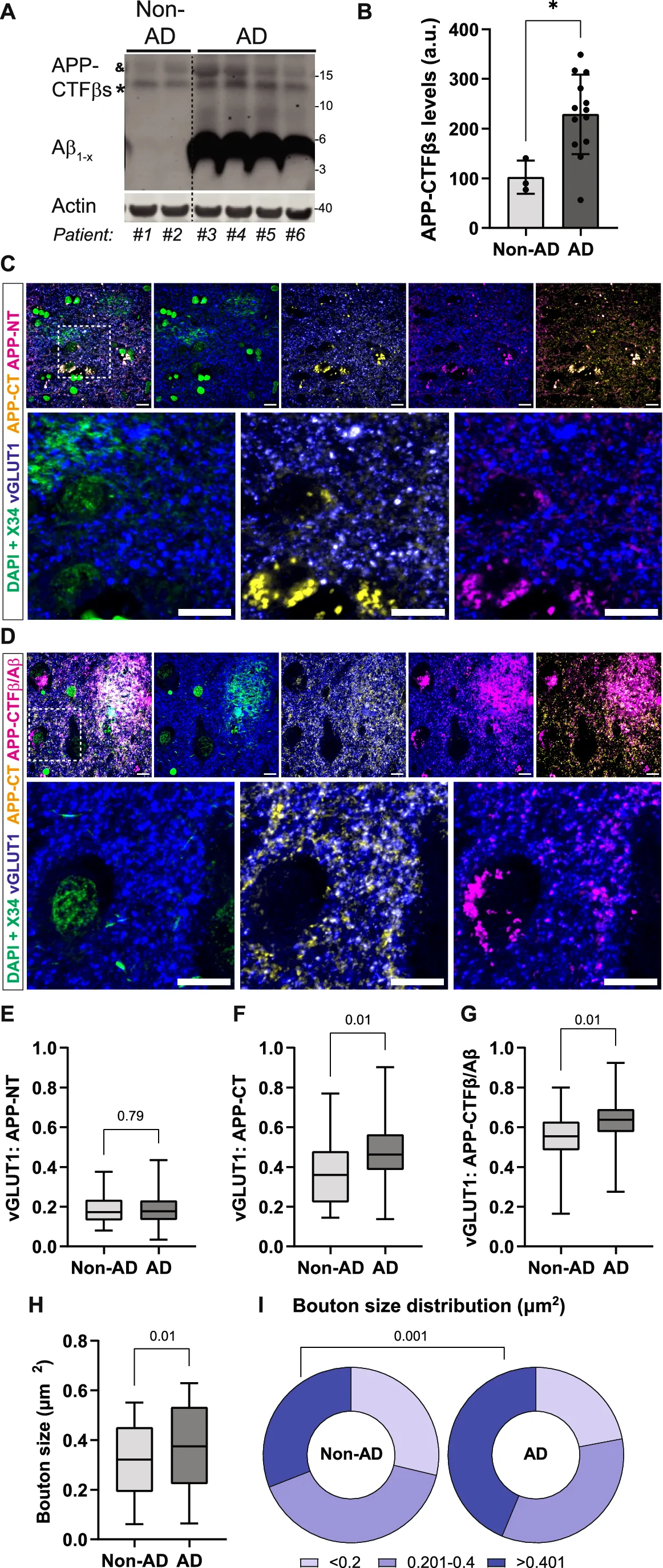
APP-CTFβ accumulates at excitatory synapses in AD patient brains. (**A**) Representative western blot showing non-phosphorylated APP-CTFβ (*&*) and phosphorylated APP-CTFβ species (*), detected with antibody clone: 82E1, in the brain homogenates from non-AD controls (*N* = 2) and AD patients (*N* = 4). (**B**) Quantification of total APP-CTFβ levels normalized to loading control, showing a significant increase in AD patient samples (*N* = 13) compared to controls (*N* = 3). Bar plots represent mean ± SEM. Demographics of the AD patient cohort analyzed have been summarized in Appendix Table S7, age of disease onset of 72 ± 8 years and a mean disease duration of 9 ± 5 years, *N* = 13. (**C**, **D**) Immunohistochemistry of paraffin-embedded fixed postmortem brain sections from AD patients showing APP-CT (clone: 140-rb, **C**, clone: 3125-rb, **D**; yellow) and APP-NT (AB#3, **C**, magenta) or APP-CTFβ/Aβ (clone: 82E1, **D**, magenta) co-stained with excitatory presynaptic marker vGLUT1 (blue) and DAPI (green), with X34 labeling amyloid plaque deposits (green); scale bar = 20 μm, *N* = 2. Details of patients analyzed have been summarized in Appendix Table S7. (**E**–**G**) Box and whiskers plot depicts quantification of colocalization coefficient (Mander’s M2) between excitatory synaptic marker vGLUT1 and APP species: APP-NT (AB#3, **E**), APP-CT (clone: 3125-rb, **F**), and APP-CTFβ/Aβ (clone: 82E1, **G**). (**H**) Distribution of excitatory presynaptic bouton sizes in AD versus non-AD control brains. Center line, median; box boundaries, 5th and 95th percentiles; whiskers extend to the minimum and maximum values; *n* = 2, *N* = 2 independent experiments. (**I**) Circle plot depicting the distribution of presynaptic bouton areas in non-AD versus AD patient samples, *n* = 2, *N* = 2 independent experiments. ns *P* > 0.05, *P ≤ 0.05, **P ≤ 0.01, ***P ≤ 0.001; Welch’s *t* test. Source data are available online for this figure.

**Figure EV4:**
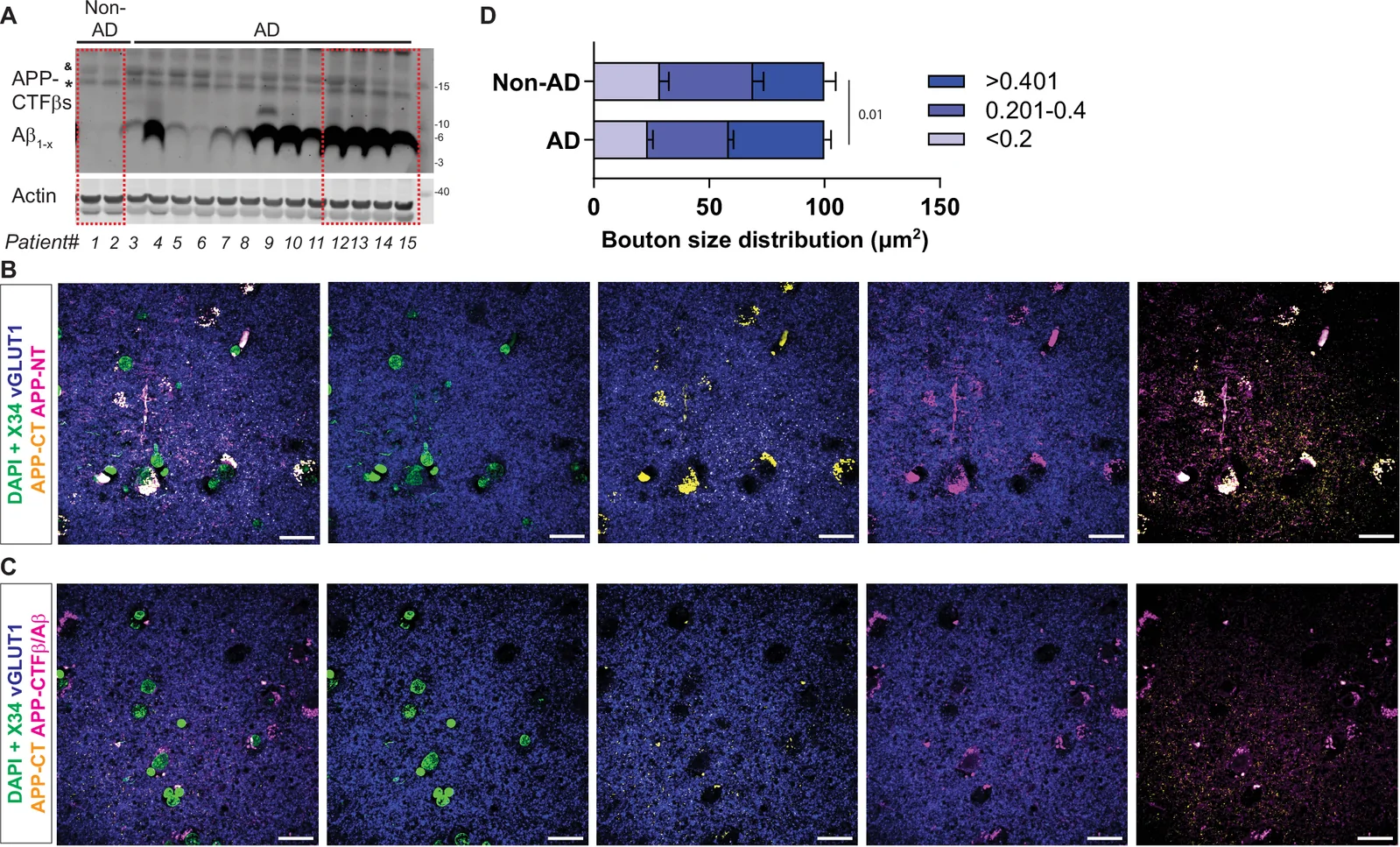
Biochemical and immunohistochemical analysis of patient samples. (**A**) Western blot showing non-phosphorylated APP-CTFβ (&) and phosphorylated APP-CTFβ species (*) in the brain homogenates from non-AD controls (*N* = 2) and AD patients (*N* = 13). Red dotted box represents the cropped immunoblot depicted in Fig. 7A. (**B**, **C**) Immunohistochemistry of paraffin-embedded fixed postmortem brain sections from non-AD patients showing APP-CT (clone: 140-rb, **B**, clone: 3125-rb, **C**; yellow) and APP-NT (AB#3, **B**, magenta) or APP-CTFβ/Aβ (clone: 82E1, **C**, magenta) co-stained with excitatory presynaptic marker vGLUT1 (blue) and DAPI (green), with X34 labeling amyloid plaque deposits if any (green); *n* = 2, *N* = 2 independent experiments. (**D**) Cumulative bar plots depict the distribution of presynaptic bouton areas in non-AD versus AD patient samples, *n* = 2, *N* = 2 independent experiments. Source data are available online for this figure.

Our findings in neuronal cultures suggest a potential role for APP-CTFβ species during AD onset and early stages of the disease. However, as brain donations only occur postmortem, the sample size of the early stage is limited. We thus examined whether signatures of APP-CTFβ dysregulation are detectable in late-stage AD. We examined 13 cases representative of typical late-onset AD, with an age of disease onset of 72 ± 8 years and a mean disease duration of 9 ± 5 years (demographics details in Appendix Table S7 and as described in (Boon et al, 2018, 2020)). Western immunoblotting revealed the presence and significant increase in the levels of APP-CTFβ species in AD patient brains compared to non-AD controls (Figs. 7A,B and EV4A). To further determine whether APP-CTFβ-dependent synaptic alterations observed in vitro, also occur in the human brain, we performed immunostaining on fixed brain sections using markers for excitatory presynapses (vGLUT1). APP C-terminal immunoreactivity, including APP and CTFs (Figs. 7C,D and EV4B,C, *yellow*), but particularly APP-CTFβ/Aβ species (Figs. 7D and EV4C, *magenta*) exhibited a pronounced enrichment at vGLUT1-positive presynaptic boutons in AD patients, whereas APP N-terminal signals showed minimal overlap (Figs. 7C and EV4B, *magenta*). Mander’s overlap coefficient analysis demonstrated that APP C-terminal immunoreactivity (Fig. 7F), and in particular APP-CTFβ (Fig. 7G), exhibited significantly greater colocalization with vGLUT1 than APP N-terminal species (Fig. 7E). Notably, excitatory synapses in AD samples displayed an increase in presynaptic bouton size compared to controls (Figs. 7H,I and EV4D). These data provide critical translational support for our mechanistic observations in rodent/murine neuronal models, indicating that APP-CTFs/β accumulation in the human AD brain is associated with enlargement of excitatory synapses and increased localization of APP-CTFs/β to presynaptic terminals.

## Discussion

Aβ, in its oligomeric form, has been shown to have toxic effects on neuronal and synaptic function (Madhu and Mukhopadhyay, 2021; Cerpa et al, 2008). Notably, the soluble Aβ oligomers can bind to excitatory synapses/neurons (Palop and Mucke, 2010; Klein, 2006; Klein et al, 2008; Viola and Klein, 2015) and induce neuronal hyperexcitability, a phenomenon observed both in animal models and early-stage AD patients (Ray et al, 2024; Anastacio et al, 2022; Tok et al, 2022; Hector and Brouillette, 2020; Li et al, 2024). This aberrant increase in neuronal activity contributes to network dysfunction and impaired synaptic plasticity (Kazim et al, 2021; Sciaccaluga et al, 2021; Papanikolaou et al, 2025; Wilson et al, 2025; Zarhin et al, 2022; Wei et al, 2010). These events furthermore drive Aβ production (Anastacio et al, 2022; Tok et al, 2022; Wilson et al, 1999; Martinsson et al, 2022; Burrinha et al, 2021; Zott et al, 2019) and exacerbate tau pathology (Giorgio et al, 2024; Samudra et al, 2023), creating a vicious cycle of neurotoxicity, neuronal loss, and neuroinflammation (Standridge, 2006; Li et al, 2024). While it is clear that Aβ oligomers contribute to AD progression, the precise role of APP proteolysis in disease onset still remains under investigation. Here, we sought to delineate the roles of APP and its proteolytic fragments, particularly APP-CTFs/β, in synaptic and neuronal function, and their potential contribution to early synaptic alterations in AD onset.

We show that in endogenous conditions, APP and its amyloidogenic processing components are relatively enriched at excitatory presynaptic terminals, in line with previously published transcriptomics and proteomics datasets (Schanzenbächer et al, 2016; Glock et al, 2021; Fornasiero et al, 2018; van Oostrum et al, 2023). Acute pharmacological modulation of APP proteolysis, under endogenous and physiologically plausible range, results in synaptic alterations. Pharmacological treatment with BSI produced modest changes in network activity and selectively reduced synaptic APP-CTFβ, while previous studies reported marked reductions in glutamate release employing genetic BACE1 knockout or prolonged inhibition (Wang et al, 2008; Das et al, 2021; Das and Yan, 2017). APP-CTFβ accumulation induced by GSI is associated with excitatory synapse enlargement, an increase in neurotransmitter release probability, and neuronal network hyperexcitation. In contrast, APA-induced Aβ overproduction resulted in a modest reduction in induced calcium transients. Our results are consistent with literature reports showing chronic GSI treatment (Essayan-Perez and Südhof, 2023), familial mutations of APP (Thordardottir et al, 2017; Marcello et al, 2012), BACE1 (Das et al, 2021; Das and Yan, 2017; Yan et al, 2016), and PSEN1 (Kabir et al, 2020; Lepeta et al, 2016) in disrupting synaptic function. Now, the retention of AICD within membrane-bound APP-CTFβ at excitatory synapses (GSI treatment), rather than its nuclear translocation (APA condition), may influence translation-related processes, contributing to changes in synapse size. These observations raise the possibility of a non-canonical, local translation-linked function of AICD (Müller et al, 2008; Schettini et al, 2010) in early synaptic remodeling during AD onset, which should be investigated in the future.

One of the key observations from our study is that APP-CTFs/β localized at presynaptic active zones, interact with components of the synaptic machinery and local lipids. Previous studies have reported binding between APP C-terminal and synaptic vesicle protein- synaptotagmin using high-end quantitative mass spectrometry and proteomic approaches (Kohli et al, 2012; Del Prete et al, 2014; Groemer et al, 2011; Zoltowska et al, 2017; Gautam et al, 2015; Guénette et al, 2017). However, in these studies, the authors were not able to unambiguously distinguish APP-CTFs/β from APP full length, as they share the same protein sequence (APP-CTFs lack the large N-terminal ectodomain of APP full length), and they used antibodies targeting shared sequences. Interpretation of APP-derived species in the literature has, however, been complicated by the extensive sequence overlap between APP-FL, APP-CTFs, and Aβ species. Antibodies directed against the C-terminus (clone C1.6.1, 140-rb, and 3125) detect APP and its derived C-terminal fragments, but are unable to unequivocally distinguish between the full-length protein and CTFs. The development of N-terminal end-specific antibodies (clone: 82E1) has substantially improved the specificity of APP-CTFβ/Aβ detection (Hunter and Brayne, 2017). This distinction is particularly important as multiple proteolytic products can coexist within the same neuronal compartment. To minimize these limitations, we combined antibodies recognizing distinct epitopes with complementary biochemical and functional approaches to discriminate between APP-FL, APP-CTFs, and Aβ throughout this study. Indeed, we could show that in brain tissue, the interaction with synaptic vesicle proteins Syt1, Syb and VAMPs, is mainly driven by the C-terminal domain of APP-CTFβ, rather than that of the full-length APP, employing simpler ELISA-based immunoisolation and western blotting.

We would also like to point out that in addition to the canonical β-cleavage site, neuronal APP undergoes cleavage at an alternative β′-site, generating β-secretase-derived products that have historically been difficult to distinguish from α-secretase-derived fragments by biochemical methods (Simons et al, 1996; Tienari et al, 1997; Vassar, 2001; Portelius et al, 2010; Yan et al, 2001). APP-CTFβ species are constitutively generated in neurons under physiological conditions, providing a rationale for investigating their contribution to synaptic physiology as well as their contribution during AD onset. When we treated living neurons with an antibody targeting the short extracellular sequence of APP-CTFβ (AB#1, clone: 82E1, Appendix Fig. S9A, *scheme*; S9E, *live-labeled ICC*), we observed a rapid (few min) silencing of neuronal and dendritic calcium traces (Appendix Fig. S9B,C) and a sharp decrease in SR fluorescence responses (Appendix Fig. S9D). This effect was also observed when using an antibody recognizing the extracellular domains of both APP full-length and APP-CTFβ (Appendix Fig. S9, AB#2, clone: M3.2). However, incubation with an antibody targeting specifically the ectodomain of full-length APP protein had no effect (Appendix Fig. S9, AB#3). These observations indicate that APP-CTFβ species are associated with rapid, surface-accessible modulation of presynaptic function.

These findings further warrant in-depth investigation of APP C-terminal-dependent calcium signaling events *via* Syt1 and/or GTP-binding protein G(o) (Nishimoto et al, 1993; Hanley and Selkoe, 1993), which modulate release probability. In our simulations, we uncovered a preferential interaction between Arg76 of APP-CTFβ and the PIP2 lipid head group. This interaction influences the positioning of the C-terminal domain of APP-CTFβ in close proximity to the plasma membrane, preventing its binding to Syt1. Accumulation of APP-CTFβ facilitates the formation of dimers or trimers, which in turn decrease its interaction with the PIP2 head group, favoring its interaction with synaptic vesicle proteins. Thus, APP-CTFβ interaction with the synaptic vesicles and its oligomerization contribute to neurotransmitter release. Our data is supported by independent findings reported in several studies highlighting that BACE1 activity is essential for optimal release of synaptic vesicles, and its deficiency or pharmacological inhibition decreases the number of docked synaptic vesicles at the active zones (Das et al, 2021; Das and Yan, 2017; Yan et al, 2016; Wang et al, 2008).

In the context of early synaptic deficits observed in AD, altered APP processing due to either increased β-secretase activity or γ-secretase deficits could result in APP-CTFβ accumulation, contributing to the alterations in synaptic vesicle fusion dynamics; in addition to the more widely recognized toxicity associated with Aβ species. Supporting our findings, both APP and PSEN1 FAD mutations that result in loss of function of γ-secretase (i.e., stalling of PSEN-APP-CTFβ complexes and/or membrane retention of longer forms of Aβ) show augmented synaptopathy and neurodegeneration phenotypes, associated with early synaptic dysfunction in AD (Essayan-Perez and Südhof, 2023; Tamayev et al, 2012). Multiple reports have shown that during aging (a risk factor implicated in AD), amyloidogenic APP proteolysis is augmented *via* an increase in β-secretase activity, which increases APP-CTFβ accumulation within neuronal sub-compartments, associated with altered neuronal activity (Burrinha et al, 2021; Fukumoto et al, 2004; Rajão-Saraiva et al, 2023). In addition, presynaptic accumulation of APP-CTFs is associated with synaptic dysfunction *via* APP-dependent reduction of the calcium sensor Synaptotagmin-7, leading to impaired vesicle replenishment (Barthet et al, 2018). Recent evidence has shown that APP-CTFβ alone, in the absence of Aβ, can induce neuronal dyshomeostasis (Tian et al, 2014; Sergeant et al, 2002; Lauritzen et al, 2019b; Lee et al, 2020, 2022; Luo et al, 2025; Bretou et al, 2024; Tamayev et al, 2012; Harwell and Coleman, 2016), suggesting these effects are independent of extracellular Aβ peptides. In addition, reports have also shown that APP and its processing enzymes along with synaptic markers are detected around plaque periphery in both human AD patient samples and transgenic mouse models (Jordà-Siquier et al, 2022). Taken together, we propose that APP-CTFβ in itself or alongside Aβ, contribute to early synaptic disruptions, an understudied avenue in the pathophysiology of AD.

Our findings highlight the contributions of presynaptic APP-CTFβ in regulating synaptic activity and its association to synaptic alterations. Now, acute treatment of Aβ (5 min), particularly the 42 variant, attenuates GSI-induced network hyperactivity *via* postsynaptic mechanisms. While others have reported synaptogenic properties of Aβ (or APP amyloidogenic proteolytic products) (Zhou et al, 2022; Schilling et al, 2023); literature evidence supports the fact that there are beneficial effects of lower concentrations of Aβ in maintaining neuron/synapse health (Wang et al, 2012; Parihar and Brewer, 2010; Schilling et al, 2023; Kent et al, 2020). The functional transition of Aβ from physiological to pathological states is concentration dependent (Siddu et al, 2025; Cavallucci et al, 2012), wherein subtle changes in Aβ levels potentially tip the balance between normal synaptic function and neurodegeneration (Bilousova et al, 2016; Kobro-Flatmoen et al, 2016; Eckman et al, 2023; Takahashi et al, 2004). These results underscore the necessity to expand on the Aβ-centric perspective to encompass the broader spectrum of APP processing products, which may have substantial implications for both synaptic physiology and disease progression (García-Ayllón et al, 2017; Gaetani et al, 2025; Oh et al, 2025). To further substantiate this, we also remark that the balance of α- or β-secretase-dependent processing of APP can either offer protection (e.g., Icelandic mutation) or drive the disease pathogenesis (e.g., Swedish mutation), suggesting that alterations in APP metabolism, in addition to Aβ changes, may contribute to AD onset (Jónsson et al, 2012; Thordardottir et al, 2017; Vargas Baron, 2024; Annaert et al, 2000; Tambini et al, 2020). Indeed, supporting our findings in postmortem AD cases, others have shown evidence of APP-CTFβ accumulation with synaptic structures/neurons in both transgenic mouse models (Lauritzen et al, 2019b, 2019a, 2016) as well as human AD patients (Daly et al, 1998; Ferrer-Raventós et al, 2023; Vaillant-Beuchot et al, 2021). While our study provides strong evidence for the role of amyloidogenic products in synaptic dysfunction, particularly APP-CTFβ-induced defects at the presynapse; several limitations must be acknowledged. Our in vitro experiments rely on rodent/murine models, which may not fully capture the complexity of human systems. Interpretations based on computational modeling and correlation with experimental approaches do not establish direct causal mechanisms. Analysis of human AD brain samples was limited by cohort size and the availability of material.

Taken together, our results and existing literature suggest that both Aβ and APP-CTFs/β contribute to synaptic dysfunction in a context- and stage-dependent manner. While Aβ-targeted antibody treatments have been shown to reduce amyloid burden and provide modest clinical benefits, their effect on synaptic function remains less clear (Jin et al, 2024; Noorda et al, 2024; Schreiner et al, 2024; Jicha, 2009; Sang, 2025; Tonegawa-Kuji et al, 2025; Panza et al, 2025; Nitrini and Studart-Neto, 2025). These observations suggest that modulation of Aβ alone may not be sufficient to fully address synaptic and neuronal defects. Effective therapies may benefit from considering the physiological role of APP processing and its diverse components. Importantly, a more holistic and comprehensive framework that considers the role of both Aβ and APP-CTFβ at the distinct stages of AD would lead to treatments that preserve and restore synaptic function.

## Methods

**Table:** Reagent and tools table

Reagent/resource	Reference or source	Identifier or catalog number
**Experimental models**		
vGLUT1^GFP^ and wild-type mice	Generated in-house *via* CRISPR/Cas-mediated genome editing (outsourced to Cyagen)	Details provided in the appendix
Rat primary neuronal cultures	Janvier	
**Recombinant DNA**		
Synapto-pHluorin	Addgene plasmid #168510	https://www.addgene.org/168510/
AAV.Camk2-GcAMP6	Generated in-house, serotype AAV1/2	
**Antibodies**		
Rabbit anti-APP N-terminal specific	Sigma-Aldrich #SAB4200536	RRID:AB_2818976
Mouse anti-APP- Aβ mid region specific (M 3.2)	Covance #SIG-39155	RRID:AB_2531964
Mouse anti-APP C-terminal fragment specific (C1/6.1)	Covance #SIG-39152	RRID:AB_2531961
Rabbit anti-APP C-terminal fragment specific (3125)	In-house generated (JW) (Eurogentec)	
Rabbit anti-APP C-terminal fragment specific (140-rb)	In-house generated (JW) (Eurogentec)	
Rabbit anti-soluble APPα	Covance #SIG-39139	RRID:AB_2531957
Rabbit anti-soluble APPβ	Covance #SIG-39138	RRID:AB_2531956
Mouse anti-Aβ_1-x_ (82E1)	IBL Int. #JP10323	RRID:AB_2715500
Mouse 82E1-Biotin conjugated	IBL Int. #JP10326	RRID:AB_2715501
Rabbit anti-ratAβ	Abcam # ab14220	RRID:AB_301636
Rabbit anti-Aβ_x-40_ (BAP-29)^‡^	Brockhaus et al, 1998^‡^	
Rabbit anti-Aβ_x-42_ (BAP-15)^‡^	Brockhaus et al, 1998^‡^	
Rabbit anti-hAβ (2964)	Generated by Walter lab (Eurogentec)	
Guinea pig anti-Vesicular Glutamate Transporter (vGLUT1)	SYSY #135 304	RRID:AB_887875
Guinea pig anti-Vesicular GABAergic Transporter (vGAT)	SYSY #131 002	RRID:AB_887873
Rabbit anti-vGAT Cytoplasmic tail (rb)	SYSY #131 004	RRID:AB_887874
Guinea Pig anti-Synapsin1/2	SYSY #106 004	RRID:AB_2619753
Mouse anti- Synapsin	SYSY #106 011	RRID:AB_2619756
Rabbit anti-PSD95	CST #3450	RRID:AB_2292883
Mouse anti-PSD95	Abcam #ab2723	RRID:AB_303248
Mouse anti-PSD95	DSHB #k28/43	RRID:AB_528100
Chichen anti-Green fluorescent protein (GFP)	Abcam #ab13970	
Rabbit anti-α-secretase (ADAM10)	TFS #MA5-32616	RRID:AB_2801652
Rabbit anti-β-secretase (BACE1)	CST #5606S	RRID:AB_10827940
Rabbit anti-Presenilin-1 (D39D1)	CST #5643	RRID:AB_10707366
Rabbit anti-Presenilin-1 (NTF 3110)	In-house generated (JW) Eurogentec	
Rabbit anti-Nicastrin (D38F9)	CST #5665	RRID:AB_10828675
Mouse anti-PtdIns(4,5)P2 (2C11)	Echelon Biosciences #Z-P045	
Rabbit anti-Histone/Chromatin (Histone H3)	Abcam #ab1791	
Rat anti-Regulating synaptic membrane exocytosis protein 1 (RIM1/2)	SYSY #140 217	RRID:AB_2619840
Guinea pig anti-Syntaxin-1 (STX1)	ProteinTech #83159-6-RR	RRID:AB_2915710
Guinea pig anti-Synaptosome Associated Protein 25 (SNAP25)	SYSY #111 004	RRID:AB_887807
Rabbit anti-Synaptotagmin-1 (Syt1)	SYSY #105 008	RRID:AB_887831
Mouse anti-Syt1	SYSY #105 011	RRID:AB_887833
Mouse anti-Syt1	DSHB #mAB30- asv30	RRID:AB_2313996
Rabbit anti-Synaptobrevins (Syb)	SYSY #104 002	RRID:AB_887810
Guinea pig anti-Vesicle-associated membrane protein- 1/2/3 (VAMPs-1/2/3)	SYSY #104 102	RRID:AB_887812
Guinea pig anti-Presynaptic cytomatrix protein Bassoon (Bsn)	SYSY #141 318	RRID:AB_887738
Guinea pig anti-Postsynaptic density-localized scaffolding protein Homer1	SYSY #160 004	RRID:AB_2619870
Mouse anti-Recombinant Puromycin (3RH11)	Kerafast #Kf-Ab02366-1.1	RRID:AB_2650491
Mouse anti-Flotillin (FLOT1)	BDB #610820	RRID:AB_398139
Rat anti-Transferrin Receptor (YTA 74.4)	Abcam #ab22391	RRID:AB_445484
Mouse anti-Transferrin Receptor	IHB #G1-221-12	RRID:AB_2619735
Mouse anti-GAPDH	SCB #sc-32233	RRID:AB_627679
Mouse anti-GAPDH	HelloBio #HB9177	RRID:AB_2651167
Guinea Pig anti-Microtubule protein	SYSY #188 004	RRID:AB_2619984
Chicken anti-Microtubule protein	SYSY #188 006	RRID:AB_2619985
Guinea Pig anti-Beta-actin	SYSY #251 005	RRID:AB_2619988
Goat anti-chicken IgG 405	Abcam #ab175674	
Goat Anti-Chicken IgY H&L A488	Abcam #ab150169	
Goat anti-Mouse IgG A405	Ozyme #BTM20080-1MG	
Goat anti-Mouse IgG A488	Abcam #ab150113	
Goat anti-Mouse IgM (Heavy chain) A568	Invitrogen #A21043	
Goat anti-Mouse IgG A647	Abcam #ab150115	
Goat anti-Mouse IgG Atto647	Sigma-Aldrich #50185-1ML-F	
Goat anti-Rabbit IgG A488	Abcam #ab150077	
Goat anti-Rabbit IgG A568	Abcam #ab175471	
Goat anti-Rabbit IgG A568	Ozyme #BTM20102-1MG	
Goat anti-Guinea Pig IgG A488	Abcam #ab150185	
Goat anti-Guinea Pig IgG A647	Abcam #ab150187	
Streptavidin Alexa 568 Conjugate	TFS #S11226	
Streptavidin, Alexa Fluor™ 647 conjugate	Biolegend #405237	
Goat anti-Mouse IgG (H + L) Secondary Antibody, HRP	TFS #32430	
Goat anti-Rabbit IgG (H + L) Secondary Antibody, HRP	TFS #31460	
Goat anti-Guinea Pig IgG (H + L) Secondary Antibody, HRP	TFS #A18769	
Streptavidin HRP	Biolegend #1474; TFS #N100	
**Oligonucleotides and other sequence-based reagents**		
siRNA targeting APP	ThermoFischer Scientific, #4390815	siRNA ID s132407
**Chemicals, enzymes, and other reagents**		
4′,6-diamidino-2-phenylindole (DAPI)	TFS #D1306	
EGTA-free protease inhibitor cocktail	Calbiochem, # 539134-1 ML	
HEPES	Sigma-Aldrich, #H4034-100G	
Sucrose	Merck, #S9378-1KG	
Triton X-100	Merck, #T8787	
BSA	Carl Roth, #8076-4	
Moviol 4-88	CarlRoth# 0713.1	
SYNPER synaptic protein extraction reagent	ThermoFischer Scientific #87793	
Nunc® MaxiSorp™ 384 well plates	Sigma-Aldrich, #P6366	
3,3′,5,5′-tetramethylbenzidine substrate	TMB; ThermoFischer Scientific, #34029	
ELAST ELISA Amplification System	Revvity, #NEP116001EA	
LY2886721 (β-secretase inhibitor, BSI)	Cayman chemicals, #21599-1	
DAPT (γ-secretase inhibitor, GSI)	Cayman chemicals, #13197-5	
Aftin-4 (activator of amyloidogenic processing; APA)	Cayman chemicals, #27341-1	
GI25423X (α-secretase modulator, ASM)	Cayman Chemicals #28284-1 mg	
Compound E (γ-secretase inhibitor, GSI2)	Cayman Chemicals #15579-500 µg	
FLI-06 (inhibitor of the notch signaling pathway, NSI)	Cayman Chemicals #21272-5 mg	
CNQX (blocking post-synaptic AMPA receptors)	Sigma-Aldrich #C127-5MG	
AP5 (post synaptic NMDA receptor antagonist)	HelloBio #HB0225-10mg	
Anisomycin (protein synthesis inhibitor)	Tocris #1290	
SynaptoRed C2	Sigma-Aldrich, #S6689-5MG	
Pierce™ BCA protein assay kit	ThermoFischer Scientific, #23225	
4–15% or 4-20% NuPage^Ⓡ^ Bis-Tris gels	Bio-Rad, #4561083EDU, 4561096EDU	
0.2-μm nitrocellulose (NT) membrane	Amersham, #1060001	
Skimmed milk powder	Cal Roth, #T145-1	
Tween 20	ThermoFischer Scientific, #J20605.AP	
Puromycin	Tocris #4089	
FAM-puromycin	Jena Bioscience #NU-925-6FM-S	
Synthetic Aβ peptides	PSL peptide laboratories	
Trichloroacetic acid (TCA)	Thermo Fisher Scientific #A11156.30	
EM grade 4% PFA	Electron Microscopy Sciences #157-4	
Glass-bottom dishes	Mattek, #P35G-1.5-14-C-HA	
Ultrasmall-gold secondary antibody	Aurion, #800.022/11	
Glutaraldehyde	Carl Roth #4157.1	
NaBH4	Sigma-Aldrich, #213462-25 G	
Glycine	Merck, #GE17-1323-01	
Ammonium chloride	Sigma-Aldrich, #A4514-100G	
ECS (Enhancement Conditioning Solution)	Aurion, #500.055	
Epon epoxy resin	Sigma-Aldrich, #45359-1EA-F	
Copper-coated copper slot grid	Science Services, #EFCF2010-Cu-50	
Lead citrate Ultrastain solution	Leica, #16707235	
Magnetic Sepharose A/G beads	Sigma-Aldrich, #GE28-9440-06	
Antigen retrieval solution	Reveal Decloaker #SKU: RV1000M	
**Software**		
GraphPad Prism	10.4.1.627	RRID:SCR_002798
Fiji ImageJ		RRID:SCR_002285
Imaris version 9	Bitplane, Oxford Instruments	RRID:SCR_007370
Image Studio processing software (LiCOR Biosciences, Germany).		RRID:SCR_014211
**Other**		
Cytek® AuroraCS equipped with a 70-µm nozzle	Cytek Biosciences, Netherlands	
Nikon live-cell SoRa spinning disk super-resolution microscope	Nikon Instruments, Japan	
Leica SP8 Liachroic confocal microscope	Leica Microsystems, Germany	
Leica Widefield microscope equipped with a Thunder computational clearing module	Leica Microsystems, Germany	
ECL imager	Bio-Rad, Germany	
LiCOR Western blot Imager	LiCOR Biosciences, Germany	
Zeiss Crossbeam 550	Carl Zeiss AG, Germany	

*BDB* BD Biosciences, *CST* Cell signaling technology, *IHB* Iowa Hybridoma bank, *SYSY* Synaptic systems, *SCB* Santa Cruz Biotechnology, *TFS* Thermo Fisher Scientific.

^‡^Gift from from Dr. M. Brockhaus (Roche). In-house-generated antibodies or received from collaborators do not have published RRIDs. RRIDs were included where available according to SciCrunch (https://scicrunch.org/resources). Details about the concentrations used in each experiment have been indicated in Appendix Table S1.

### Synaptosome isolation

Mice were handled and maintained according to the guidelines laid down by the Animal Welfare Body (AWB) (Instantie voor Dierenwelzijn IvD) in Mouse Eurostandard type II L cages in groups in line with the animal experimentation policy within Radboud University and RadboudUMC; under the license AVD10300 2021 15635 and protocol 2021-0040-001 to Dr. Anne-Sophie Hafner. vGLUT1^GFP^ knock-in mice (C57BL/6N) were generated *via* CRISPR/Cas-mediated genome editing (outsourced to Cyagen), and detailed information including targeting strategy, gene/transcript data, and expression analysis is provided in the Appendix Figs. S17–S20. Synaptosomes were enriched from the forebrains of 1-year-old wild-type and vGLUT1^GFP^ knock-in mice. To favor the isolation of synaptosomes with presynaptic compartments with closed membrane bilayers and postsynaptic compartments with open membrane bilayers (Whittaker et al, 1964), we used the adapted protocol, previously described ((Luquet et al, 2017; Hafner et al, 2019 and Appendix Table S3). Forebrain from a single mouse was homogenized in 2 ml of ice-cold homogenization buffer 0.32 M sucrose (Merck, #S9378-1KG), 4 mM HEPES (Sigma-Aldrich, #H4034-100G; pH adjusted to 7.4), EGTA-free protease inhibitor cocktail (Calbiochem, # 539134-1 ML; 1:1000), by using a 2-ml glass-Teflon homogenizer with 15-17 gentle strokes. An additional 2 ml of buffer was used for rinsing, and the combined 4 ml was centrifuged at 1000×*g* for 8 min at 4 °C. The supernatant (S1) was centrifuged at 12,500×*g* for 15 min at 4 °C. The synaptoneurosome-enriched pellet (P2) was then resuspended in 1 ml of homogenization buffer. This fraction was finally layered on top of a two-step sucrose density gradient (5 ml of 1.2 M sucrose and 5 ml of 0.8 M sucrose, 4 mM HEPES, and EGTA-free protease inhibitor cocktail, as described above). The gradient was ultracentrifuged at 40,000×*g* for 70 min at 4 °C. Synaptosome fractions were recovered through the tube wall, at the interface of 0.8 and 1.2 M sucrose, by using a syringe to minimize contamination with lighter fractions enriched in myelin. The resulting fraction is referred to as presorted synaptosomes (or presort. SYN).

### Fluorescence-activated synaptosome sorting (FASS)

Synaptosome sorting was performed as previously described (Luquet et al, 2017; Hafner et al, 2019). The Cytek® AuroraCS was operated using a 70-µm nozzle. Briefly, presort. SYN were stored on ice, diluted in PBS containing protease and RNAase inhibitor as described above. Dilution was optimized to obtain an event rate of 15,000–20,000 events/s with an optimal flow rate to maintain a single particle per droplet. A threshold was set on SSC-A to discriminate against the noise. A dilution range was made to confirm the correct settings. A first gate delineated small particles (“singlets”) and excluded events showing correlated high values for forward scatter and side scatter areas (aggregates and large particles). The singlets gate was sub-gated according to vGLUT1GFP fluorescence intensity by using the 488-laser line. Thus, singlets were sorted into two fractions, the vGLUT1^GFP^-negative (GFP −) fraction and the vGLUT1^GFP^-positive (GFP + ) fraction (Fig. EV1A, *scheme*). These two fractions were either collected onto filters and processed for homogenate preparation or plated onto gelatinized coverslips at a density of 1 Mio particles per 12-mm coverslip in 24-well plates by centrifugation at 5000×*g* for 30 min and then processed for ICC as described ahead.

### Neuron cultures

Pregnant rats RjHan:SD from Janvier were handled and maintained according to the guidelines laid down by the Animal Welfare Body (AWB) (Instantie voor Dierenwelzijn IvD) in Rat Eurostandard type IV S cages in isolation in line with the animal experimentation policy within Radboud University and RadboudUMC; under the licenses AVD10300 2021 15635 and AVD10300 2024 17994 and on the respective protocols 2021-0040-002 and 2024-0009-003 to Dr. Anne-Sophie Hafner. The pregnant rats were housed at the animal house CDL (RadboudUMC) for 5 days before being sacrificed. Briefly, cortices were dissected from embryonic (day 18) rat pups of either sex (Sprague-Dawley strain; Janvier), trypsinised (1×, 15–20 min), mechanically dissociated in 1% normal horse serum (NHS; ThermoFischer Scientific, #16050130) containing Basal Media Eagle (Life technologies, #21010046), containing supplements like B27 plus (Gibco, #A3582801), penicillin-streptomycin (1× PS, Gibco, #15140122), non-essential amino acids (1× NEAA, ThermoFischer, #11140050), sodium pyruvate (1×, NaPy, ThermoFischer, #11360070). Dissociated cells were plated on poly-D-lysine-coated (Sigma-Aldrich, #A-003-E) glass coverslips (VWR, #631-1577/79/84) or 35 mm glass-bottom dishes (Mattek, #P35G-1.5-14-C-HA) at a specific density to achieve optimal neuron network connections. Neurons were maintained and matured in a humidified atmosphere at 37 °C and 5% CO_2_ in growth medium (described above, without serum). Neurons were fed by replacing 1:1 media replacement with BrainPhys™ neuronal medium (StemCell technologies, # 05790) supplemented with NeuroCult™ SM1 neuronal supplement (StemCell technologies, # 05711) and PS (1×) starting DIV4 until DIV21-22 to ensure synapse maturation. All experiments complied with national animal care guidelines and the guidelines issued by the Radboud UMC, Nijmegen, and were approved by local authorities (mentioned earlier). For transduction, 3–5-DIV neurons were transduced with AAV-Camk2-GCaMP6s, and transfected cells were maintained until 18 to 21 DIV for further experiments.

### Pharmacological treatments

Primary cortical neurons (DIV21-22) were treated with LY2886721 (1 μM; BSI; Cayman chemicals, #21599-1), DAPT (10 μM; GSI; Cayman chemicals, #13197-5), or Aftin-4 (5 μM; APA; Cayman chemicals, #27341-1) for 4 h in culture media, to manipulate different stages of APP proteolysis. Post-treatment neurons were either fixed with 4% PFA + 4% sucrose in PBS for 15 min at RT for ICC or subjected to synaptoneurosomes isolation or lysate/ homogenate preparation, respectively. Treatment with Aβ peptides (100 nM, 5 min) was done following with or without prior GSI treatment and excess peptides were washed with PBS (thrice) followed by live imaging or fixation for ICC.

### Immunocytochemistry

Neurons after fixation were permeabilized with permeabilization buffer (0.025% Triton X-100 (Merck, #T8787-250ML) in blocking buffer) for 2 min, followed by blocking (2.5% NHS, 2.5% bovine serum albumin (BSA; Carl Roth, #8076-4), 0.0125% Triton X-100 in 1× PBS) for 1 h at RT. An antigen retrieval step was performed to augment and facilitate binding of APP-CT/ APP antibodies. Incubation with primary antibodies (respective dilutions in blocking buffer, Appendix Tables S1 and 2) was performed overnight at 4 °C. The next day, coverslips were washed thrice with PBS and incubated with respective secondary antibodies (respective dilutions in blocking buffer, Appendix Table S1) for 2 h at RT. Removal of the secondary antibody solution was followed by washes of PBS (thrice) and distilled water (once), and coverslips were mounted on the slides using Moviol mounting reagent (41.67% Glycerol, 16.67% Moviol 4-88 (CarlRoth# 0713.1) in ddH₂O).

### ELISA

Equal amounts of protein from various brain, synaptosome, or cellular fractions (based on antibody LOD quantifications, Appendix Table S4) were added to Nunc® MaxiSorp™ 384 well plates (Sigma-Aldrich, #P6366) and incubated overnight at 4 °C. Plates were washed, blocked with BSA, and incubated with primary antibodies (dilutions mentioned in Appendix Table S1) at room temperature or 4 °C. After washing, plates were incubated with respective HRP-conjugated secondary antibodies, followed by TMB substrate development (ThermoFischer, #34029). The reaction was stopped with 4M sulfuric acid, and the absorbance was measured at 450 nm with background correction at 620 nm. Samples were analyzed in technical duplicates or triplicates. Detailed experimental protocols for sandwich or dual immunoisolation ELISA, and signal amplification using a tyramide-based system, please refer to the Appendix file.

### Western blotting

Protein concentration of all prepared samples was estimated using the standard Pierce™ BCA protein assay kit (ThermoFisher Scientific, 23225). An equal amount of protein (10–20 μg) was loaded per lane, except Fig. 1B (S1, P2, presort. SYN = 20 μg, Sort. SYN = 1 μg). Briefly, proteins were separated on a precast 4–15% or 4–20% NuPage® Bis-Tris gel (Bio-Rad, #4561083EDU, 4561096EDU). Human brain lysates (20 μg) were loaded on a 4–12% NuPage® Bis-Tris gels (Life Technologies, NP033-5/6, WG140-2/3). Separated proteins were electro-transferred onto a 0.2-μm nitrocellulose (NT) membrane (Amersham, #1060001) for 10 min using the quick transfer protocol (Trans-Blot turbo transfer system, Bio-Rad) or for 1 h 45 min using the wet-transfer method. For detection of APP-FL, APP-CTFs/β or Aβ; samples were boiled for 5 min at 95 °C. In addition, NC membrane blots post-transfer were placed in boiling PBS solution. Equal loading of protein concentration was determined by staining the separated proteins using Ponceau S staining solution. The blots were then incubated in 5% skim milk (Cal Roth, #T145-1) in TBS-T containing 0.1% Tween 20 (ThermoFischer Scientific, #J20605.AP) for 1 h at RT. Blots were incubated with primary antibodies (overnight at 4 °C); followed by respective secondary antibodies (2 h at RT). Antibody dilutions have been summarized in Appendix Table S1. Proteins were detected either by ECL imager (Bio-Rad, Germany) or fluorescent imager (LiCOR Biosciences, Germany). Detailed descriptions have been provided in the SI. Values from each sample were normalized to the values from the control cells in that experiment, and fold change with respect to the control was indicated as a graph.

### Aβ treatment

Untreated or γ-secretase inhibitor (GSI) pre-treated neurons were treated with or without synthetic Aβ peptides (100 nM) for 5 min at 37 °C. Following incubation, cells were washed three times with pre-warmed culture medium to get rid of excess Aβ and used for live imaging experiments as described earlier. Preparation of monomeric Aβ peptides is described in the Appendix, along with other experimental protocols.

### Calcium and SynaptoRed imaging

Neurons expressing Camk2-GCaMP6 (AAV transduction, DIV3) were subjected to treatment with different modulators (DIV21, as indicated previously). Spontaneous calcium transients were recorded in the absence and presence of Ca^2+^ in Tyrode buffer for 5 min continuously in 500 ms intervals. ROIs were either derived from the complete neuron (Fig. 2I,J) or segmented to differentiate between soma and dendrite (Appendix Fig. S2A,B), and Ca^2+^ traces per minute have been depicted to show the number of spontaneously evoked Ca^2+^ transients per minute. Dual imaging of neuronal Ca^2+^ response and synaptic vesicle release events by SynaptoRed C2 (Sigma-Aldrich, #S6689-5MG, 16 nM in culture media), wherein synaptic vesicles were loaded with the dye *via* stimulation at 35 Hz, excess dye was washed off with fresh media thrice, and neurons were rested for 5 min to ensure stable baseline recordings. This was quantified in a pilot experiment, and thus the same timeline was maintained for the entirety of the experiments henceforth. Calcium and synaptic vesicle dynamics were imaged and recorded during the stimulation protocol with 30 Hz, 4 repetitions, starting 7th s, with 30 s recovery period. Data was recorded for 2 min at every 125 ms intervals. Time-lapse images were then processed to either quantify the total Ca^2+^ response (Fig. 3C) or just the dendrite (ROIs, Fig. 3B; Appendix Fig. S4A,B). Fluorescence intensities of SR loaded within synaptic vesicles were then quantified for the specific dendritic ROI (ROIs, Fig. 3F; Appendix Fig. S4C,D) or SR-positive ROIs from the complete frame (Fig. 3G,H). Detailed analysis has been explained in Movie EV2, and representative data has been depicted in Movies EV2–4. In an additional SynaptoRed-only experimental paradigm, synaptic vesicle dynamics were imaged in control, GSI, and GSI + Aβ conditions (4 h treatment) in the presence or absence of TTX (500 nM), following the same stimulation-loading and washout timeline as above (Fig. 3I, *scheme*).

### pHluorin imaging

Neuronal cultures were transfected with Synaptophysin-pHluorin *via* a lipofectamine-based transfection protocol at DIV7. At DIV 21, neurons were treated for 2 h with either GSI, and GSI + Aβ or left untreated in the absence or presence of TTX (1 µM final) applied simultaneously and washed off before imaging for experiments (Fig. 3M, *scheme*). Imaging began at least 10 s before stimulation to establish baseline fluorescence and was acquired continuously at 125 ms frame intervals. Field stimulation protocol was the same as for SynaptoRed experiments described earlier: 30 Hz trains, 4 repetitions, first train starting at 7 s, and 30 s inter-train recovery periods. Total imaging length was ~2–2.5 min covering baseline, stimulation trains, and immediate post-stimulation periods. To correct for expression level and report release as a fraction of the total releasable pool, after live imaging, neurons were perfused with NH₄Cl solution (50 mM NH₄Cl replacing an equimolar amount of NaCl in Tyrode) for 5 min to alkalinize acidic compartments and reveal total pHluorin fluorescence (F_NH₄Cl). Lastly, neurons were fixed and processed for immunocytochemistry.

### Electron microscopy

Neurons cultured on glass-bottom dishes (Mattek, #P35G-1.5-14-C-HA) were live-labeled with or without an antibody targeting the extracellular N-terminal domain of APP-CTFβ (AB#1) or full-length APP (AB#2/3), then washed and fixed with PFA (Electron Microscopy Sciences #157-4). After blocking, cells were incubated with a secondary gold-conjugated antibody (1:50; Aurion, #800.022/11), followed by a second fixation with glutaraldehyde (Carl Roth #4157.1). Excess reagents were washed off, and samples were quenched using sodium borohydride and glycine/ammonium chloride. After additional washes, silver enhancement (Aurion, #500.044) was performed. Cells were then osmicated, stained with uranyl acetate, and dehydrated through graded ethanol. Resin infiltration and polymerization followed. Blocks were detached from dishes, trimmed, and ultrathin-sectioned. Sections were post-stained with uranyl acetate and lead citrate, then imaged using STEM mode on a Zeiss Crossbeam 550. Multiple regions per grid were imaged to ensure unbiased representation. Detailed experimental protocol and additional information have been summarized in the Appendix. Images were minimally processed using Fiji/ImageJ.

### Synaptosome lipid and protein fractionation

Synaptosome fraction (Appendix Table S3, adult rat brains or cultured primary neurons) was homogenized in SynPer reagent (without any surfactant) by multiple freeze–thaw cycles to maintain protein-protein interaction complexes. Samples were then loaded on a density gradient (rat brain, 5%–2.5 ml, 35% - 6 ml and 42.5% 1.5 ml; cultured neurons, 5%–1 ml, 35%–3 ml, 42.5% 1 ml and 60%–6 ml sucrose in HEPES buffer) ultracentrifugation (288,000× *g*, 12 h, 4 °C) to fractionate synaptic membranes into cholesterol and PI(4,5)P2 enriched and the perisynaptic membrane fraction. The next day, 10–1 ml or 14–0.3 ml fractions were collected, and the material was subjected to ELISA analysis to examine the levels of different proteins and PI(4,5)P2 lipids. Cholesterol in each fraction was analyzed using Amplex™ Red Cholesterol Assay Kit (ThermoFischer Scientific, #A12216) as per the manufacturer’s protocol without modifications.

### Immunoisolation

Synaptosomes (Appendix Table S3, rat brains) were homogenized in SYNPER protein extraction reagent containing protease and phosphatase inhibitors by repeated suspension through a 23 G × 1” needle. Synaptosome homogenate was then used for IP experiments using magnetic Sepharose A/G beads (Sigma-Aldrich, #GE28-9440-06/). Beads were first coated with respective antibodies for 1 h at RT (Appendix Table S1), washed 3× in PBS, and then incubated with material at 4 °C for 16 h. Beads were further pelleted down and boiled in 1% SDS + 50 mM Tris + sample buffer, used as IP eluate, and the remaining supernatant was loaded as sup. Beads incubated only with the homogenate were used as the bead control. The supernatants exposed to beads only were devoid of non-specific binding substrates and were used further as a loading control. Similarly, a dual immunoisolation protocol, primarily with APP-N-terminal ectodomain antibody to deplete FL-APP, and secondly with an APP antibody (clone: M3.2) to enrich the APP-CTFβ pool. Protein–protein interactions to distinguish relative pools of synaptic proteins interacting with full-length APP and APP-CTFβ were done using ELISA.

### Computational studies

The protein structure was obtained from an earlier study by Pantelopulos et al (Pantelopulos et al, 2022, 2018), made available through the author’s GitHub (GitHub - gpantel/MD_methods-and-analysis: Repository for MD methods and analysis from submitted or published work). The prepared initial structure (modifications explained in Appendix, initial structure and coarse-graining and Appendix Figs. S10 and S11) was used to prepare three different simulation setups (All-atom with the CHARMM force field (Huang et al, 2017): realistic and POPC membrane with a single APP-CTFβ (runs #1-3), total- 6 simulations; Coarse-grained with the Martini 3 force field (Souza et al, 2021; Pedersen et al, 2024): realistic and POPC membrane with 1×, 2×, and 3× APP-CTFβ each, in two models each, (runs #1-3), total: 36 simulations). An all-atom model and two coarse-grained simulations that differ in their choice of modeling the secondary structure of the proteins. Detailed information on modeling parameters, analysis, and calculations, etc., is described in detail in the Appendix.

### Immunolabelling of human brain sections

Ethical approval was obtained either prior to death from donors or their next of kin signed informed consent for brain autopsy and use of brain tissue and medical records for research purposes. The brain donor program of the Netherlands Brain Bank (NBB) was approved by the local medical ethics committee of the VUmc with ethical approval reference number 2009/148. Formalin-fixed paraffin-embedded postmortem human brain sections (obtained from the Netherlands brain bank-NBB, demographic information in Appendix Table S7) were deparaffinized and rehydrated through a graded xylene-alcohol series (xylene ×3, 100% ethanol ×2, 90% ethanol ×1, 70% ethanol ×2) and rinsed twice in PBS. Heat-assisted antigen retrieval was performed by incubating sections in antigen retrieval solution (Reveal Decloaker #SKU: RV1000M) and placing them in a water bath at 95 °C for ~1 h, followed by cooling to room temperature in PBS for 15-20 min. Sections were washed in PBS (3 × 5 min) and incubated with blocking solution for 2 h at RT. Incubation with primary antibodies diluted in blocking buffer (respective dilutions, Appendix Tables S1 and 2) was performed overnight at 4 °C. The following day, slides were washed in PBS (3 × 5 min) and incubated with respective secondary antibody solutions for 2 h at RT. Removal of the secondary antibody solution was followed by washes in PBS (3 × 5 min) and distilled water (once). Finally, sections were counterstained with DAPI and mounted under glass coverslips using Moviol mounting reagent. Mounting media was allowed to harden overnight at RT.

### Microscopy and image processing

Confocal microscopy images were acquired on Nikon live-cell SoRa spinning disk super-resolution microscope (Nikon technologies, Japan). or Leica SP8 Liachroic confocal microscope (Germany) or Leica Widefield fluorescence microscope equipped with Thunder computational clearing module (Germany). Imaging parameters have been mentioned in detail in the Appendix file.

### Statistical analyses

All graphical illustrations have been prepared using GraphPad Prism 10.4.1.627. Statistical analysis for a particular graph has been mentioned in the respective figure legends. Biological and technical replicates have been consistently defined throughout the manuscript. Capital ‘*N*’ refers to independent biological replicates, i.e., independent neuronal preparations prepared in different batches. Lowercase ‘*n*’ refers to technical replicates or individual measurements within the particular biological replicate. All parametric and nonparametric tests have been performed using the in-built default settings of the module in the software, without modifications, until mentioned otherwise; not significant (ns) *P* > 0.05; *P ≤ 0.05; **P ≤ 0.01; ***P ≤ 0.001. When possible, we blinded the data during the analysis.

## Supplementary information

Appendix

Peer Review File

Movie EV1

Movie EV2

Movie EV3

Movie EV4

Movie EV5

Movie EV6

Source data Fig. 1

Source data Fig. 2

Source data Fig. 3

Source data Fig. 4

Source data Fig. 5

Source data Fig. 6

Source data Fig. 7

Figures EV1-4 Source Data

Expanded View Figures

## Data Availability

All experimental data from the main text and the supplementary materials as well as computational simulation code and materials used in the analysis are available on the data sharing server Donders Repository (https://doi.org/10.34973/7cwk-sx42). The source data of this paper are collected in the following database record: biostudies:S-SCDT-10_1038-S44318-026-00899-6.
