# Supplementary material for: Presynaptic accumulation of APP-CTFβ may contribute to synaptic dysfunction in Alzheimer’s disease
Source: EMBO J. 2026 Sep 1;45(19):6914–45. doi: 10.1038/s44318-026-00899-6 (PMC13631370; doi:10.1038/s44318-026-00899-6)
Supplement: Supplementary file 1 [file 44318_2026_899_MOESM17_ESM.pdf]

Expanded View Figures

Figure EV1. FASS and biochemical analyses of GFP+ and GFP− synapses from vGLUT1^{GFP} mice.

(**A**) Schematic representation depicting synaptosome isolation (i) and FAS sorting (ii) of GFP+ (GFP-positive) and GFP− (GFP-negative) synapses from vGLUT1^{GFP} mice forebrain (m, 1 y) for further biochemical analysis (iii) using ICC and ELISA. (**B**, **C**) Sorting of synapses (singlets, all events, and gating of GFP+ and GFP– synapse populations). Sorted GFP+ synapses were rerun to determine the purity of the sorted population (**C**). Values indicate the mean and standard deviation of 5 experimental replicates, $N = 5$ animals/experiments. (**D**–**M**) Indirect ELISA analyses depicting levels of GFP (**D**), synapsin (**E**), vGLUT1 (**F**), vGAT (**G**), APP-FL (**H**), APP-CT species (**I**), ADAM10 (**J**), BACE1 (**K**), PSEN1 (**L**), and NCSTN (**M**) in all fractions starting from S1, P2, unsorted synapses and sorted synapses. Values were normalized to the S1 fraction of vGLUT1^{GFP} mice. The dotted line indicates average values from blank wells. As seen in the graphs, GFP signals are specific to the transgenic mice and the sorted population of the synapses. The red box highlights data depicted in Fig. 1D,E,G,H,J,K,M,N, respectively. Bar plots represent mean ± SEM; $n = 10$–15, $N = 5$. (**N**) Mander's overlap coefficient of overlap determined from ICC analysis of sorted synapses. vGLUT1/vGAT: GFP $n = 20$, $N = 5$; APP-FL/APP-CT: vGLUT1/vGAT, $n = 10$, $N = 5$; ADAM10/BACE1/PSEN1/NCSTN1: vGLUT1/vGAT. Scatter plots represent individual values; smaller dots, per image; larger dots, experimental replicates; bold line, median ± SEM. $n = 10$, $N = 5$. ns ($P > 0.05$), *($P \le 0.05$), **($P \le 0.01$), ***($P \le 0.001$); one-way ANOVA. Source data are available online for this figure.

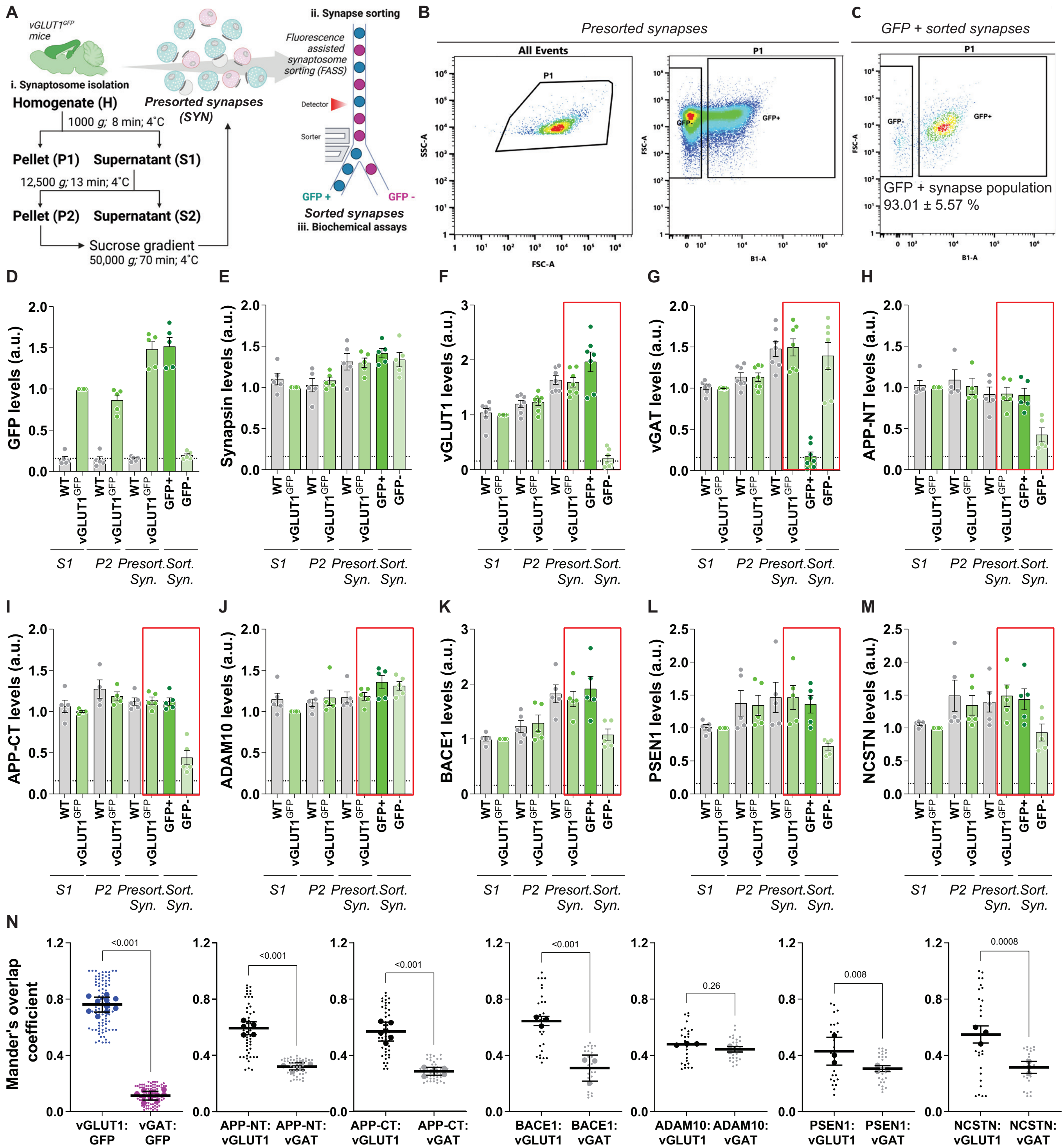
A
vGLUT1GFP mice
i. Synaptosome isolation
Homogenate (H)
1000 g; 8 min; 4˚C
Pellet (P1) Supernatant (S1)
12,500 g; 13 min; 4˚C
Pellet (P2) Supernatant (S2)
Sucrose gradient
50,000 g; 70 min; 4˚C
Presorted synapses (SYN)
ii. Synapse sorting
Fluorescence assisted synaptosome sorting (FASS)
Detector
Sorter
GFP +
GFP -
Sorted synapses
iii. Biochemical assays
B
Presorted synapses
All Events
P1
GFP-
GFP+
C
GFP + sorted synapses
GFP + synapse population
93.01 ± 5.57 %
D GFP levels (a.u.)
E Synapsin levels (a.u.)
F vGLUT1 levels (a.u.)
G vGAT levels (a.u.)
H APP-NT levels (a.u.)
I APP-CT levels (a.u.)
J ADAM10 levels (a.u.)
K BACE1 levels (a.u.)
L PSEN1 levels (a.u.)
M NCSTN levels (a.u.)
WT vGLUT1GFP WT vGLUT1GFP WT vGLUT1GFP GFP+ GFP-
S1 P2 Presort. Syn. Sort. Syn.
N
Mander's overlap coefficient
vGLUT1: GFP vGAT: GFP <0.001
APP-NT: vGLUT1 APP-NT: vGAT <0.001
APP-CT: vGLUT1 APP-CT: vGAT <0.001
BACE1: vGLUT1 BACE1: vGAT <0.001
ADAM10: vGLUT1 ADAM10: vGAT 0.26
PSEN1: vGLUT1 PSEN1: vGAT 0.008
NCSTN: vGLUT1 NCSTN: vGAT 0.0008

 © The Author(s)

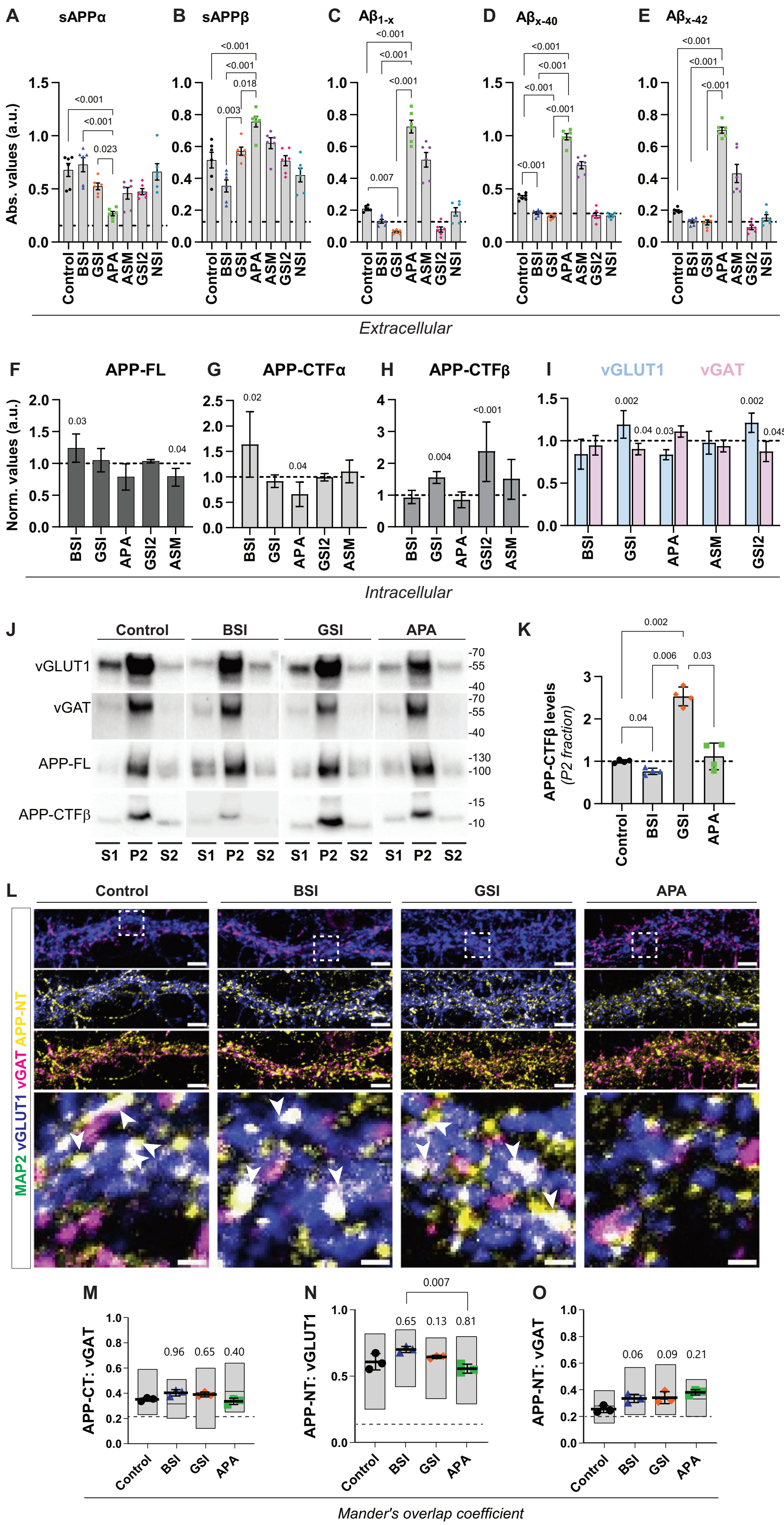
A sAPPα
B sAPPβ
C Aβ1-x
D Aβx-40
E Aβx-42
Abs. values (a.u.)
Control BSI GSI APA ASM GSI2 NSI
<0.001
0.023
0.003
0.018
0.007
Extracellular
F APP-FL
G APP-CTFα
H APP-CTFβ
I vGLUT1 vGAT
Norm. values (a.u.)
BSI GSI APA GSI2 ASM
0.03
0.04
0.02
0.004
0.002
0.045
Intracellular
J
Control BSI GSI APA
vGLUT1
vGAT
APP-FL
APP-CTFβ
S1 P2 S2
K
APP-CTFβ levels (P2 fraction)
Control BSI GSI APA
0.002
0.006
0.03
0.04
L
Control BSI GSI APA
MAP2 vGLUT1 vGAT APP-NT
M
APP-CT: vGAT
0.96 0.65 0.40
N
APP-NT: vGLUT1
0.007
0.65 0.13 0.81
O
APP-NT: vGAT
0.06 0.09 0.21
Control BSI GSI APA
Mander's overlap coefficient

© The Author(s)

◀ **Figure EV2. Pharmacological modulation of APP processing alters the distribution of synaptic proteins in cultured rat neurons.**

(**A–I**) Rat cortical neurons (DIV21) were treated with LY2886721 (1 μM; BACE1 inhibition, BSI), DAPT (10 μM; γ-secretase inhibition, GSI), or Aftin-4 (5 μM; activator of APP processing, APA) for 4 h, to pharmacologically manipulate different stages of APP proteolysis. In addition, we tested GI25423X (1 μM; α-secretase modulator, ASM), Compound E (10 nM; γ-secretase inhibition, GSI2), and FLI-06 (10 μM; inhibitor of the notch signaling pathway, NSI) for 4 h. Post-treatment media were used to detect levels of sAPPα (**A**), sAPPβ (**B**), Aβ (**C**), $A\beta_{x\text{-}40}$ (**D**), and $A\beta_{x\text{-}42}$ (**E**) using indirect ELISA. Bar plots represent mean ± SEM. Neuron homogenate was subjected to western blotting to examine changes in the level of APP-FL (**F**), APP-CTFα (**G**), and APP-CTFβ (**H**) along with vGLUT1 and vGAT (**I**), respectively. Bar plots represent mean ± SEM; $n = 6$, $N = 3$. (**J**, **K**) Neurons post-treatment was subjected to synaptosome enrichment, loaded on western blots to examine changes in levels of proteins between the fractions. While APP-FL remains relatively comparable in the P2 fraction, differences in levels of APP-CTFβ are significant. The dotted line represents the average levels of APP-CTFβ within P2 (synaptic) fraction in untreated conditions. Bar plots represent mean ± SD; $n = 4$, $N = 2$. (**L**) ICC depicting relative changes in the levels of full-length APP (APP-FL) upon treatment with different compounds (gray, yellow-merge). Neurons were also co-stained with vGLUT1 (excitatory synapses, blue), vGAT (inhibitory synapses, magenta), and MAP2 (dendrites, green). Scale bar = 5 μm. Box selection depicts the zoomed-in panel. Arrows depict colocalization/overlap of APP-FL with vGLUT1+ synapse. Scale bar = 1 μm, $N = 3$. (**M–O**) Box plots depicts Mander's overlap coefficient (MOC) of colocalization between APP-FL: vGLUT1 (**N**). MOC values between APP-CT (**M**, images depicted in Fig. 2F) and APP-FL (**O**) with vGAT-positive boutons show no significant changes among treatment groups. Box extends to the minimum and maximum values; center line, mean; $N = 3$. ns ($P > 0.05$), *($P \le 0.05$), **($P \le 0.01$), ***($P \le 0.001$); one-way ANOVA. Source data are available online for this figure.

 © The Author(s)

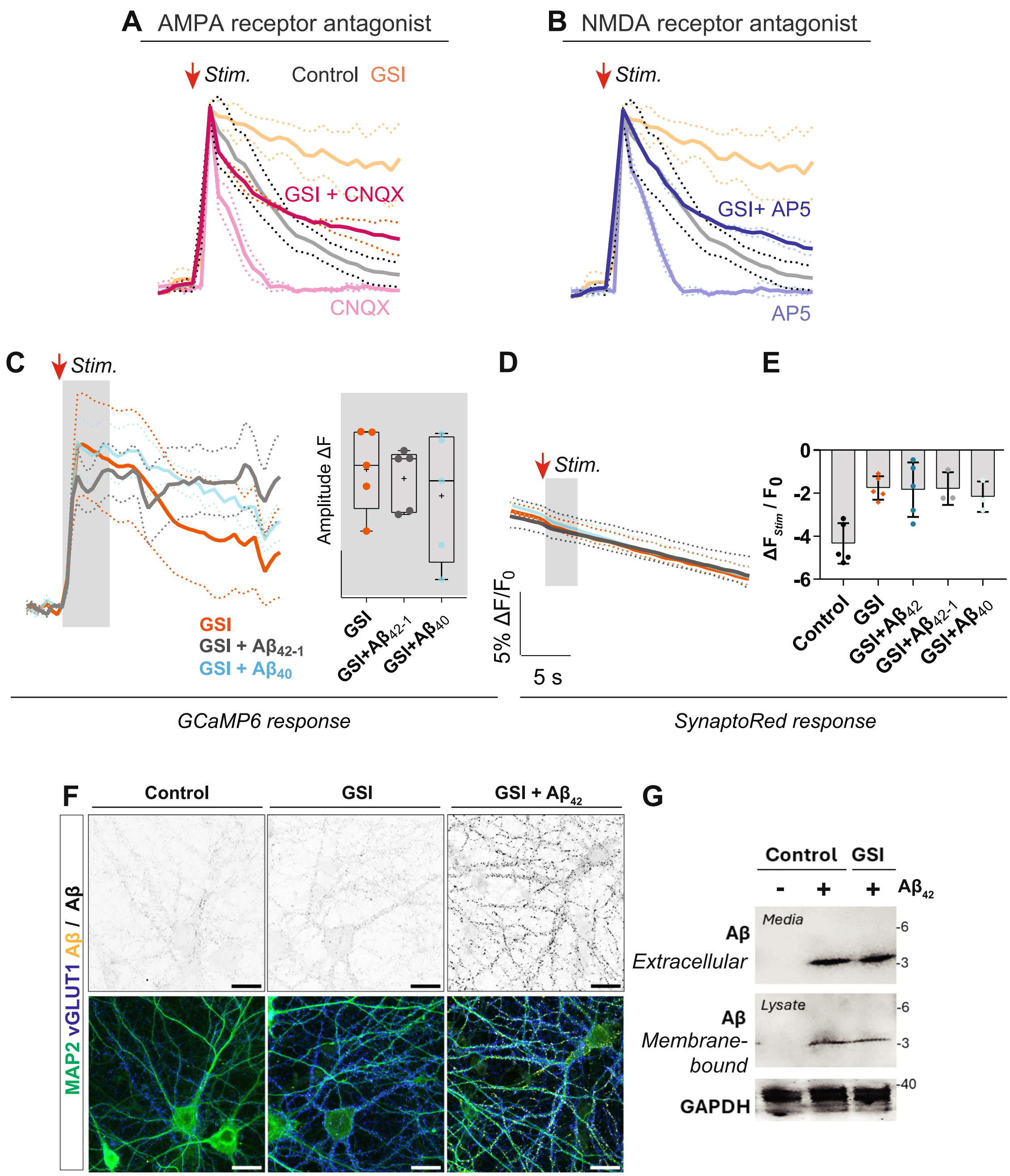

Figure EV3. Effects of post-synaptic receptor antagonism and Aβ42 binding to neuronal membranes.

(**A**, **B**) Neurons expressing Camk2-GCaMP6s were treated with GSI (4 h) were further treated with CNQX (10 µM, 30 min; blocking post-synaptic AMPA receptors), or AP5 (5 µM, 30 min, post synaptic NMDA receptor antagonist) to examine whether changes at postsynaptic signaling influence GSI-induced network hyperactivity. AMPA and NMDA receptor antagonists were added 30 min before the imaging experiments concluding the GSI treatment time point. Experimental values were compared to neurons treated with the inhibitors alone (without GSI) for the respective durations. Traces (**A**, **B**) depict mean (bold) and SEM (dotted lines). $n = 6$, $N = 3$. (**C–E**) As observed in Fig. 3, exposure to 100 nM of $Aβ_{42}$ (5 min) results in rescue of GSI-induced network hyperactivity. In addition, in a similar experimental setting, we examined $Aβ_{40}$ as well as $Aβ_{42\text{-}1}$ (reverse sequence, control peptide), and recorded changes in calcium (**C**) and SR fluorescence response (**D**, **E**), $n = 5$, $N = 2$. Here, no significant change in GCaMP6s response, amplitude of the Ca^{2+} transient, or change in SR fluorescence intensity was detected. (**F**, **G**) Control or GSI-treated or untreated (control) neurons additionally treated with or without $hAβ_{42}$ (100 nM, 5 min) were further analyzed *via* immunocytochemistry (**F**) or western blotting (**G**) with an antibody against Aβ epitope. Neurons were further co-stained with antibodies against vGLUT1 (excitatory synapses, blue) and MAP2 (dendrites, green). $hAβ_{42}$ is mainly detected extracellularly bound to the neuronal membranes. Scale bar = 10 µm. Western immunoblot analyses of protein-enriched samples show that $Aβ_{42}$ in the treatment media and bound to neuronal membranes is monomeric in nature. Also, GSI-induced APP-CTF accumulation did not impact the binding of $Aβ_{42}$ to neuronal membranes. GAPDH was used as a loading control. $N = 2$. ns ($P > 0.05$), *($P \le 0.05$), **($P \le 0.01$), ***($P \le 0.001$); one-way ANOVA. Source data are available online for this figure.

© The Author(s)

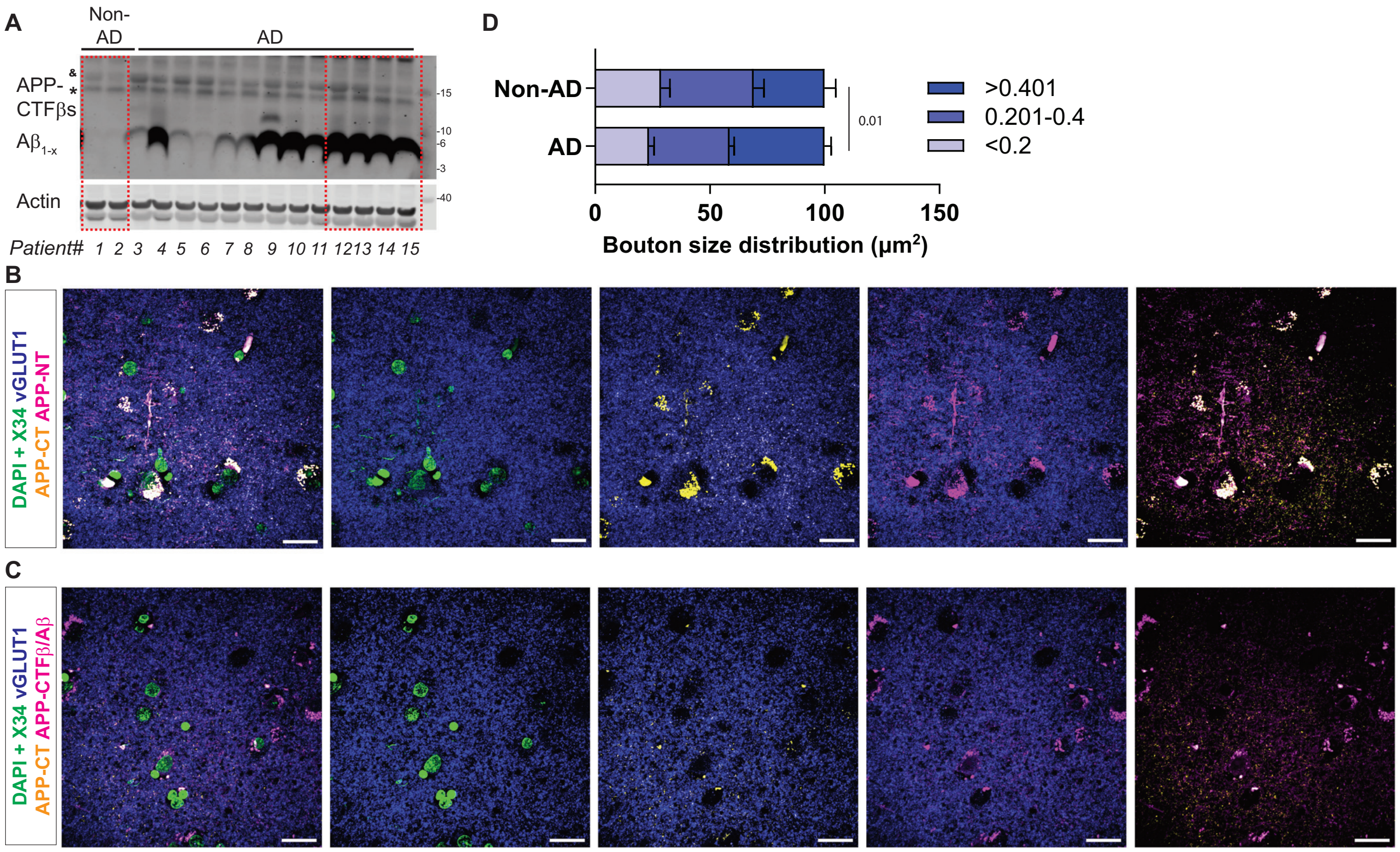

Figure EV4. Biochemical and immunohistochemical analysis of patient samples.

(**A**) Western blot showing non-phosphorylated APP-CTFβ (&) and phosphorylated APP-CTFβ species (*) in the brain homogenates from non-AD controls ($N = 2$) and AD patients ($N = 13$). Red dotted box represents the cropped immunoblot depicted in Fig. 7A. (**B**, **C**) Immunohistochemistry of paraffin-embedded fixed postmortem brain sections from non-AD patients showing APP-CT (clone: 140-rb, **B**, clone: 3125-rb, **C**; yellow) and APP-NT (AB#3, **B**, magenta) or APP-CTFβ/Aβ (clone: 82E1, **C**, magenta) co-stained with excitatory presynaptic marker vGLUT1 (blue) and DAPI (green), with X34 labeling amyloid plaque deposits if any (green); $n = 2$, $N = 2$ independent experiments. (**D**) Cumulative bar plots depict the distribution of presynaptic bouton areas in non-AD versus AD patient samples, $n = 2$, $N = 2$ independent experiments. Source data are available online for this figure.

 © The Author(s)
