# Supplementary material for: Presynaptic accumulation of APP-CTFβ may contribute to synaptic dysfunction in Alzheimer’s disease
Source: EMBO J. 2026 Sep 1;45(19):6914–45. doi: 10.1038/s44318-026-00899-6 (PMC13631370; doi:10.1038/s44318-026-00899-6)
Supplement: Supplementary file 3 [file 44318_2026_899_MOESM2_ESM.pdf]

THE EMBO JOURNAL

Presynaptic accumulation of APP-CTFβ may contribute to synaptic dysfunction in Alzheimer's disease

Akshay Kapadia, Fabian Schuhmann, Ezgi Daskin, Jochen Walter, Isabell Lindahl, Neda Rahmani, Tjado Morrema, annemieke rozemuller, Baayla Boon, Weria Pezeshkian, and Anne-Sophie Hafner

Corresponding author(s): Anne-Sophie Hafner (anne-sophie.hafner@donders.ru.nl) , Akshay Kapadia (akshay.kapadia@donders.ru.nl)

Review Timeline:

Submission Date:	10th Feb 26
Editorial Decision:	13th Mar 26
Revision Received:	24th Apr 26
Editorial Decision:	3rd Jul 26
Revision Received:	12th Jul 26
Accepted:	23rd Jul 26

Editor: Ioannis Papaioannou

Transaction Report:

(Note: With the exception of the correction of typographical or spelling errors that could be a source of ambiguity, letters and reports are not edited. Depending on transfer agreements, referee reports obtained elsewhere may or may not be included in this compilation. Referee reports are anonymous unless the Referee chooses to sign their reports.)

Dear Dr. Hafner,

Thank you for the submission of your manuscript EMBOJ-2026-123845 for consideration by The EMBO Journal, and for your patience during peer review. Your manuscript has now been seen by three experts in the field, and we have received the full set of their reports, which you can find appended below.

As you will see, the referees indicate interest in your study, acknowledge the relevance of the topic and the question addressed, and recognize the potential significance of the results. However, they also identify a number of limitations and raise several concerns. Importantly, there is consensus among them that not all relevant literature has been appropriately cited and discussed, and that the interpretation and presentation of your results are not entirely balanced and remain to some extent speculative. They also raise technical concerns, regarding for example the specificity of the used antibodies and some controls, among other points.

On balance, our decision was that we would like to invite you to submit a substantially strengthened revised version of your manuscript taking the referees' comments and suggestions on board. I should note that for the revised version to be considered further for publication in The EMBO Journal, the text must be carefully revised throughout the manuscript to present, contextualize and discuss the results in a balanced manner, citing all relevant studies, clarifying the novelty of the new study over the literature, and acknowledging the possibility that other processes may also contribute to the disease initiation. The conclusions of the study should be fully supported by the available data. Any discussion of more speculative nature should be clearly presented as such in the Discussion section of your revised manuscript. Furthermore, the revision should convincingly address all technical issues raised by the referees.

Along with your revised manuscript, please also prepare and submit a detailed point-by-point response fully addressing all referee comments. Please note that it is The EMBO Journal policy to allow only a single round of major experimental revision, and acceptance of your manuscript will therefore depend on the completeness of your responses in this revised version and your point-by-point response. Given the nature of the raised concerns, I think it would be very useful to discuss your revision plan and its feasibility already during the initial phase of your revision. You are very welcome to share with me a draft point-by-point response letter/revision plan explaining how you would try to address the referees' concerns, and if there are any points you do not agree with or cannot address. I would also be happy to arrange a video call with you to discuss the details further if this might be useful.

We generally allow three months as standard revision time (12 June, 2026). As a matter of policy, competing manuscripts published during this period will not negatively impact our assessment of the conceptual advance presented by your study. However, we request that you contact us as soon as possible upon publication of any related work, to discuss how to proceed. Should you foresee a problem in meeting this three-month deadline, please let us know in advance and we will be able to grant an extension.

Please let me know if you have any questions or comments that you would like to discuss with me. Thank you for the opportunity to consider your work for publication in The EMBO Journal. I look forward to your revision.

Best regards,

Ioannis

Ioannis Papaioannou, PhD
Editor, The EMBO Journal
i.papaioannou@embojournal.org

*****
Instructions for preparing your revised manuscript
*****

1. When you are ready to submit the revision, please upload:

- A Word file of the manuscript text (including legends of main Figures, EV Figures and Tables). Please make sure that changes are highlighted (or "tracked") to be clearly visible.

- Individual production-quality figure files (one file per figure). When assembling your figures, please refer to our figure preparation guidelines in order to ensure proper formatting and readability in print as well as on screen:

https://bit.ly/EMBOPressFigurePreparationGuideline
If the data shown in a figure are obtained from n {less than or equal to} 2, please use scatter plots showing the individual data points.
Please also include scale bars in all microscopy images.
The following points must be specified in each figure legend:
i. the name of the statistical test used to generate error bars and P values
ii. the number (n) of independent experiments (please specify technical or biological replicates) underlying each data point (discussion of statistical methodology can be reported in the Materials and Methods section, but figure legends should contain a basic description of n, P, and the test applied)
iii. the nature of the bars and error bars (s.d., s.e.m.).
See also guidelines for figure legends: https://www.embopress.org/page/journal/14602075/authorguide#figureformat

- A point-by-point response to the referees' comments, with a detailed description of the changes made (as a word file). All referees' concerns must be fully addressed and their suggestions taken on board. When preparing your letter of response to the referees' comments, please bear in mind that this will form part of the Review Process File and will therefore be available online to the community. Please note that you have the possibility to opt out of the transparent process at any stage prior to publication by letting the editorial office know (contact@embojournal.org); if you do opt out, the Review Process File link will point to the following statement: "No Review Process File is available with this article, as the authors have chosen not to make the review process public in this case.". For more details on our Transparent Editorial Process, please visit our website: https://www.embopress.org/page/journal/14602075/authorguide#transparentprocess

- Expanded View (EV) files (replacing Supplementary Information) that are collapsible/expandable online. A maximum of 5 EV Figures can be typeset. EV Figures should be cited as "Figure EV1, Figure EV2" etc. in the text, and their respective legends should be included in the manuscript file after the legends of regular figures. See detailed instructions regarding Expanded View files here:
https://www.embopress.org/page/journal/14602075/authorguide#expandedview

- For the figures that you do NOT wish to display as Expanded View figures, they should be bundled together with their legends in a single PDF file called "Appendix", which should start with a short Table of Contents (including page numbers). Appendix figures should be referred to in the main text as: "Appendix Figure S1, Appendix Figure S2" etc. Please see detailed instructions here: https://www.embopress.org/page/journal/14602075/authorguide#expandedview

- A complete author checklist, which you can download from our author guidelines (https://www.embopress.org/page/journal/14602075/authorguide). Please note that the checklist will also be part of the Review Process File.

2. Please note that no statistics should be calculated and shown in Figures if n=2. Please also note that each p value should be reported as an exact value.

3. Before submitting your revision, primary datasets (and computer code, where appropriate) produced in this study need to be deposited in appropriate public databases (see https://www.embopress.org/page/journal/14602075/authorguide#dataavailability). In particular, all datasets such as DNA or RNA sequencing data, or mass spectrometry data generated in the study must be deposited in appropriate repositories. The accession numbers, database, and the specific URLs (links) should be listed in a formal "Data availability" section (placed after Methods), following the example below:

"The RNA-seq datasets produced in this study are available in the following database:
Gene Expression Omnibus GSE46843 (https://www.ncbi.nlm.nih.gov/geo/query/acc.cgi?acc=GSE46843)"

*** All links should resolve to a page where the data can be accessed. ***

*** Please remember to provide in the Data availability section of your revised manuscript reviewer passwords if the datasets are not yet public. ***

*** The Data Availability Section is restricted to new primary data that are part of this study. In case you have no data that require deposition in a public database, please state so instead of referring to the database: "Our study includes no data deposited in public repositories." under the heading "Data availability". ***

4. The materials and methods need to be described in the manuscript using our structured methods format, which is now required for all research articles. According to this format, the Methods section includes a single "Reagents and Tools Table" - listing key reagents, experimental models, software and relevant equipment including their sources and relevant identifiers- followed by a "Methods and Protocols" section describing the methods. Please download and fill our Reagents and Tools Table template (.docx), which you can find in our author guide:
https://www.embopress.org/page/journal/14602075/authorguide#structuredmethods. When submitting your revised manuscript, please do not include the Reagents and Tools Table in the Methods section of the manuscript but instead upload it as a

separate file choosing the file type "Reagent Table".

5. Please check that the title and the abstract of the manuscript are brief, yet explicit, even to non-specialists. The length of the title should not exceed 100 characters, and the abstract should be a single paragraph not exceeding 175 words.

6. Please also note our reference format: https://www.embopress.org/page/journal/14602075/authorguide#referencesformat.

7. At EMBO Press we ask authors to provide source data for the main manuscript figures. You will receive a separate email with instructions for providing source data with your revised manuscript, including how to upload and organize the files.

8. Please remember: digital image enhancement is acceptable practice, as long as it accurately represents the original data and conforms to community standards. If a figure has been subjected to significant electronic manipulation, this must be noted in the figure legend or in the "Materials and Methods" section. The editors reserve the right to request original versions of figures and the original images that were used to assemble the figure.

9. Our journal encourages inclusion of data citations in the reference list to directly cite datasets that were obtained from public databases. Data citations in the article text are distinct from normal bibliographical citations and should directly link to the database records from which the data can be accessed. In the main text, data citations are formatted as follows: "Data ref: Smith et al, 2001" or "Data ref: NCBI Sequence Read Archive PRJNA342805, 2017". In the Reference list, data citations must be labeled with "[DATASET]". A data reference must provide the database name, accession number/identifiers, and a resolvable link to the landing page from which the data can be accessed at the end of the reference. Further instructions are available at: https://www.embopress.org/page/journal/14602075/authorguide#referencesformat.

10. We request authors to consider both actual and perceived competing interests. Please review our policy (https://www.embopress.org/page/journal/14602075/authorguide#conflictsofinterest) and update your competing interests statement if necessary. Please name this section 'Disclosure and competing interests statement' and place it after the Acknowledgements section.

11. Please note that all corresponding authors are required to provide an ORCID ID upon submission of a revised manuscript (https://orcid.org/). Please find instructions on how to link your ORCID ID to your account in our manuscript tracking system in our Author guidelines (https://www.embopress.org/page/journal/14602075/authorguide#authorshipguidelines).

12. We use CRediT to specify the contributions of each author in the journal submission system. CRediT replaces the author contribution section, which should be removed from the manuscript. Please use the free text box to provide more detailed descriptions. See also guide to authors: https://www.embopress.org/page/journal/14602075/authorguide#authorshipguidelines.

13. Further information is available in our Guide For Authors: https://www.embopress.org/page/journal/14602075/authorguide

14. We would also welcome the submission of cover suggestions or motifs to be used by our Graphics Illustrator in designing a cover.

Referee #1:

This is a very interesting and technically high-level manuscript working to define the role of APP CTFs- specifically C99)- in the presynapse. A large body of work has shown that APP, its metabolites and amyloidogenic processing components are present and active at synapses, including presynapses, where APP appears to be preferentially trafficked to and processed. The accumulation of APP CTFs in neuritic dystrophies (typically axonal, and also presynaptic) in Alzheimer's disease (AD) brains has been known for about 45 years. The transport to and processing of APP at synapses further suggests a function there, which this manuscript provides several important new insights into.

Major point
This study is from very competent neuroscientists, who have been influenced by a hypothesis that is a minority view, which is that APP CTFs rather than Abeta42/43 initiate the AD disease process. This reviewer believe that both are involved in initiating

AD. However the role for Abeta42/43 in AD is significantly greater than the authors describe in their manuscript. A potential issue with this otherwise excellent work is that many but far from all of their work relies on chemical treatments which can be tricky and can be more complex than they might seem. Inhibitors do not purely inhibit the step one wants them to. GSI and BSI have other substrates, while APA is less well understood. To their credit, they show that "To ensure that excitatory synapse enlargement was due to APP-CTFβ accumulation and/or Aβ decrease and not to another substrate of γ-secretase, we repeated this experiment while knocking down APP using siRNA (fig. S10). The effect was not observed in neurons transfected with siRNA targeting APP indicating that APP-CTFβ accumulation and/or Aβ decrease are driving the increase in excitatory synapse size" This is an important experiment and better than just relying on GSI, as others who have supported CTFs have tended to do. The APP shRNA could have had a shRNA control, since the procedure can itself have effects in neurons. Nevertheless, it is a clear strength, although it's not used as a control for all their experiments with these treatments.
When they suggest that CTFs might be important to AD initiation separate from extracellular Abeta, they may not be aware about the extensive literature on intracellular and intra-synaptic Abeta, which could place CTFs and Abeta42 together in causing synapse alterations that initiate AD: see e.g. Bilousova T et al., 2016; Eckmann EA et al., 2023: Takahashi RH et al., 2002; Kobro-Flatmoen A et al., 2016; Yuan P, Zhang N, Tong L, Morse TM et al., 2024. This intraneuronal pool of Abeta is more difficult to detect, however, low levels can still be potent.

Related point
Regarding the first sentence of their Introduction: Abeta is not only considered important for AD because of its aggregation propensity and it being the building block of plaques." There is significantly more important evidence, especially since plaques don't correlate well with cognitive decline. The hundreds of genetic mutations causing familial AD all point to Abeta; in contrast to plaques, soluble Abeta42 levels in brain correlate with cognitive decline (McLean CA et al., 1999; Lue LF et al., 1999), and a drop in Abeta42 (not changes in CTFs) is the earliest validated biomarker for AD. In the Discussion they go into the protective Icelandic variant and the Swedish mutation to argue for CTFs but fail to mention the numerous mutations affecting the C-terminus of Abeta that lead to elevated levels of more aggregation-prone Abeta42 or 43. Recently it was also reported that even when a presenile 1 mutation leads to inability to cleave, a heterozygous mouse model with such a mutation still leads to more Abeta42 (from WT PS1 and PS2s) (Sanjuan-Ruiz I et al., 2025). Further down they cite a literature suggesting Abeta-independent effects of APP CTFs. A key experiment used in many of these studies is complex. After showing toxic effects in cells that have elevated CTFs but also Abeta, treatment with GSI is used to see if by blocking Abeta production, the toxicity might be rescued. Because this doesn't happen, Abeta independence is argued for. GSI is not a trivial treatment. Without gamma-secretase activity, mouse embryos die in utero (while attributed to loss of Notch signaling, numerous important proteins -over a hundred including important synaptic proteins, e.g. in their citation of Restituito S et al., 2011- rely on this cleavage. Thus, it can be tricky to know how GSI is acting. An experiment used in the current manuscript is however much stronger in that they also give GSI this in the presence of APP knockdown.

Other points
Further down in the Discussion they write "Therapeutic strategies singularly aimed at reducing Aβ levels or its aggregation have faced significant challenges in clinical trials, making AD drug development a pharmaceutical graveyard." That is not totally untrue but one should also keep in mind that reducing Abeta has also been the first and only therapy that showed clinical benefits in blinded clinical studies. In contrast, non-Abeta targets have so far failed 100% of the time in slowing down the disease.

They should provide a bit more information about Aftin-4 (APA). Most are familiar with beta and gamma-sec inhibitors but the mechanism of action of APA-4 appears complex and not fully clear. APA was reported to increase Abtea42 but not 40 and even to lower Abeta38.

It isn't clear from the manuscript what form of Abeta they used when they first use it: was it Abeta1-42? This starts aggregating immediately in solution such as their 100 nM solution and even more in their earlier 230 microM solution; 10 minutes of sonication will not dissolve it all to monomers. Also 100 nM is 1000 fold higher than physiological for extracellular Abeta42, which for the extracellular fluid CSF is 100-250 pM. Thus, their use of the term "monomeric" is not ideal. It is likely a mixture of monomers and various oligomeric species. Running it on a non-denaturing gel would give information about this. Later in the Results they do experiments where they treat separately with 42 and other Abeta peptides, but the precise species the use should be clear throughout.

On line 28 of the Discussion they write "We show that in endogenous conditions, APP and its amyloidogenic processing components are predominantly localized to excitatory presynaptic terminals." Although it is well known that APP is at synapses and traffics there by fast axonal transport, that does not mean most is there. Labeling with an APP CTF specific antibody typically shows most labeling in the area of the Golgi apparatus and in transport and endosomal/autophagic vesicles throughout the cell with eventual degradation in lysosomes. It is also long known that APP processing occurs heavily at synapses where beta- and gamma-secretases have been shown to reside. Remarkably, synaptic activity was shown to promote axonal transport to synapses and generation of Abeta.

They might take a look at a few more recent articles relating to AD ,hyperexcitability and synapses, such as Papanikolaou A et al., 2025, Wilson P, Kim N, et al.2025, Martinsson I et al., 2022, Zarhin D et al., 2022, Zott D et al., 2019, and even Wei W, Nguyen LN et al., 2009, amongst others.

Referee #2:

Review report to Kapadia et al. EMBOJ-2026-123845

Summary
This study by Kapadia et al. investigates the synaptic localization of full length APP-FL and its proteolytic products, in particular that of APP-C-terminal fragments (CTFs) to elucidate whether a presynaptic accumulation of CTFs causes presynaptic dysfunction and whether this results in early hyperexcitability in AD.
To this end the authors use fluorescent activated synaptosome sorting from mice with a GFP-knockin into the vGlut1-locus to enrich for synaptosomes from excitatory synapses. They identify in these preparations an enrichment of APP-FL, APP-CTFs as well as all relevant secretases. They investigate the subcellular localization of APP-FL and APP-CTFs and report a selective enrichment of APP-CTFs as compared to APP-FL in presynaptic compartments that is further enhanced by inhibition of amyloidogenic processing. This leads to enhanced synaptic vesicle release probability and thus deficits in vesicle release, as revealed by Ca2+ imaging, SynaptoRed- and Syp-pHflourin imaging in primary neurons. Using immunoisolation the authors go on to show that APP-CTFs associate with the active zone and that this can be enhanced under GSI conditions.

General remarks
The concept that accumulation of APP-CTFs can drive presynaptic dysfunction is not novel. In fact, Barthet et al. 2018 (PMC6235831, see also review by Barthet and Mulle, 2020) previously analyzed presynaptic function in mice with a presynapse-specific KO of PS1/PS2 at Mossy fiber to CA3 synapses. This resulted in impaired presynaptic facilitation and impaired replenishment of the synaptic vesicle pool, which was associated with a drastic reduction in the Ca2+ sensor Synaptotagmin 7. Importantly, it was further shown (using APP-KO/PS1-KO mice) that the depletion of Syt7 was dependent on APP, specificaly the accumulation of APP-CTFs at presynaptic sites. This in vivo study was not quoted nor discussed by the authors. The authors need to address in detail how their study, conducted in vitro, adds conceptually to this previous knowledge. The claim of conceptual novelty that APP-CTF accumulation drives presynaptic dysfunction is therefore not justified.

In addition, there are major technical limitations (see below) that compromise the conclusions drawn and preclude publication in its present form.

Specific comments:

Point 1: Related to Fig1, but also applies to all other figures. The study essentially relies on antibodies used in WB, ICC, ELISA and proteomics to deduce the specific localization of APP and its fragments at the synapse.

A major concern is therefore the specificity of the Abs for APP-FL and CTFs that are key for the whole study. Here, it is essential to provide experimental evidence, that is currently lacking, that Abs recognize with high specificity and selectivity the claimed fragments and to exclude cross-reactivity with APP-FL (see my comments for example for M3.2 below). Only antibodies directed against a cleavage specific neo-epitope allow to distinguish unambiguously between CTFs and APP-FL. All other antibodies directed at the C-terminus will inevitably also recognize APP-FL, which is particularly relevant in ICC or IHC where the size of Fragments cannot be relvealed.

If stainings with different antibodies are compared, e.g a staining directed against an N-terminal epitope (e.g. polyclonal rabbit SAB4200536) versus a staining using a C-terminal epitope, differences in localization to a certain subcellular structure may result from differences in the affinity and thus sensitivity of the antibodies. Lack of presynaptic staining (or less intense staining) with the N-terminal Ab may result from insufficient sensitivity. This issue needs to be addressed and excluded experimentally.

In each figure panel it must be specifically stated which Ab from the long list was used (e.g. 4 different Ab are directed against the C-terminus).
Moreover, for ICCs negative controls for background staining need to be provided and APP-KO neurons should be used to exclude unspecific signals, that have been notoriously found in the AD field.

In addition, the authors should generate a schematic figure depicting the various APP processing products and antibodies used to distinguish between them or indicate overlapping reactivity.

In the methods section on page 19 the antibody M3.2 is indicated as APP-C-terminus specific. This is actually not true as the antibody binds between the b- and a-secretase site and will therefore detect both APP-FL and CTFb. Also C1/6.1 will detect both APP-FL and CTFs

Specific comments to Fig 1 O : It is unclear how APP-FL was distinguished from APP-CTFs (CTFa or CTFb?) using ICC on primary neurons, please explain this essential point and provide additional data (see my remarks above). The M3.2 antibody , as well as C1/6.1, is not suited for distinguishing APP-FL from APP-CTFs (see above). Also the details provided in Table S1 and

S2 are not sufficient and need to be further validated by showing experimental data for the specificity/epitope of each antibody. This also applies to in house generated antibodies (JW, Eurogentec 3125 and 140-rb).
Point2, related to Fig 1: The authors state that most vGAT -enriched synapses (GFP) did not contain APP, APP-CTFs, BACE and PSEN. This statement, also made in the abstract, needs to be toned down as panels Fig 1G-N clearly show that these proteins were pretty abundant and detected at about 50% in GFP-negative synapses. There is also evidence from studies using interneuron-specific BACE-KO models that a large proportion of Abeta is generated by interneurons (e.g Rice et al., PMC6950898) and scRNAseq data show the co-expression of APP and of the secretase machinery in interneuron populations.
Point 3, related to Figure 2: the authors use various secretase inhibitors to modulate the abundance of APP fragments in particular APP-CTFs. In Fig 2F it is again unclear how APP-FL and APP-CTFs were distinguished. Please also specify how the biological replicates were obtained, what means N=4?
Point 4, related to Figure S3, panel A-E: ELISA was used to monitor the specificity and efficiency of secretase inhibitors. It appears that the data are not always supporting the expected modulation of the pathways. e.g the ASM (a-secretase inhibitor) did not significantly reduce sAPPa and BSI did not reduce sAPPb. The same is true for WBs of neuronal homogenates (Fig S3G, H): ASM and GSI did not modulate levels of APP-CTFa and BSI did not modulate CTFb, respectively. These pharmacological agents have previously been used by many labs in primary neurons with the expected outcomes, also without enrichment for synaptosomes. The ELISA used is not the most sensitive available in the field (e.g. compared to MSD ELISAs). These shortcomings need to be improved, or the focus should shift to GSI inhibitors only, that showed robust data.
Point 5, related to Figure 2 G.
The authors assess synapse size from ICC in primary neurons by calculating the vGlut1 positive area. This needs to be substantiated with an independent measure of synapse size.

There is also concern that vGlut1 positive puncta detected in primary neurons do not necessarily represent mature and active synapses. Staining with a postsynaptic marker is needed to substantiate these findings and all conclusions drawn from it.

Point 6, related to Fig 5A,B. Please describe in more detail the blots depicted in panels A and B , the labelling of the Y axis and how the quantitative evaluation of the data was performed.

Minor points
1) The paper focuses primarily on CTFb, although the physiologically most relevant pathway in wildtype neurons is CTFa. Please comment and explain.
2) The discussion is in part highly speculative and rather general (e.g. Page 15, lines 8-17) It would greatly benefit from a clear focus that is more closely related to the data presented.
3) Page 15, line 20: check wording and grammar

Referee #3:

- general summary and opinion about the principle significance of the study, its questions and findings
APP, the mother protein of peptide Abeta, is expressed in most of brain cells. Its processing is highly complex, modulated by different consecutive cleavages by different enzymes as BACE, ADAM and gamma-secretase. The authors address a very important questions in the field of neurophysiology and in particular in the context of Alzheimer's disease. Is there a cell type difference in what regards to the expression of APP and APP processing ? If so, will this difference have a functional impact, which could be related to AD pathology if APP processing is changed ? Which APP fragment would be the culprit contributing the most to AD ?
To my knowledge the authors presented convincing data to these questions with elegant technical approaches.

- specific major concerns essential to be addressed to support the conclusions
I don't have major concerns.

- minor concerns that should be addressed
the study would benefit from an experiement showing a the observed prevalence of APP and BACE expression in excitatory neurons from a human model, thus allowing translation of the observed results. The authors show the increased expression of APP-CTFbeta in AD brains through electronic microscopy, but these data does not convincingly show that APP and amyloidogenic pathway is prevalent in excitatory neurons indetriment to inhibitory neurons.

- any additional non-essential suggestions for improving the study (which will be at the author's/editor's discretion)
The discussion presents several sentences that are quite speculative. I would suggest the authors to restrain the discussion to the data in regard to the literature. Ex. Line 8 to 17, page 15.

We thank the editor for the careful evaluation of our manuscript- EMBOJ-2026-123845, entitled 'Alzheimer's disease related accumulation of APP-CTFβ drives presynaptic dysfunction'. We are grateful for the time and expertise involved in accessing our work. We appreciate the reviewers' assessment of the relevance of the topic, technical breadth of the study, as well as the potential significance of the findings. We appreciate the constructive comments and suggestions which have significantly improved the manuscript in its revised form.

All comments and suggestions raised by the reviewers have been addressed (indicated in blue). In particular, we have revised the text throughout to ensure a balanced presentation and interpretation of the data, rephrased speculative statements, clarified the scope and novelty of the study in the context of the existing literature data. The revised discussion section of the manuscript expands on complementary mechanisms contributing to AD, to better position our findings in the current state of the field.

In addition, technical concerns raised during review regarding antibody usage and specificity, figure legends, methodological descriptions, interpretation of imaging data, and respective controls have been addressed to strengthen the rigor and transparency of the study.

We are pleased to herewith submit a substantially strengthened revised version of the manuscript and the SI file, along with a detailed point-by-point response to all reviewer comments answered below.

Referee #1:

This is a very interesting and technically high-level manuscript working to define the role of APP CTFs- specifically C99)- in the presynapse. A large body of work has shown that APP, its metabolites and amyloidogenic processing components are present and active at synapses, including presynapses, where APP appears to be preferentially trafficked to and processed. The accumulation of APP CTFs in neuritic dystrophies (typically axonal, and also presynaptic) in Alzheimer's disease (AD) brains has been known for about 45 years. The transport to and processing of APP at synapses further suggests a function there, which this manuscript provides several important new insights into.

Author response: We thank the reviewer for their thoughtful comments and appreciate their encouraging assessment of our work.

Major point

This study is from very competent neuroscientists, who have been influenced by a hypothesis that is a minority view, which is that APP CTFs rather than Abeta42/43 initiate

the AD disease process. This reviewer believe that both are involved in initiating AD. However the role for Abeta42/43 in AD is significantly greater than the authors describe in their manuscript.

Author response: We agree with the reviewer, AD is a complex, multifactorial disorder in which both Aβ-species and APP/APP-CTFs/β contribute to disease initiation and progression. We have revised the manuscript significantly to reflect on these aspects while positioning our work within the broad framework of AD, highlighting an additional complementary mechanism triggering early AD onset.

A potential issue with this otherwise excellent work is that many but far from all of their work relies on chemical treatments which can be tricky and can be more complex than they might seem. Inhibitors do not purely inhibit the step one wants them to. GSI and BSI have other substrates, while APA is less well understood. To their credit, they show that "To ensure that excitatory synapse enlargement was due to APP-CTFβ accumulation and/or Aβ decrease and not to another substrate of γ-secretase, we repeated this experiment while knocking down APP using siRNA (fig. S10). The effect was not observed in neurons transfected with siRNA targeting APP indicating that APP-CTFβ accumulation and/or Aβ decrease are driving the increase in excitatory synapse size" This is an important experiment and better than just relying on GSI, as others who have supported CTFs have tended to do. The APP shRNA could have had a shRNA control, since the procedure can itself have effects in neurons. Nevertheless, it is a clear strength, although it's not used as a control for all their experiments with these treatments.

Author response: We thank the reviewer for highlighting this important aspect of using the pharmacological approaches (inhibitors/modulators) that affect multiple substrates beyond APP. To address this, we have employed several complementary strategies throughout the study:
(i) use of other pharmacological modulators targeting distinct steps of APP processing, both amyloidogenic and non-amyloidogenic (ASM and Notch signaling)- Ext. Fig. 2A-H, SI Fig, S2D, S5E-I.
(ii) importantly use of siRNA reducing APP levels helps us distinguish APP-related and unrelated effects, specifically when inferring the effects of APP-CTFs/β-accumulation, which is an important part of this study SI, Fig. S7. Here, we restricted the APP knockdown experiments to conditions where key interpretations were made. This provides observed effects that are specifically linked to APP-CTFs/β and/or APP-processing- rather than off-target drug effects.

When they suggest that CTFs might be important to AD initiation separate from extracellular Abeta, they may not be aware about the extensive literature on intracellular

and intra-synaptic Abeta, which could place CTFs and Abeta42 together in causing synapse alterations that initiate AD: see e.g. Bilousova T et al., 2016; Eckmann EA et al., 2023: Takahashi RH et al., 2002; Kobro-Flatmoen A et al., 2016; Yuan P, Zhang N, Tong L, Morse TM et al., 2024. This intraneuronal pool of Abeta is more difficult to detect, however, low levels can still be potent.

Author response: We agree, intracellular pools of Aβ, although technically challenging to detect (Wilson, Doms, and Lee 1999; Oddo et al. 2006; Skovronsky, Doms, and Lee 1998; Gouras, Willén, and Tampellini 2012), have been widely reported and exert potent synaptic effects even at lower concentrations (Cavallucci, D'Amelio, and Cecconi 2012; Siddu et al. 2025). This has now been cited in the discussion aspect of the manuscript wherein we indicate that the shift in physiology-to-pathology effect of Aβ is concentration dependent.

We would also like to point out that the intracellular pools of Aβ could also be in some part APP-CTFβ species which are cross-detected using antibodies that could indirectly detect other species (S. Hunter and Brayne 2017).
Additionally, we would like to point out that the effects we report linked to the accumulation of APP-CTFβ after inhibition of γ-secretase occur while Aβ levels are significantly reduced - as this is also a consequence of our treatment (see Figure 2A-E). Thus, the increased network activity in response to increased levels of APP-CTFβ is undoubtedly independent of Aβ which is not being produced anymore.

Related point
Regarding the first sentence of their Introduction: Abeta is not only considered important for AD because of its aggregation propensity and it being the building block of plaques." There is significantly more important evidence, especially since plaques don't correlate well with cognitive decline. The hundreds of genetic mutations causing familial AD all point to Abeta; in contrast to plaques, soluble Abeta42 levels in brain correlate with cognitive decline (McLean CA et al., 1999; Lue LF et al., 1999), and a drop in Abeta42 (not changes in CTFs) is the earliest validated biomarker for AD. In the Discussion they go into the protective Icelandic variant and the Swedish mutation to argue for CTFs but fail to mention the numerous mutations affecting the C-terminus of Abeta that lead to elevated levels of more aggregation-prone Abeta42 or 43. Recently it was also reported that even when a presenile 1 mutation leads to inability to cleave, a heterozygous mouse model with such a mutation still leads to more Abeta42 (from WT PS1 and PS2s) (Sanjuan-Ruiz I et al., 2025). Further down they cite a literature suggesting Abeta-independent effects of APP CTFs. A key experiment used in many of these studies is complex. After showing toxic effects in cells that have elevated CTFs but also Abeta, treatment with GSI is used to see if by blocking Abeta production, the toxicity might be

rescued. Because this doesn't happen, Abeta independence is argued for. GSI is not a trivial treatment. Without gamma-secretase activity, mouse embryos die in utero (while attributed to loss of Notch signaling, numerous important proteins -over a hundred including important synaptic proteins, e.g. in their citation of Restituito S et al., 2011- rely on this cleavage. Thus, it can be tricky to know how GSI is acting. An experiment used in the current manuscript is however much stronger in that they also give GSI this in the presence of APP knockdown.

Author response: We agree that γ-secretase inhibition using pharmacological treatments is difficult to interpret due to the broad substrate and essential biological roles. Combining multiple perturbations within a single experiment paradigm increases biological complexity and limits interpretability. Thus, our stepwise approach using complementary strategies where key interpretations were made to specifically disentangle APP-processing-APP-CTFs rather than off-target drug effects.

Other points

Further down in the Discussion they write "Therapeutic strategies singularly aimed at reducing Aβ levels or its aggregation have faced significant challenges in clinical trials, making AD drug development a pharmaceutical graveyard." That is not totally untrue but one should also keep in mind that reducing Abeta has also been the first and only therapy that showed clinical benefits in blinded clinical studies. In contrast, non-Abeta targets have so far failed 100% of the time in slowing down the disease.

Author response: While Aβ remains the only clinically validated target till date, monoclonal Aβ-targeting antibody therapy has shown significant success in clearing Aβ load in patient brains, but the positive changes in memory and cognition remain modest (P. Hunter 2024; Panza et al. 2025; Lozupone et al. 2025; Chundu et al. 2025). Currently this is attributed to the fact that clinical trials cohorts were late stage AD patients, attributed as the "too late" hypothesis; and thus, there is a push to apply antibody therapy to patients in the early stages of the disease (Guiloff and Rudge 2024; van Dyck et al. 2023; Honig et al. 2024). Our experiments show that if monomeric Aβ is removed at early stages of the disease it may shift/change the stoichiometry of APP and its proteolytic products, which could have detrimental effects on synapse and neuron homeostasis. Taken together, we thus propose that there is an unmet need in finding effective therapeutics, and researchers should also further explore complementary/parallel approaches involving the contribution of APP-CTFs/β to synaptic and neuronal dysfunction during AD onset. This has been included in the discussion section of the revised manuscript.

They should provide a bit more information about Aftin-4 (APA). Most are familiar with beta and gamma-sec inhibitors but the mechanism of action of APA-4 appears complex

and not fully clear. APA was reported to increase Abtea42 but not 40 and even to lower Abeta38.

Author response: There is extensive literature on the effects of Aftin-4 to modulate γ-secretase activity shifting the production to longer forms of Aβ42 species (Bettayeb et al. 2012; Hochard et al. 2013; Meunier et al. 2015; Žnidaršič et al. 2024). Herewith we compare the effect of Aftin-4 treatment on rat primary neurons – ELISA comparing Aβx-40, Aβx-42, Aβx-38, total Aβ and sAPPα/β; in our tested model system. We see a ~2-fold increase in Aβ levels (Fig. 2D-E, Ext. Fig. 2A-E).

It isn't clear from the manuscript what form of Abeta they used when they first use it: was it Abeta1-42? This starts aggregating immediately in solution such as their 100 nM solution and even more in their earlier 230 microM solution; 10 minutes of sonication will not dissolve it all to monomers. Also 100 nM is 1000 fold higher than physiological for extracellular Abeta42, which for the extracellular fluid CSF is 100-250 pM. Thus, their use of the term "monomeric" is not ideal. It is likely a mixture of monomers and various oligomeric species. Running it on a non-denaturing gel would give information about this. Later in the Results they do experiments where they treat separately with 42 and other Abeta peptides, but the precise species the use should be clear throughout.

Author response: Yes, experiments described in the manuscript (main figure 3) were done using Aβ1-42, wherein control experiments used Aβ1-40 and Aβ42-1 (Ext. Fig. 3). This has now been clarified in the text, figures and the respective figure legends.

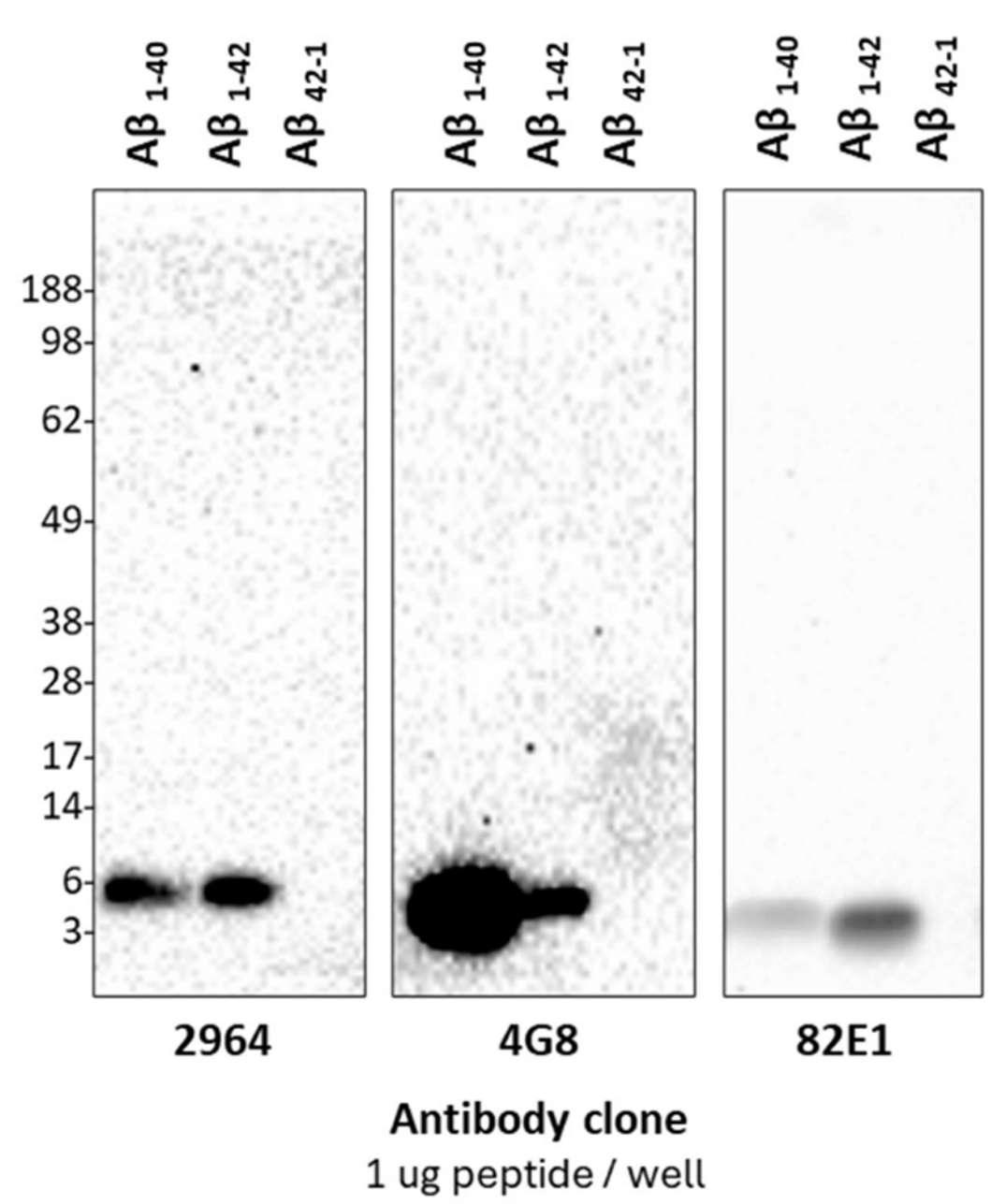

We appreciate this important point about the aggregation propensity of Aβ42 peptides. All Aβ solutions were prepared by solubilizing the lyophilized peptides in NaOH solution, followed by sonication cycles, and flash-freezing; a widely used and optimised protocol to minimize aggregation (Fezoui et al. 2000; Teplow 2006; Taylor et al. 2023; Kumar et al. 2016, 2011; Venegas et al. 2017). We have herewith provided a western blot showing that Aβ peptides prepared using this technique is mostly unaggregated and remains in monomeric form, even when stored in concentrated 230 μM solution. Here we have loaded 1 μl (containing 1μg peptide each) from the prepared 230 μM flash frozen aliquot.

Indeed it is very well known that Aβ42 can spontaneously form oligomers when suspended in cell culture media, thus, keeping this in mind, we designed experiments, wherein the limited exposure time of 5 min reduces/abolishes the chances of oligomer/aggregate formation and/or internalization via endocytosis. This validates that observed effects are via surface interactions and not via triggered intracellular cytotoxic pathways. WB evaluation under non-denaturation conditions has been indicated in the revised manuscript Ext. Fig. 3G. Additionally, while the concentration (100 nm) used exceeded physiological levels, similar concentrations have been previously used in acute synaptic assays to probe rapid functional effects limiting the possibility of overt toxicity (Siddu et al. 2025). These aspects have now been clarified and appropriately referenced in the bibliography.

On line 28 of the Discussion they write "We show that in endogenous conditions, APP and its amyloidogenic processing components are predominantly localized to excitatory presynaptic terminals." Although it is well known that APP is at synapses and traffics there by fast axonal transport, that does not mean most is there. Labeling with an APP CTF specific antibody typically shows most labeling in the area of the Golgi apparatus and in transport and endosomal/autophagic vesicles throughout the cell with eventual degradation in lysosomes. It is also long known that APP processing occurs heavily at synapses where beta- and gamma-secretases have been shown to reside. Remarkably, synaptic activity was shown to promote axonal transport to synapses and generation of Abeta.

Author response: We agree that APP and APP-CTFs exist in two distinct pools, (i) intracellular mostly associated with intracellular organelles i.e. endo-lysosomes, ER and Golgi, and the other (ii) plasma membrane at the synapses. While ADAM10 (**α** secretase) is equally present in sorted synapse subtypes, only β-secretase levels are elevated at the excitatory synapse (see Figure 1); suggesting that amyloidogenic processing of APP is more prominent at the excitatory synapse. This finding is coherent with established literature on APP and its processing at synapses which also strengthen our specific findings (van Oostrum et al. 2023; Fornasiero et al. 2018; Kluever et al. 2022).

They might take a look at a few more recent articles relating to AD ,hyperexcitability and synapses, such as Papanikolaou A et al., 2025, Wilson P, Kim N, et al.2025, Martinsson I et al., 2022, Zarhin D et al., 2022, Zott D et al., 2019, and even Wei W, Nguyen LN et al., 2009, amongst others.

Author response: We thank the reviewer for these references. They have now been included in the discussion section of the manuscript, contextualizing our findings within the growing body of synaptic dysfunction and network hyperexcitability in AD.

Referee #2:

Review report to Kapadia et al. EMBOJ-2026-123845

Summary
This study by Kapadia et al. investigates the synaptic localization of full length APP-FL and its proteolytic products, in particular that of APP-C-terminal fragments (CTFs) to elucidate whether a presynaptic accumulation of CTFs causes presynaptic dysfunction and whether this results in early hyperexcitability in AD.
To this end the authors use fluorescent activated synaptosome sorting from mice with a GFP-knockin into the vGlut1-locus to enrich for synaptosomes from excitatory synapses. They identify in these preparations an enrichment of APP-FL, APP-CTFs as well as all relevant secretases. They investigate the subcellular localization of APP-FL and APP-CTFs and report a selective enrichment of APP-CTFs as compared to APP-FL in presynaptic compartments that is further enhanced by inhibition of amyloidogenic processing. This leads to enhanced synaptic vesicle release probability and thus deficits in vesicle release, as revealed by Ca2+ imaging, SynaptoRed- and Syp-pHflourin imaging in primary neurons. Using immunoisolation the authors go on to show that APP-CTFs associate with the active zone and that this can be enhanced under GSI conditions.

Author response: We thank the reviewer for their careful and constructive evaluation of the manuscript. The comments have helped us improve the scientific rigor and clarity of our work. We have revised the manuscript extensively throughout.

General remarks
The concept that accumulation of APP-CTFs can drive presynaptic dysfunction is not novel. In fact, Barthet et al. 2018 (PMC6235831, see also review by Barthet and Mulle, 2020) previously analyzed presynaptic function in mice with a presynapse-specific KO of PS1/PS2 at Mossy fiber to CA3 synapses. This resulted in impaired presynaptic facilitation and impaired replenishment of the synaptic vesicle pool, which was associated with a drastic reduction in the Ca2+ sensor Synaptotagmin 7. Importantly, it was further shown (using APP-KO/PS1-KO mice) that the depletion of Syt7 was dependent on APP, specificaly the accumulation of APP-CTFs at presynaptic sites. This in vivo study was not quoted nor discussed by the authors. The authors need to address in detail how their study, conducted in vitro, adds conceptually to this previous knowledge. The claim of conceptual novelty that APP-CTF accumulation drives presynaptic dysfunction is therefore not justified.

Author response: We acknowledge that this work was not cited in the original version of our manuscript and have now included it in the discussion section of the revised

manuscript. Our study provides conceptual and mechanistic findings that complement and extend these previous findings. We have therefore revised the manuscript to acknowledge that the accumulation of APP-CTFs/β impair presynaptic function.

In addition, there are major technical limitations (see below) that compromise the conclusions drawn and preclude publication in its present form.

Specific comments:

Point 1: Related to Fig1, but also applies to all other figures. The study essentially relies on antibodies used in WB, ICC, ELISA and proteomics to deduce the specific localization of APP and its fragments at the synapse.

A major concern is therefore the specificity of the Abs for APP-FL and CTFs that are key for the whole study. Here, it is essential to provide experimental evidence, that is currently lacking, that Abs recognize with high specificity and selectivity the claimed fragments and to exclude cross-reactivity with APP-FL (see my comments for example for M3.2 below). Only antibodies directed against a cleavage specific neo-epitope allow to distinguish unambiguously between CTFs and APP-FL. All other antibodies directed at the C-terminus will inevitably also recognize APP-FL, which is particularly relevant in ICC or IHC where the size of Fragments cannot be relvealed.

If stainings with different antibodies are compared, e.g a staining directed against an N-terminal epitope (e.g. polyclonal rabbit SAB4200536) versus a staining using a C-terminal epitope, differences in localization to a certain subcellular structure may result from differences in the affinity and thus sensitivity of the antibodies. Lack of presynaptic staining (or less intense staining) with the N-terminal Ab may result from insufficient sensitivity. This issue needs to be addressed and excluded experimentally.

In each figure panel it must be specifically stated which Ab from the long list was used (e.g. 4 different Ab are directed against the C-terminus).

Author response: First, we would like to clarify that we did not use any proteomics approach in this study because of the impossibility of distinguishing between APP-FL and CTFs - due to total sequence overlap, using such mass spectrometry.
We agree with the reviewer that antibody specificity has always been a critical issue in the field (S. Hunter and Brayne 2017). We have further substantiated the revised manuscript and SI files to improve transparency and experimental support for the usage of antibodies in the different experimental paradigms.

(i) Specific antibody clones used in each experiment have been mentioned now in the figure legend and modified the labels in the figure panels appropriately.
(ii) Tables S2 includes the specific antigen sequences the antibodies target, respectively.
(iii) we have further elaborated on the schematic (previously Fig. S12 now in Fig. SI Fig. S9, to depict APP antibody epitopes and their overlapping reactivities.
(iv) Additional WB validation has been provided herewith to demonstrate antibody behavior.

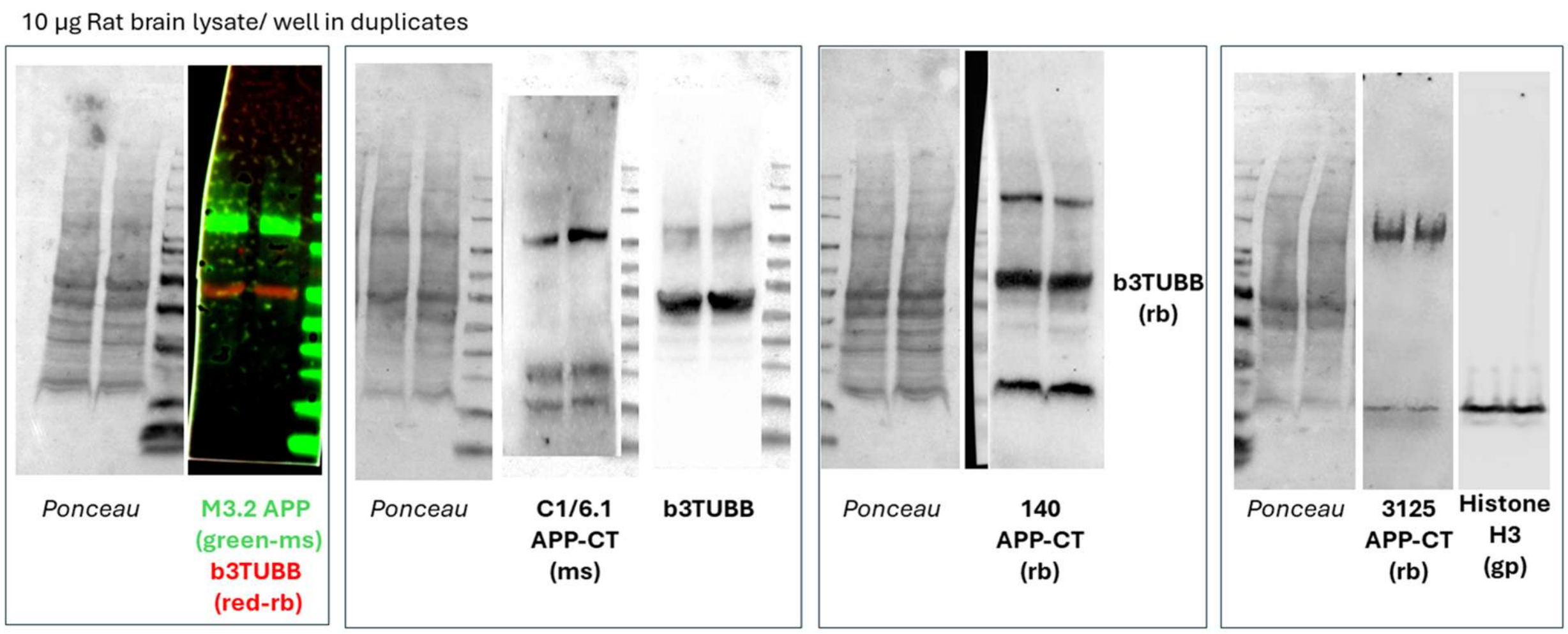

Importantly, we agree that antibodies directed to the APP C-terminus do not, by themselves, unambiguously distinguish APP-FL from APP-CTFs in ICC/IHC due to the sequence similarity of the C-terminal domain. We have revised the manuscript now to include the specific antibody clones in each image/experiment to avoid overinterpretation in cases where APP/APP-CTFs discrimination is not possible by staining alone. Importantly, the revised manuscript now relies on the specific combination of orthogonal approaches:
(i) targeting the N and C-terminus specifically to resolve differences,
(ii) western blotting where we can resolve for APP/APP-CTFs,
(iii) manipulation to enrich for specific APP metabolite within physiological levels,
(iv) Sequential immunoisolation approaches.
Thus, we can say with strength that our conclusions do not single-handedly rely on a detection with a single antibody alone. We have now also toned down the wording in all relevant sections to enable distinction between full length APP, APP-CTFs wherever appropriate.

Moreover, for ICCs negative controls for background staining need to be provided and APP-KO neurons should be used to exclude unspecific signals, that have been notoriously found in the AD field.

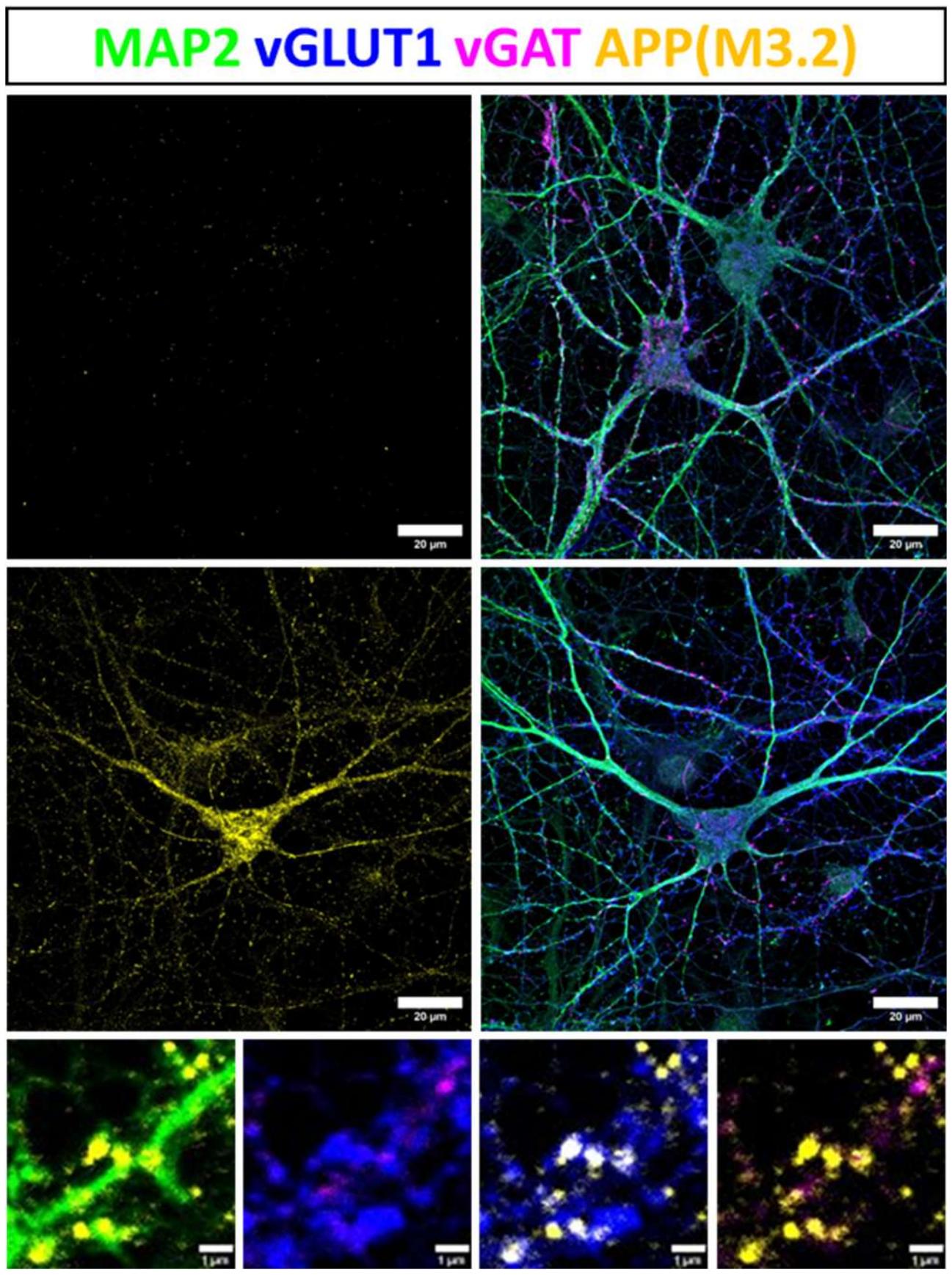

Author response: We have now clarified/added relevant background (secondary antibody) controls in the revised version of the manuscript/SI files wherever required, please see figure below stained without (top) and with APP-M3.2 antibody (bottom) with Goat anti-Mouse IgG A488 (Abcam #ab150113). To further eliminate antibody-based background signals, all secondary antibody combinations were tested in a pilot experiment. Additionally, we interchanged the secondary antibodies in different experimental conditions and detected similar signals across experiments. Please see the list of validated fluorescent conjugated secondary antibodies, mentioned in SI, Table S1.

We agree that while APP-deficient conditions (siRNA based knockdown) is a more stringent specificity control, they were used to only access APP dependence, strengthening the APP-CTFs/β specifically. Our conclusions are strengthened by respective staining conditions, western blot validation, pharmacological modulation and the APP-loss-of-expression control experiments.

In addition, the authors should generate a schematic figure depicting the various APP processing products and antibodies used to distinguish between them or indicate overlapping reactivity.

Author response: We thank the reviewer for this helpful comment. A schematic has been depicted in SI, scheme S1, depicting the antibody clones used in this study, and their expected overlapping reactivity profiles.

In the methods section on page 19 the antibody M3.2 is indicated as APP-C-terminus specific. This is actually not true as the antibody binds between the b- and a-secretase site and will therefore detect both APP-FL and CTFβ. Also, C1/6.1 will detect both APP-FL and CTFs.

Author response: We have corrected the antibody descriptions and added the relevant information in the methods table. We apologize for the typographical error. The correct information was indicated in SI, Table S2, Additionally, the revised antibody schematics and tables now reflect these distinctions more accurately.

Specific comments to Fig 1 O : It is unclear how APP-FL was distinguished from APP-CTFs (CTFa or CTFb?) using ICC on primary neurons, please explain this essential point and provide additional data (see my remarks above). The M3.2 antibody , as well as C1/6.1, is not suited for distinguishing APP-FL from APP-CTFs (see above). Also the details provided in Table S1 and S2 are not sufficient and need to be further validated by showing experimental data for the specificity/epitope of each antibody. This also applies to in house generated antibodies (JW, Eurogentec 3125 and 140-rb).

Author response: We agree that this was insufficiently explained. In the revised version of the manuscript, ICC was not used as a standalone method to distinguish APP-FL from the CTFs (including alpha and beta-species). This was established by western immunoblotting and complementary biochemical assays (epitope specific immunoisolation). The ICC experiments depicted in panel Fig. 1O-P were intended to visualize relative synaptic localization patterns using the epitope-specific antibodies. The revised version of the manuscript includes the descriptions in the respective figure legends, tables and figures. In-house generated antibodies have been previously validated in literature reports published earlier (Wahle et al. 2006; Rijal Upadhaya et al. 2012; Mitroi et al. 2017; Jardanhazi-Kurutz et al. 2010).

Point2, related to Fig 1: The authors state that most vGAT -enriched synapses (GFP) did not contain APP, APP-CTFs, BACE and PSEN. This statement, also made in the abstract, needs to be toned down as panels Fig 1G-N clearly show that these proteins were pretty abundant and detected at about 50% in GFP-negative synapses. There is also evidence from studies using interneuron-specific BACE-KO models that a large proportion of Abeta is generated by interneurons (e.g Rice et al., PMC6950898) and scRNAseq data show the co-expression of APP and of the secretase machinery in interneuron populations.

Author response: This is an important point. We note that the GFP-negative sorted-synapse population should not be interpreted as a pure inhibitory synapse population. Rather, it presents vGLUT1-GFP-depleted material enriched for vGAT-positive synapses, but this likely contains other synaptic populations and a minor fraction of weakly fluorescent excitatory synapse populations below the sorting threshold of the FASs sorter. This has now been explicitly stated in the revised version of the manuscript. Our revised conclusion is that APP, APP-CTFs and other amyloidogenic processing machinery are enriched at the excitatory synapses, rather than being completely absent from inhibitory

or GFP-negative fractions. These findings are also further substantiated by additional results/databases which have specifically isolated synapse-subtype populations using a similar technique (REF}. We have also incorporated additional literature indicating APP-processing in interneurons and other neuronal populations.

Point 3, related to Figure 2: the authors use various secretase inhibitors to modulate the abundance of APP fragments in particular APP-CTFs. In Fig 2F it is again unclear how APP-FL and APP-CTFs were distinguished. Please also specify how the biological replicates were obtained, what means N=4?

Author response: We have now explicitly clarified/stated that ICC-based distinction is interpreted in conjunction to validated WB and biochemical experiments.
Biological and technical replicates have been consistently defined throughout the manuscript. Capital 'N' refers to independent biological replicates; i.e., independent neuronal preparations prepared in different batches. Lowercase 'n' refers to technical replicates or individual measurements within the particular biological replicate. This has now been clarified in the methods section (statistical analyses) of the manuscript.

Point 4, related to Figure S3, panel A-E: ELISA was used to monitor the specificity and efficiency of secretase inhibitors. It appears that the data are not always supporting the expected modulation of the pathways. e.g the ASM (a-secretase inhibitor) did not significantly reduce sAPPa and BSI did not reduce sAPPb. The same is true for WBs of neuronal homogenates (Fig S3G, H): ASM and GSI did not modulate levels of APP-CTFa and BSI did not modulate CTFb, respectively. These pharmacological agents have previously been used by many labs in primary neurons with the expected outcomes, also without enrichment for synaptosomes. The ELISA used is not the most sensitive available in the field (e.g. compared to MSD ELISAs). These shortcomings need to be improved, or the focus should shift to GSI inhibitors only, that showed robust data.

Author response: Our intention in using multiple modulators was to evaluate complementary perturbations of APP processing in rat neuronal cultures, and place primary emphasis on the GSI condition, which yielded the most interesting biochemical and functional effects. The other conditions are presented as supportive, comparative manipulations to validate the findings (in particular, GSI) to validate/eliminate compensatory or off-target consequences. The revised version of the manuscript and the SI file improve the focus and avoids overinterpretation of results.

Point 5, related to Figure 2 G.

The authors assess synapse size from ICC in primary neurons by calculating the vGlut1 positive area. This needs to be substantiated with an independent measure of synapse size.
There is also concern that vGlut1 positive puncta detected in primary neurons do not necessarily represent mature and active synapses. Staining with a postsynaptic marker is needed to substantiate these findings and all conclusions drawn from it.

Author response: We agree with the reviewer that vGLUT1-positive area is not alone sufficient universal proxy for synapse size and maturity of synapse puncta. Thus, the staining with pan-synaptic marker Synapsin1/2 (SI, Fig. S5D) as well as loading of synapses with a synaptic vesicle labelling dye-SynaptoRed (SI, Fig. S5C) was also performed, which show comparable observations with the vGLUT1-based measurements.

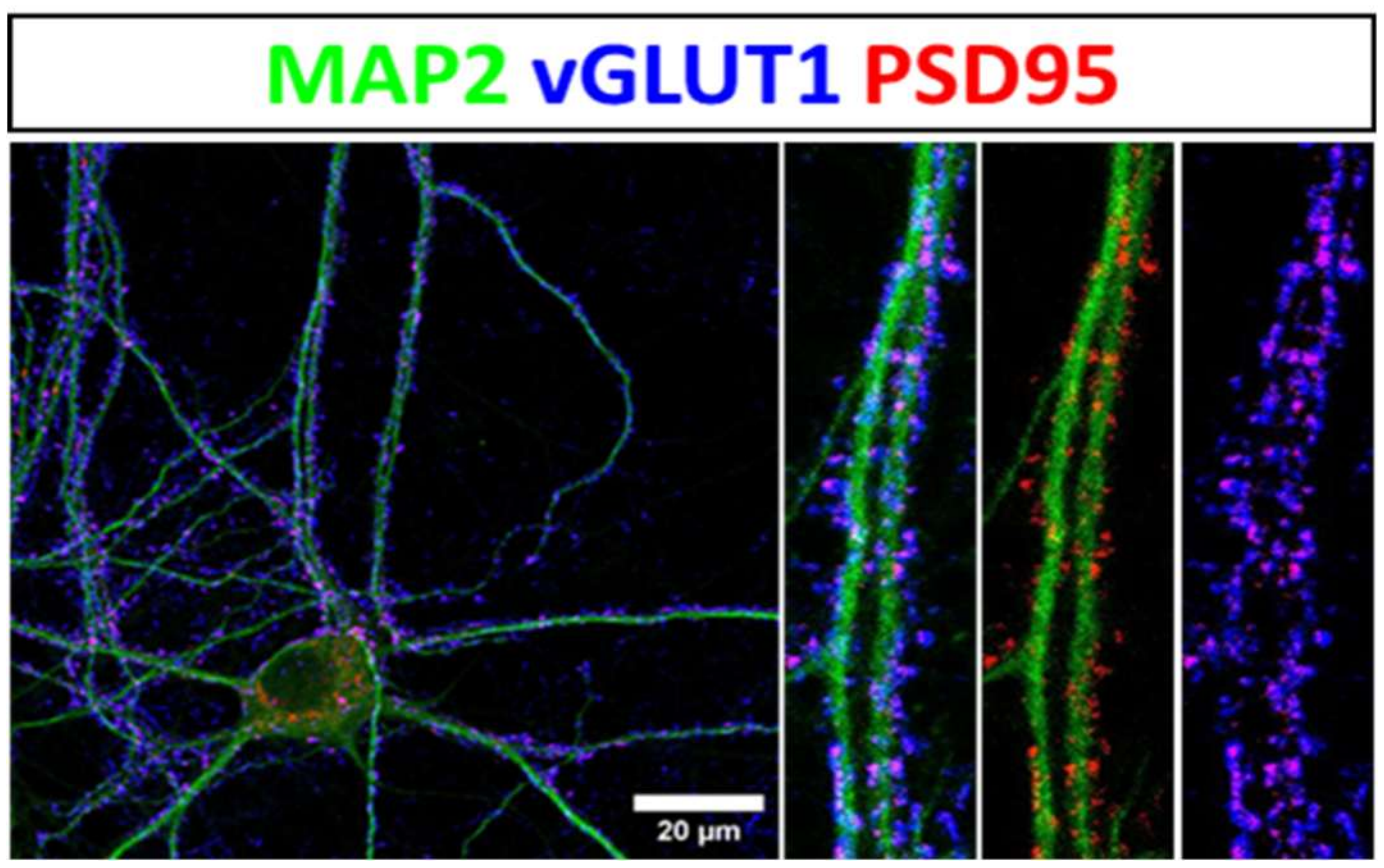

We have additionally presented a representative co-staining with post-synaptic markers to further substantiate that majority (> 40 %) of the synapses analyzed in this study were bonafide synapses in the mature DIV21 neuronal cultures. We have revised the wording to presynaptic bouton area, where appropriate rather than equating that to the synapse size in general. We believe that this will substantially strengthen our interpretation.

Point 6, related to Fig 5A,B. Please describe in more detail the blots depicted in panels A and B , the labelling of the Y axis and how the quantitative evaluation of the data was performed.

Author response: We have revised the figure legends and methods section to provide a more detailed description of the ELISA sandwich strategy. In brief, ELISA plates were first coated with an antibody of interest (capture antibody), e.g. panel A experiments APP-CT antibody or panel B antibody against Sty1, Syb or Stx1. Wells were blocked with BSA to block unreactive sites. These wells were then loaded with synapse homogenates prepared by mechanistical homogenization in an isotonic buffer to maintain protein-protein interactions. Following washing steps, a different detection antibody, in panel A antibody against Syt1, Syb or Stx1 and panel B- APP-CT antibody was used to measure the amount of each respective protein.

Minor points

1) The paper focuses primarily on CTFb, although the physiologically most relevant pathway in wildtype neurons is CTFa. Please comment and explain.

Author response: We agree that in the physiological system, non-amyloidogenic processing and thus the generation of APP-CTFα is highly relevant in wild-type neurons. Our focus on APP-CTFβ stems from the observation that the amyloidogenic machinery is present explicitly at the excitatory synapse. This also has the strongest functional phenotype upon APP-CTFβ accumulation. The discussion aspect of the revised manuscript now addresses these aspects.

2) The discussion is in part highly speculative and rather general (e.g. Page 15, lines 8-17) It would greatly benefit from a clear focus that is more closely related to the data presented.

Author response: The discussion aspect of the manuscript has been rewritten and rephrased to reduce speculative statements and focus more tightly on the data presented in this study with links to prior literature, where necessary.

3) Page 15, line 20: check wording and grammar

Author response: The corresponding sentence has been revised for grammar and clarity.

Referee #3:

- general summary and opinion about the principle significance of the study, its questions and findings

APP, the mother protein of peptide Abeta, is expressed in most of brain cells. Its processing is highly complex, modulated by different consecutive cleavages by different enzymes as BACE, ADAM and gamma-secretase. The authors address a very important questions in the field of neurophysiology and in particular in the context of Alzheimer's disease. Is there a cell type difference in what regards to the expression of APP and APP processing ? If so, will this difference have a functional impact, which could be related to AD pathology if APP processing is changed ? Which APP fragment would be the culprit contributing the most to AD ?

To my knowledge the authors presented convincing data to these questions with elegant technical approaches.

- specific major concerns essential to be addressed to support the conclusions

I don't have major concerns.

Author response: We thank the reviewer for their positive and encouraging evaluation of our work and recognizing the importance and the technical approaches used in this study.

- minor concerns that should be addressed

the study would benefit from an experiement showing a the observed prevalence of APP and BACE expression in excitatory neurons from a human model, thus allowing translation of the observed results. The authors show the increased expression of APP-CTFbeta in AD brains through electronic microscopy, but these data does not convincingly show that APP and amyloidogenic pathway is prevalent in excitatory neurons indetriment to inhibitory neurons.

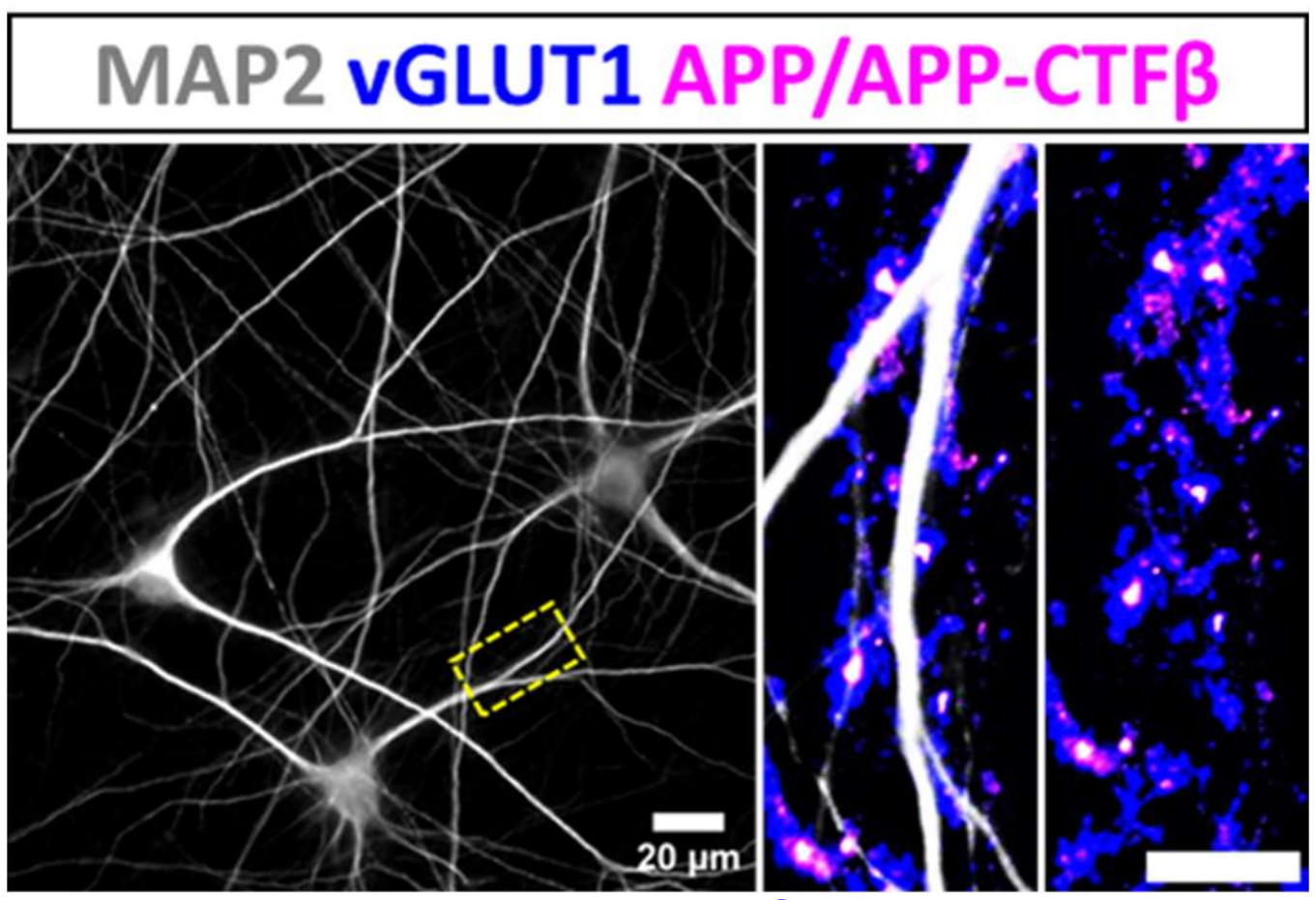

Author response: Emerging transcriptomic and proteomic datasets report detection of APP, BACE1, and γ-secretase components (mRNA and proteins) across neuronal populations (Schanzenbächer et al. 2016; Glock et al. 2021; Fornasiero et al. 2018), with notable enrichment in excitatory neurons (van Oostrum et al. 2023). Previously published literature has indeed shown this in human AD patient samples APP as well as BACE1, localized with synaptic proteins around amyloid plaques (Jordà-Siquier et al. 2022). While we agree that direct demonstration of APP and its amyloidogenic pathway enrichment in excitatory synapses would strengthen the translational relevance of this study. This is currently ongoing wherein we have preliminary data depicting the presence of APP at vGLUT1+ boutons in human iPSC derived excitatory neurons.

iPSC derived excitatory iN (NGN protocol)- DIV49, stained with antibody 6E10 which recognizes human APP, APP-CTFβ and Aβ, respectively.

- any additional non-essential suggestions for improving the study (which will be at the author's/editor's discretion)

The discussion presents several sentences that are quite speculative. I would suggest the authors to restrain the discussion to the data in regard to the literature. Ex. Line 8 to 17, page 15.

Author response: We have now revised/rewritten the discussion to improve focus and precision while reducing speculative statements to closely align the data in this study with existing literature.

List of References:

Bettayeb, Karima, Nassima Oumata, Yuanyuan Zhang, Wenjie Luo, Victor Bustos, Hervé Galons, Paul Greengard, Laurent Meijer, and Marc Flajolet. 2012. “Small-Molecule Inducers of Aβ-42 Peptide Production Share a Common Mechanism of Action.” *FASEB Journal: Official Publication of the Federation of American Societies for Experimental Biology* 26 (12): 5115–23.

Cavallucci, Virve, Marcello D’Amelio, and Francesco Cecconi. 2012. “Aβ Toxicity in Alzheimer’s Disease.” *Molecular Neurobiology* 45 (2): 366–78.

Chundu, Umesh C., Sree Ram Thiriveedhi, Chetanya Bhatti, Jaya Sai Mupparaju, Nina Otinashvili, and Elene Gelishvili. 2025. “A Systematic Review of the Efficacy and Safety of Anti-Amyloid Monoclonal Antibodies in Alzheimer’s Disease.” *Cureus* 17 (6): e85377.

Dyck, Christopher H. van, Chad J. Swanson, Paul Aisen, Randall J. Bateman, Christopher Chen, Michelle Gee, Michio Kanekiyo, et al. 2023. “Lecanemab in Early Alzheimer’s Disease.” *The New England Journal of Medicine* 388 (1): 9–21.

Fezoui, Y., D. M. Hartley, J. D. Harper, R. Khurana, D. M. Walsh, M. M. Condron, D. J. Selkoe, P. T. Lansbury Jr, A. L. Fink, and D. B. Teplow. 2000. “An Improved Method of Preparing the Amyloid Beta-Protein for Fibrillogenesis and Neurotoxicity Experiments.” *Amyloid: The International Journal of Experimental and Clinical Investigation: The Official Journal of the International Society of Amyloidosis* 7 (3): 166–78.

Fornasiero, Eugenio F., Sunit Mandad, Hanna Wildhagen, Mihai Alevra, Burkhard Rammner, Sarva Keihani, Felipe Opazo, et al. 2018. “Precisely Measured Protein Lifetimes in the Mouse Brain Reveal Differences across Tissues and Subcellular Fractions.” *Nature Communications* 9 (1): 4230.

Glock, Caspar, Anne Biever, Georgi Tushev, Belquis Nassim-Assir, Allison Kao, Ina Bartnik, Susanne Tom Dieck, and Erin M. Schuman. 2021. “The Translatome of Neuronal Cell Bodies, Dendrites, and Axons.” *Proceedings of the National Academy of Sciences of the United States of America* 118 (43): e2113929118.

Gouras, Gunnar K., Katarina Willén, and Davide Tampellini. 2012. “Critical Role of Intraneuronal Aβ in Alzheimer’s Disease: Technical Challenges in Studying Intracellular Aβ.” *Life Sciences* 91 (23–24): 1153–58.

Guiloff, A. E., and P. Rudge. 2024. “Amyloid Antibody Therapy for Early-Stage Alzheimer’s Disease: A Critical Review of Three Recent Trials.” *Journal of Neurology* 271 (5): 2914–16.

Hochard, Arnaud, Nassima Oumata, Karima Bettayeb, Olfa Gloulou, Xavier Fant, Emilie Durieu, Nelly Buron, et al. 2013. “Aftins Increase Amyloid-Β42, Lower Amyloid-Β38, and Do Not Alter Amyloid-Β40 Extracellular Production in Vitro: Toward a Chemical Model of Alzheimer’s Disease?” *Journal of Alzheimer’s Disease* 35 (1): 107–20.

Honig, Lawrence S., Marwan N. Sabbagh, Christopher H. van Dyck, Reisa A. Sperling, Steven Hersch, Andre Matta, Luigi Giorgi, et al. 2024. “Updated Safety Results from Phase 3 Lecanemab Study in Early Alzheimer’s Disease.” *Alzheimer’s Research & Therapy* 16 (1): 105.

Hunter, Philip. 2024. "The Controversy around Anti-Amyloid Antibodies for Treating Alzheimer's Disease : The European Medical Agency's Ruling against the Latest Anti-Amyloid Drugs Highlights the Ongoing Debate about Their Safety and Efficacy: The European Medical Agency's Ruling against the Latest Anti-Amyloid Drugs Highlights the Ongoing Debate about Their Safety and Efficacy." *EMBO Reports* 25 (12): 5227–31.

Hunter, Sally, and Carol Brayne. 2017. "Do Anti-Amyloid Beta Protein Antibody Cross Reactivities Confound Alzheimer Disease Research?" *Journal of Negative Results in Biomedicine* 16 (1): 1.

Jardanhazi-Kurutz, Daniel, Markus P. Kummer, Dick Terwel, Kim Vogel, Thomas Dyrks, Andrea Thiele, and Michael T. Heneka. 2010. "Induced LC Degeneration in APP/PS1 Transgenic Mice Accelerates Early Cerebral Amyloidosis and Cognitive Deficits." *Neurochemistry International* 57 (4): 375–82.

Jordà-Siquier, Tomàs, Melina Petrel, Vladimir Kouskoff, Una Smailovic, Fabrice Cordelières, Susanne Frykman, Ulrike Müller, Christophe Mulle, and Gaël Barthet. 2022. "APP Accumulates with Presynaptic Proteins around Amyloid Plaques: A Role for Presynaptic Mechanisms in Alzheimer's Disease?" *Alzheimer's & Dementia: The Journal of the Alzheimer's Association* 18 (11): 2099–2116.

Kluever, Verena, Belisa Russo, Sunit Mandad, Nisha Hemandhar Kumar, Mihai Alevra, Alessandro Ori, Silvio O. Rizzoli, Henning Urlaub, Anja Schneider, and Eugenio F. Fornasiero. 2022. "Protein Lifetimes in Aged Brains Reveal a Proteostatic Adaptation Linking Physiological Aging to Neurodegeneration." *Science Advances* 8 (20): eabn4437.

Kumar, Sathish, Nasrollah Rezaei-Ghaleh, Dick Terwel, Dietmar R. Thal, Mélisande Richard, Michael Hoch, Jessica M. Mc Donald, et al. 2011. "Extracellular Phosphorylation of the Amyloid β-Peptide Promotes Formation of Toxic Aggregates during the Pathogenesis of Alzheimer's Disease: Extracellular Phosphorylation of the Amyloid β-Peptide." *The EMBO Journal* 30 (11): 2255–65.

Kumar, Sathish, Oliver Wirths, Kathrin Stüber, Patrick Wunderlich, Philipp Koch, Sandra Theil, Nasrollah Rezaei-Ghaleh, et al. 2016. "Phosphorylation of the Amyloid β-Peptide at Ser26 Stabilizes Oligomeric Assembly and Increases Neurotoxicity." *Acta Neuropathologica* 131 (4): 525–37.

Lozupone, Madia, Vittorio Dibello, Emanuela Resta, Rodolfo Sardone, Fabio Castellana, Roberta Zupo, Luisa Lampignano, et al. 2025. "Uncertainties in Anti-Amyloid Monoclonal Antibody Therapy for Alzheimer's Disease: The Challenges Ahead." *Expert Review of Neurotherapeutics* 25 (6): 649–59.

Meunier, Johann, Nozha Borjini, Cyril Gillis, Vanessa Villard, and Tangui Maurice. 2015. "Brain Toxicity and Inflammation Induced in Vivo in Mice by the Amyloid-β Forty-Two Inducer Aftin-4, a Roscovitine Derivative." *Journal of Alzheimer's Disease* 44 (2): 507–24.

Mitroi, Daniel N., Indulekha Karunakaran, Markus Gräler, Julie D. Saba, Dan Ehninger, María Dolores Ledesma, and Gerhild van Echten-Deckert. 2017. "SGPL1 (Sphingosine Phosphate Lyase 1) Modulates Neuronal Autophagy via Phosphatidylethanolamine Production." *Autophagy* 13 (5): 885–99.

Oddo, Salvatore, Antonella Caccamo, Ian F. Smith, Kim N. Green, and Frank M. LaFerla. 2006. “A Dynamic Relationship between Intracellular and Extracellular Pools of Abeta.” *The American Journal of Pathology* 168 (1): 184–94.

Oostrum, Marc van, Thomas M. Blok, Stefano L. Giandomenico, Susanne Tom Dieck, Georgi Tushev, Nicole Fürst, Julian D. Langer, and Erin M. Schuman. 2023. “The Proteomic Landscape of Synaptic Diversity across Brain Regions and Cell Types.” *Cell* 186 (24): 5411-5427.e23.

Panza, Francesco, Vittorio Dibello, Rodolfo Sardone, Roberta Zupo, Fabio Castellana, Ivana Leccisotti, Maria Claudia Moretti, et al. 2025. “Successes and Failures: The Latest Advances in the Clinical Development of Amyloid-β-Targeting Monoclonal Antibodies for Treating Alzheimer’s Disease.” *Expert Opinion on Biological Therapy* 25 (3): 275–83.

Rijal Upadhaya, Ajeet, Estibaliz Capetillo-Zarate, Irina Kosterin, Dorothee Abramowski, Sathish Kumar, Haruyasu Yamaguchi, Jochen Walter, Marcus Fändrich, Matthias Staufenbiel, and Dietmar Rudolf Thal. 2012. “Dispersible Amyloid β-Protein Oligomers, Protofibrils, and Fibrils Represent Diffusible but Not Soluble Aggregates: Their Role in Neurodegeneration in Amyloid Precursor Protein (APP) Transgenic Mice.” *Neurobiology of Aging* 33 (11): 2641–60.

Schanzenbächer, Christoph T., Sivakumar Sambandan, Julian D. Langer, and Erin M. Schuman. 2016. “Nascent Proteome Remodeling Following Homeostatic Scaling at Hippocampal Synapses.” *Neuron* 92 (2): 358–71.

Siddu, Alberto, Silvia Natale, Connie H. Wong, Hamidreza Shaye, and Thomas C. Südhof. 2025. “Aggregation Shifts Amyloid-β Peptides from Synaptogenic to Synaptotoxic.” *The Journal of Clinical Investigation* 135 (24). https://doi.org/10.1172/JCI193407.

Skovronsky, D. M., R. W. Doms, and V. M. Lee. 1998. “Detection of a Novel Intraneuronal Pool of Insoluble Amyloid Beta Protein That Accumulates with Time in Culture.” *The Journal of Cell Biology* 141 (4): 1031–39.

Taylor, Alexander I. P., Peter J. Davis, Liam D. Aubrey, Joshua B. R. White, Zoe N. Parton, and Rosemary A. Staniforth. 2023. “Simple, Reliable Protocol for High-Yield Solubilization of Seedless Amyloid-β Monomer.” *ACS Chemical Neuroscience* 14 (1): 53–71.

Teplow, David B. 2006. “Preparation of Amyloid Beta-Protein for Structural and Functional Studies.” *Methods in Enzymology* 413: 20–33.

Venegas, Carmen, Sathish Kumar, Bernardo S. Franklin, Tobias Dierkes, Rebecca Brinkschulte, Dario Tejera, Ana Vieira-Saecker, et al. 2017. “Microglia-Derived ASC Specks Cross-Seed Amyloid-β in Alzheimer’s Disease.” *Nature* 552 (7685): 355–61.

Wahle, Tina, Dietmar R. Thal, Magdalena Sastre, Andrea Rentmeister, Nenad Bogdanovic, Michael Famulok, Michael T. Heneka, and Jochen Walter. 2006. “GGA1 Is Expressed in the Human Brain and Affects the Generation of Amyloid Beta-Peptide.” *The Journal of Neuroscience: The Official Journal of the Society for Neuroscience* 26 (49): 12838–46.

Wilson, C. A., R. W. Doms, and V. M. Lee. 1999. “Intracellular APP Processing and A Beta Production in Alzheimer Disease.” *Journal of Neuropathology and Experimental Neurology* 58 (8): 787–94.

Žnidaršič, Neža, Neža Grgurevič, Alenka Nemec Svete, Anže Meden, and Tomaž Snoj. 2024. “A Comparison of Cognitive Decline in Aged Mice and Mice Treated with Aftin-4.” *Scientific Reports* 14 (1): 28320.

1st Revision - Editorial Decision **3rd Jul 2026**

Dear Dr. Hafner,

Thank you for the submission of your revised manuscript (EMBOJ-2026-123845R) to The EMBO Journal for our consideration, and for your patience during re-review. Your manuscript and your point-by-point responses to the previous comments of the referees have now been seen by them, and we have received their new reports, which you can find appended below.

I am pleased to say that, as you will see, the referees are now, overall, largely supportive. All three referees recognize that the revised manuscript is considerably improved, and that most of the initially raised concerns have been adequately addressed in the new version of the manuscript that provides a more balanced and well-supported interpretation and discussion of the results. Referees #1 and #2 have only a few remaining points regarding some comments from the previous review round and your responses to them, which should be easily addressable and must be clarified in a final version of the manuscript before we can move forward with the acceptance of the article for publication in The EMBO Journal. Please make sure that the referees' points are fully addressed both in the revised manuscript and in a detailed point-by-point response to the new reports.

From the editorial side, there are also a few changes and corrections we need you to make in the final version of the manuscript, before we can move forward with the next steps towards its publication:

- Please include funding information in the Acknowledgements section of your revised manuscript.

- The same funding information must also be entered in our online system; currently, the following information is missing from the system: "Independent Research Fund Denmark grant 10.46540/2064-00032B; Dr.phil. Ragna Rask-Nielsens Grundforskningsfond".

- Please change heading "References and Notes" to "References".

- All references listed in your point-by-point responses, which will be part of the Peer Review File that is published alongside the article (please see below for more information), must also be included in the main list of References in the manuscript.

- Please change heading "Data and materials availability" to "Data availability".

- All data and code must be publicly available at the time of publication.

- Please change heading "Competing interests" to "Disclosure and competing interests statement".

- The Author Contributions statement should be removed from the manuscript file. Instead, we use CRediT to specify the contributions of each author in the journal submission system. Please feel free to use the free text box to provide more detailed descriptions during submission. See also our guide to authors for more information: https://link.springer.com/partners/embo-press/editorial-policies#Authorship.

- Figure panel callouts should be listed in the manuscript sequentially.

- We noticed that callouts for Fig. 3P, 7C-D, Figures S1 to S15, and Tables S3-S6 are missing.

- There is a callout for Fig. 7J-K, but there are no panels J and K in Fig. 7.

- EV Figure legends in the manuscript must be renamed to "Figure EV1-EV4" instead of "Extended Figure 1-4".

- EV Figure callouts should be renamed to "Figure EV1-EV4" instead of "Ext. fig. 1-4".

- Source file names, titles, legends and manuscript callouts need to be updated to "Dataset EV1-EV#" instead of "Supp_data_file"; their legends should be uploaded in a separate tab/sheet in each Excel file.

- The Appendix file needs to be in PDF format; its title page should contain heading "Appendix for", followed by the manuscript's title (author and affiliation lists are not necessary), and a Table of Contents including page numbers for all listed items; nomenclature should be "Appendix Figure S#" and "Appendix Table S#" throughout the main manuscript file and the Appendix PDF file.

- Please note that EMBO press papers are accompanied online by:
A) a short (2 sentences) summary of the findings and their significance,
B) 2-5 short bullet points highlighting the key results, and
C) a synopsis image in .jpg or .png format that is exactly 550 pixels wide and 300 pixels high. Please note that all text needs to be legible at the final size.

Please upload this information along with your revised manuscript (the text for A and B should be provided in a separate Word file).

- During our standard Figure integrity checks, our team detected a case of possible blot reuse between Figure S1H and S7B; the blot appears to be horizontally flipped. This issue must be clarified and corrected before we can move forward with the acceptance of the article for publication here. Please check these Figures carefully, correct them if necessary, clarify in your cover letter, and include in your Source Data the original uncropped, unprocessed blots for these Figures. Please note that if reuse is intentional and scientifically justified, it must be stated in the legends.

- Our data editors have checked your Figures and their legends, and raised the following queries. Please address all points below completely in your revised manuscript (all changes should be highlighted or "tracked"):
1. Please note that the box plots need to be defined in terms of minima, maxima, centre, bounds of box and whiskers, and percentile in the legends of Figures 2G,H,J; 7E-H.
2. Please note that information related to "n" is missing in the legends of Figures 7E-H.
3. Please note that n=2 in Extended Figure 3C,E; Extended Figure 4D. In such cases, the individual data points must be shown, and no statistics can be calculated and shown.
4. Please define the error bars in the legends of Figures 1D,E,G,H,J,K,M,N; 2B-E; 3G,H,L,O; 5A,B; 6H; 7B; Extended Figure 1A-M,O; Extended Figure 2A-I,K,M-O.
5. Please provide the exact p-values in the legends of Figures 1D,E,G,H,J,M,N; 2B-E,G,H,J; 3G,H,L,O; 6H; 7B,F,G,H,I; Extended Figure 1O; Extended Figure 2A-I,K,N.
6. Please define the scale bar for Figures Extended Figure 3F; Extended Figure 4B.

- "Short summary" should be removed from the revised manuscript.

- "Ethical approval" should be included in the Methods text, not provided as a separate section.

- Movie files should be renamed to "Movie EV1-EV6" and their corresponding callouts updated accordingly; their legends should be removed from the Appendix PDF file and instead zipped together with each Movie file.

- Manuscript sections need to be named and ordered as follows: Title page - Abstract - Introduction - Results - Discussion - Methods - Data Availability - Acknowledgements - Disclosure and Competing Interests Statement - References - Figure Legends - main Tables (if there are any) - Expanded View Figure Legends (if there are any).

- Please also note that as part of the EMBO Press transparent editorial process, The EMBO Journal publishes online a Peer Review File along with each accepted manuscript. This File will be published in conjunction with your paper and will include the referee reports, your point-by-point responses and all pertinent correspondence relating to the manuscript. Your Author's Checklist will also be published at the end of the Peer Review File. Please let us know in case you want to remove any data or figures from your point-by-point responses before they are published as part of the Peer Review File. Retaining unpublished data in the Peer Review File means that these count as published and that the Peer Review File would need to be referenced in future publications. Please let the editorial office know in case you want to remove any data from this file (contact@embojournal.org).

We look forward to seeing a final version of your manuscript as soon as possible.

Best regards,

Ioannis

Ioannis Papaioannou, PhD
Editor, The EMBO Journal
i.papaioannou@embojournal.org

Referee #1:

This is an excellent and improved manuscript revision.
I have 2 minor points, one about a response to one of my prior review points and one about a minor point raised by reviewer 2.

1. They write: "We would also like to point out that the intracellular pools of Aβ could also be in some part APP-CTFβ species which are cross-detected using antibodies that could indirectly detect other species (S. Hunter and Brayne 2017)."
In response: Absolutely, and as S. Hunter and Brayne 2017 describe, there was a problem with a lack of understanding among quite a few AD researchers about the use of standard Abeta antibodies that cross-react with APP. However, this is much less of an issue since the generation of Abeta end-specific antibodies. The majority of articles that have worked on intraneuronal Abeta have been aware of these antibody epitope issues. There are a few outliers, such as their reference to Oddo et al., 2006, who did use such an Abeta/APP antibody for intraneuronal Abeta; that paper reasoned that it must be Abeta since they did not see similar labelling with a C-terminal APP antibody. While Hunter & Brayne focus on Abeta/APP antibodies, they do also mention that C-terminal antibodies might also be detecting the gamma-secretase product of the alpha-secretase pathway, p3. Although the topic of diverse N-terminal truncation of Abeta is important and well discussed by Hunter & Brayne, biochemical evidence (e.g. mass spec) has not detected appreciable levels of alpha-secretase generated p3 in brain (e.g. Portelius E et al., Acta Neuropathol. 2010).
2. Reviewer 2's minor point 1: The paper focuses primarily on CTFb, although the physiologically most relevant pathway in wildtype neurons is CTFa. Please comment and explain"
My comment: Alpha-secretase is not the major secretase for neurons as it is for non-neuronal cells. As shown already by the Beyreuther lab in Heidelberg three decades ago, neurons have an alternate beta-cleavage site that some have confused as alpha-cleavage p3 since it doesn't separate well from it on Western blots (Simons M et al., 1996 and Tienari PJ et al., 1997); see also the initial BACE! paper by Vassar R et al., 2000 showing that this alternate Abeta is from an alternate BACE1 beta cleavage site. Or see again Portelius E et al., 2010 noted in the prior point for human brain.

Referee #2:

The revised manuscript is considerably improved, the authors now provided:
- a scheme depicting the epitopes of all the antibodies used (Fig S1)
- a more detailed table indicating the specific use of all antibodies (Table S1)
- a table of antibodies against APP, APP-CTFs and Ab (Table S2)
- indicate in each of the figure legends which antibody was used
- now indicate that the antibody M3.2, recognizes both APP-FL and CTFs
- an improved and balanced discussion with a clear focus to data presented in their study

However, there is still a major technical concern and misinterpretation of the data that was not adequately addressed and needs to be corrected. The story as a whole is very interesting and this remaining issue can be easily addressed.

This refers to the APP species and fragments that are detected by the antibody C1/6.1.
This antibody is directed against the APP C-terminus (aa 676-695). The antibody was originally developed by the lab of Paul Matthews and R. Nixon (see e.g. Mathews et al. J Biol Chem 2002 Vol. 277 Issue 39 Pages 36415-24; Morales-Corraliza et al. PLoS One 2009 Vol. 4 Issue 9 Pages e7134), is now commercially available (e.g. by Biolegend https://www.biolegend.com/de-de/products/purified-anti-app-c-terminal-fragment-antibody-11275?GroupID=BLG15648) and has since been widely used by many labs including our own.

From the literature and the data sheet of the company it is evident that C1/6.1 detects both APP-FL and CTFs. Despite this, the authors write in table S2 that the Ab is specific to APP-CTFs and shows only mild crossreactivity to APP-FL.
Accordingly, they write in the manuscript that species detected by C1/6.1 correspond to APP-CTFs, which is highly misleading when using immunocytochemistry or any other technique that falls short of resolving the size.

See for example:
Legend to Fig1 L: „(O-P) Immunocytochemistry and Imaris 3D reconstruction of rat primary cortical neurons (DIV21) depicting localization of APP-NT (clone: M3.2, N, grayscale, yellow-merge); BACE1 (N, magenta) and APP-CTFs (clone: C1/6.1, O, grayscale, yellow-merge)"

Legend Fig2: „(F) ICC depicting relative changes in the levels of APP-CTFs upon treatment with different compounds (clone: C1/6.1, gray, yellow-merge). Neurons were also co-stained with vGLUT1 (excitatory synapses, blue), vGAT (inhibitory synapses, magenta) and MAP2 (dendrites, green). Box selection depicts the zoomed in panel. Arrows depict colocalization of APP-CTFs with vGLUT1+ synapse. „

Legend Fig4: „(B) Immunocytochemical analysis depicting colocalization between APP-CTFs (clone: C1/6.1) and presynaptic active zone protein RIM1"

This criticism also applies to the manuscript text referring to these experiments.

The WB provided in the response letter is not resolving this issue: for all blots the size of the marker proteins are missing, nor are the bands detected labelled. While two smaller band are detected with C1/6.1, that might correspond to CTFs, there is also a prominent band of higher mobility that might correspond to APP-FL (or an unspecific band?). Failure to detect bands corresponding to APP-FL may have many reasons, including insufficient transfer during blotting.

Anyway, given the vast amount of literature from independent laboratories using this antibody the claim that APP-FL is not detected by C1/6.1 cannot be upheld.
This could be easily avoided by changing the wording to APP-CT immunoreactivity.

Apart from this issue I agree with the authors that they provide several independent lines of evidence, in particular their data using GSI inhibitors, that link an increase in CTFs (WB) to an increase in presynaptic accumulation of APP-C-terminal immunoreactivity that is associated with the presynaptic deficits. The story as a whole is very interesting and there is no need to claim that CTFs can be detected by antibodies such as C1/6.1.

Minor comments:
The authors may not to be aware that the Ab 82E1 (used e.g. in Fig 7) does indeed detect CTFs (and Ab) as it is directed to the very N-terminus of CTFb that is generated upon BACE cleavage. This should be mentioned in the table and manuscript. This is the only antibody that detects a cleavage-specific neo-epitope, while all other Abs directed against the C-terminus also detect APP-FL.

I also had a look at the source data supporting the WB quantification of the Ext. Fig.2J:
The WBs given in the source data do not correspond to the WB used for the quantification. The source data show the optimization of inhibitors, e.g. slide 1: 1µM DAPT, 10µM DAPT. However, the final WBs used for the quantification were lacking, only prism files of the calculation were provided.
In addition, it is obvious from the Ponceau staining that protein transfer was quite unequal. How was this taken into account for quantification?

It would be helpful to add references to the tables for the in-house generated Ab that are not commercially available.

Referee #3:

The authors have satisfactorily addressed the points raised during the review process. The revised Discussion is more balanced and remains closely supported by the experimental data and the existing literature.
The authors provide relevant evidence from published transcriptomic and proteomic studies supporting enrichment of APP processing components in excitatory neurons, appropriately acknowledge this limitation, and present preliminary observations in human iPSC-derived excitatory neurons that are consistent with their conclusions. Although these experiments would add value, they are not required to support the central conclusions of the manuscript.
Overall, the revised manuscript provides a rigorous and comprehensive analysis of cell type-specific APP processing and its functional implications, and I consider the conclusions to be well supported by the data presented.

Response to Reviewer comments_R2:

Author response: We greatly appreciate the constructive feedback from all three referees and the editorial team. We thank them very much for their detailed comments and for guiding us through the review and editorial process. All changes have been indicated in red font in the respective documents.

Referee #1:

This is an excellent and improved manuscript revision. I have 2 minor points, one about a response to one of my prior review points and one about a minor point raised by reviewer 2.

1. They write: "We would also like to point out that the intracellular pools of Aβ could also be in some part APP-CTFβ species which are cross-detected using antibodies that could indirectly detect other species (S. Hunter and Brayne 2017)."

In response: Absolutely, and as S. Hunter and Brayne 2017 describe, there was a problem with a lack of understanding among quite a few AD researchers about the use of standard Abeta antibodies that cross-react with APP. However, this is much less of an issue since the generation of Abeta end-specific antibodies. The majority of articles that have worked on intraneuronal Abeta have been aware of these antibody epitope issues. There are a few outliers, such as their reference to Oddo et al., 2006, who did use such an Abeta/APP antibody for intraneuronal Abeta; that paper reasoned that it must be Abeta since they did not see similar labelling with a C-terminal APP antibody. While Hunter & Brayne focus on Abeta/APP antibodies, they do also mention that C-terminal antibodies might also be detecting the gamma-secretase product of the alpha-secretase pathway, p3. Although the topic of diverse N-terminal truncation of Abeta is important and well discussed by Hunter & Brayne, biochemical evidence (e.g. mass spec) has not detected appreciable levels of alpha-secretase generated p3 in brain (e.g. Portelius E et al., Acta Neuropathol. 2010).

Author response: We have revised the Discussion to more accurately reflect the current understanding of antibody specificity between APP-FL, APP-CTFs/β and intracellular Aβ species. The relevant section has been updated accordingly.

2. Reviewer 2's minor point 1: The paper focuses primarily on CTFb, although the physiologically most relevant pathway in wildtype neurons is CTFa. Please comment and explain"
My comment: Alpha-secretase is not the major secretase for neurons as it is for non-neuronal cells. As shown already by the Beyreuther lab in Heidelberg three decades ago, neurons have an alternate beta-cleavage site that some have confused as alpha-cleavage p3 since it doesn't separate well from it on Western blots (Simons M et al., 1996 and Tienari PJ et al., 1997); see also the initial BACE! paper by Vassar R et al., 2000 showing that this alternate Abeta is from an alternate BACE1 beta cleavage site. Or see again Portelius E et al., 2010 noted in the prior point for human brain.

Author response: We thank the reviewer for this important clarification. We have added a short paragraph to the Discussion to highlight this aspect for the benefit of the readers.

Referee #2:

The revised manuscript is considerably improved, the authors now provided:
- a scheme depicting the epitopes of all the antibodies used (Fig S1)

- a more detailed table indicating the specific use of all antibodies (Table S1)
- a table of antibodies against APP, APP-CTFs and Ab (Table S2)
- indicate in each of the figure legends which antibody was used
- now indicate that the antibody M3.2, recognizes both APP-FL and CTFs
- an improved and balanced discussion with a clear focus to data presented in their study

However, there is still a major technical concern and misinterpretation of the data that was not adequately addressed and needs to be corrected. The story as a whole is very interesting and this remaining issue can be easily addressed.

This refers to the APP species and fragments that are detected by the antibody C1/6.1. This antibody is directed against the APP C-terminus (aa 676-695). The antibody was originally developed by the lab of Paul Matthews and R. Nixon (see e.g. Mathews et al. J Biol Chem 2002 Vol. 277 Issue 39 Pages 36415-24; Morales-Corraliza et al. PLoS One 2009 Vol. 4 Issue 9 Pages e7134), is now commercially available (e.g. by Biolegend https://www.biolegend.com/de-de/products/purified-anti-app-c-terminal-fragment-antibody-11275?GroupID=BLG15648) and has since been widely used by many labs including our own.

From the literature and the data sheet of the company it is evident that C1/6.1 detects both APP-FL and CTFs. Despite this, the authors write in table S2 that the Ab is specific to APP-CTFs and shows only mild crossreactivity to APP-FL.

Accordingly, they write in the manuscript that species detected by C1/6.1 correspond to APP-CTFs, which is highly misleading when using immunocytochemistry or any other technique that falls short of resolving the size.

See for example:

-Legend to Fig1 L: „(O-P) Immunocytochemistry and Imaris 3D reconstruction of rat primary cortical neurons (DIV21) depicting localization of APP-NT (clone: M3.2, N, grayscale, yellow-merge); BACE1 (N, magenta) and APP-CTFs (clone: C1/6.1, O, grayscale, yellow-merge)"
-Legend Fig2: „(F) ICC depicting relative changes in the levels of APP-CTFs upon treatment with different compounds (clone: C1/6.1, gray, yellow-merge). Neurons were also co-stained with vGLUT1 (excitatory synapses, blue), vGAT (inhibitory synapses, magenta) and MAP2 (dendrites, green). Box selection depicts the zoomed in panel. Arrows depict colocalization of APP-CTFs with vGLUT1+ synapse.

-Legend Fig4: „(B) Immunocytochemical analysis depicting colocalization between APP-CTFs (clone: C1/6.1) and presynaptic active zone protein RIM1"

This criticism also applies to the manuscript text referring to these experiments.
-The WB provided in the response letter is not resolving this issue: for all blots the size of the marker proteins are missing, nor are the bands detected labelled. While two smaller band are detected with C1/6.1, that might correspond to CTFs, there is also a prominent band of higher mobility that might correspond to APP-FL (or an unspecific band?). Failure to detect bands corresponding to APP-FL may have many reasons, including insufficient transfer during blotting.

Anyway, given the vast amount of literature from independent laboratories using this antibody the claim that APP-FL is not detected by C1/6.1 cannot be upheld.

This could be easily avoided by changing the wording to APP-CT immunoreactivity.

Apart from this issue I agree with the authors that they provide several independent lines of evidence, in particular their data using GSI inhibitors, that link an increase in CTFs (WB) to an increase in presynaptic accumulation of APP-C-terminal immunoreactivity that is associated with the presynaptic deficits. The story as a whole is very interesting and there is no need to claim that CTFs can be detected by antibodies such as C1/6.1.

Author response: We thank the reviewer for this important and constructive comment. We fully agree with the reviewer and have revised the text and the figure legends in the manuscript and the SI files accordingly. Throughout the manuscript, we now refer to the C1/6.1 signal as APP C-terminal (APP-CT) immunoreactivity or APP-C-terminal species, rather than APP-CTFs in particular. We have also corrected the description of antibody C1/6.1 in Table S2 to accurately reflect its specificity for both APP-FL and APP C-terminal fragments.

Minor comments:

The authors may not to be aware that the Ab 82E1 (used e.g. in Fig 7) does indeed detect CTFs (and Ab) as it is directed to the very N-terminus of CTFb that is generated upon BACE cleavage. This should be mentioned in the table and manuscript. This is the only antibody that detects a cleavage-specific neo-epitope, while all other Abs directed against the C-terminus also detect APP-FL.

Author response: In Fig. 7 D, we have specifically indicated that antibody clone 82E1 detects APP-CTFβ and Aβ species (magenta). In addition, the description in Table S2 has been revised to explicitly state that 82E1 recognizes the conserved N-terminal neo-epitope present in both APP-CTFβ and Aβ species.

I also had a look at the source data supporting the WB quantification of the Ext. Fig.2J: The WBs given in the source data do not correspond to the WB used for the quantification. The source data show the optimization of inhibitors, e.g. slide 1: 1µM DAPT, 10µM DAPT. However, the final WBs used for the quantification were lacking, only prism files of the calculation were provided. In addition, it is obvious from the Ponceau staining that protein transfer was quite unequal. How was this taken into account for quantification?

Author response: We apologize for the oversight. The complete source data, including the western blots used for quantification, have now been provided. For all western blot quantifications, band intensities were first normalized to the corresponding Ponceau staining or β3-tubulin loading control, as appropriate. The normalized values were then expressed relative to the untreated control condition and are presented as fold change throughout the manuscript, respectively.

It would be helpful to add references to the tables for the in-house generated Ab that are not commercially available.

Author response: We thank the reviewer for this suggestion. The relevant references for the in-house generated antibodies have now been included in Table S1 of the Supplementary Information.

Referee #3:

The authors have satisfactorily addressed the points raised during the review process. The revised Discussion is more balanced and remains closely supported by the experimental data and the existing literature.

The authors provide relevant evidence from published transcriptomic and proteomic studies supporting enrichment of APP processing components in excitatory neurons, appropriately acknowledge this limitation, and present preliminary observations in human iPSC-derived excitatory neurons that are consistent with their conclusions. Although these experiments would add value, they are not required to support the central conclusions of the manuscript. Overall, the revised manuscript provides a rigorous and comprehensive analysis of cell type-specific APP processing and its functional implications, and I consider the conclusions to be well supported by the data presented.

Author response: We sincerely thank the reviewer for the positive evaluation of our revised manuscript and for the encouraging comments.

2nd Revision - Editorial Decision 23rd Jul 2026

Dear Dr. Hafner,

Congratulations on an excellent manuscript! I am very pleased to inform you that it has been accepted for publication in The EMBO Journal. Thank you for comprehensively addressing the initially raised referees' concerns and our editorial requests for changes and corrections.

Your manuscript will be processed for publication by EMBO Press. It will be copy edited and you will receive page proofs prior to publication. Please note that you will be contacted by Springer Nature Author Services to complete licensing and payment information.

You may qualify for financial assistance for your publication charges - either via a Springer Nature fully open access agreement or an EMBO initiative. Check your eligibility: https://link.springer.com/journal/44318/how-to-publish-with-us

Should you be planning a Press Release on your article, please notify the production team via this form in order to coordinate publication and release dates: https://forms.cloud.microsoft/e/eH4A8JVP6g

If you have any questions, please do not hesitate to contact the Editorial Office. Thank you for your contribution to The EMBO Journal. Working with you has been a pleasure.

Best regards,

Ioannis

Ioannis Papaioannou, PhD
Senior Editor, The EMBO Journal - EMBO Reports - Molecular Systems Biology
i.papaioannou@embojournal.org

*****
Please note that it is The EMBO Journal policy for the transcript of the editorial process (containing referee reports and your response letters) to be published as an online supplement to each paper. If you should prefer removal of any referee-only figures included in the point-by-point response(s), e.g. because they may still be used for future publication or because they have been reproduced from published work by others, please do let us know immediately via response email.
More information is available here: https://link.springer.com/partners/embo-press/editorial-policies#Peer%20review

EMBO Press Author Checklist

Corresponding Author Name: Anne-Sophie Hafner	
Journal Submitted to: EMBO J.	
Manuscript Number: EMBOJ-2026-123845R	

USEFUL LINKS FOR COMPLETING THIS FORM
The EMBO Journal - Author Guidelines
EMBO Reports - Author Guidelines
Molecular Systems Biology - Author Guidelines
EMBO Molecular Medicine - Author Guidelines

Reporting Checklist for Life Science Articles (updated January
This checklist is adapted from Materials Design Analysis Reporting (MDAR) Checklist for Authors. MDAR establishes a minimum set of requirements in transparent reporting in the life sciences (see Statement of Task: 10.31222/osf.io/9sm4x). Please follow the journal's guidelines in preparing your
Please note that a copy of this checklist will be published alongside your article.

Abridged guidelines for figures
1. Data
The data shown in figures should satisfy the following conditions:
- the data were obtained and processed according to the field's best practice and are presented to reflect the results of the experiments in an accurate and unbiased manner.
- ideally, figure panels should include only measurements that are directly comparable to each other and obtained with the same assay.
- plots include clearly labeled error bars for independent experiments and sample sizes. Unless justified, error bars should not be shown for technical
- if n<5, the individual data points from each experiment should be plotted. Any statistical test employed should be justified.
- Source Data should be included to report the data underlying figures according to the guidelines set out in the authorship guidelines on Data

2. Captions
Each figure caption should contain the following information, for each panel where they are relevant:
- a specification of the experimental system investigated (eg cell line, species name).
- the assay(s) and method(s) used to carry out the reported observations and measurements.
- an explicit mention of the biological and chemical entity(ies) that are being measured.
- an explicit mention of the biological and chemical entity(ies) that are altered/varied/perturbed in a controlled manner.
- the exact sample size (n) for each experimental group/condition, given as a number, not a range;
- a description of the sample collection allowing the reader to understand whether the samples represent technical or biological replicates (including how many animals, litters, cultures, etc.).
- a statement of how many times the experiment shown was independently replicated in the laboratory.
- definitions of statistical methods and measures:
  - common tests, such as t-test (please specify whether paired vs. unpaired), simple χ2 tests, Wilcoxon and Mann-Whitney tests, can be unambiguously identified by name only, but more complex techniques should be described in the methods section;
  - are tests one-sided or two-sided?
  - are there adjustments for multiple comparisons?
  - exact statistical test results, e.g., P values = x but not P values < x;
  - definition of 'center values' as median or average;
  - definition of error bars as s.d. or s.e.m.

Please complete ALL of the questions below.
Select "Not Applicable" only when the requested information is not relevant for your study.

Materials

Newly Created Materials	Information included in the manuscript?	In which section is the information available? (Reagents and Tools Table, Materials and Methods, Figures, Data Availability Section)
New materials and reagents need to be available; do any restrictions apply?	Not Applicable	

Antibodies	Information included in the manuscript?	In which section is the information available? (Reagents and Tools Table, Materials and Methods, Figures, Data Availability Section)
For **antibodies** provide the following information:<br>- Commercial antibodies: RRID (if possible) or supplier name, catalogue number and or/clone number<br>- Non-commercial: RRID or citation	Yes	Reagents table, Material and Methods, Figure legends

DNA and RNA sequences	Information included in the manuscript?	In which section is the information available? (Reagents and Tools Table, Materials and Methods, Figures, Data Availability Section)
**Short novel DNA or RNA including primers, probes**: provide the sequences.	Not Applicable	

Cell materials	Information included in the manuscript?	In which section is the information available? (Reagents and Tools Table, Materials and Methods, Figures, Data Availability Section)
**Cell lines:** Provide species information, strain. Provide accession number in repository **OR** supplier name, catalog number, clone number, and/**OR** RRID.	Yes	Material and methods
**Primary cultures:** Provide species, strain, sex of origin, genetic modification status.	Yes	Material and methods
Report if the cell lines were recently **authenticated** (e.g., by STR profiling) and tested for mycoplasma contamination.	Not Applicable	

Experimental animals	Information included in the manuscript?	In which section is the information available? (Reagents and Tools Table, Materials and Methods, Figures, Data Availability Section)
**Laboratory animals or Model organisms:** Provide species, strain, sex, age, genetic modification status. Provide accession number in repository **OR** supplier name, catalog number, clone number, **OR** RRID.	Yes	Reagents table, Material and Methods
**Animal observed in or captured from the field:** Provide species, sex, and age where possible.	Not Applicable	
Please detail **housing and husbandry conditions**.	Yes	Material and Methods

Plants and microbes	Information included in the manuscript?	In which section is the information available? (Reagents and Tools Table, Materials and Methods, Figures, Data Availability Section)
**Plants:** provide species and strain, ecotype and cultivar where relevant, unique accession number if available, and source (including location for collected wild specimens).	Not Applicable	
**Microbes:** provide species and strain, unique accession number if available, and source.	Not Applicable	

Human research participants	Information included in the manuscript?	In which section is the information available? (Reagents and Tools Table, Materials and Methods, Figures, Data Availability Section)
If collected and within the bounds of privacy constraints report on age, sex and gender or ethnicity for all study participants.	Yes	Material and Methods, Ehical Approvals

Core facilities	Information included in the manuscript?	In which section is the information available? (Reagents and Tools Table, Materials and Methods, Figures, Data Availability Section)
If your work benefited from core facilities, was their service mentioned in the acknowledgments section?	Yes	Acknowledgements

Design

**Study protocol**	**Information included in the manuscript?**	**In which section is the information available?** (Reagents and Tools Table, Materials and Methods, Figures, Data Availability Section)
If study protocol has been **pre-registered, provide DOI in the manuscript**. For clinical trials, provide the trial registration number **OR** cite DOI.	Not Applicable	
Report the **clinical trial registration number** (at ClinicalTrials.gov or equivalent), where applicable.	Not Applicable	

**Laboratory protocol**	**Information included in the manuscript?**	**In which section is the information available?** (Reagents and Tools Table, Materials and Methods, Figures, Data Availability Section)
Provide DOI OR other citation details if **external detailed step-by-step protocols** are available.	Not Applicable	

**Experimental study design and statistics**	**Information included in the manuscript?**	**In which section is the information available?** (Reagents and Tools Table, Materials and Methods, Figures, Data Availability Section)
Include a statement about **sample size** estimate even if no statistical methods were used.	Yes	Material and Methods, Figure legends
Were any steps taken to minimize the effects of subjective bias when allocating animals/samples to treatment (e.g. **randomization procedure**)? If yes, have they been described?	Yes	Material and Methods
Include a statement about **blinding** even if no blinding was done.	Yes	Material and Methods
Describe **inclusion/exclusion criteria** if samples or animals were excluded from the analysis. Were the criteria pre-established?<br><br>If sample or data points were omitted from analysis, report if this was due to attrition or intentional exclusion and provide justification.	Not Applicable	
For every figure, are **statistical tests** justified as appropriate? Do the data meet the assumptions of the tests (e.g., normal distribution)? Describe any methods used to assess it. Is there an estimate of variation within each group of data? Is the variance similar between the groups that are being statistically compared?	Yes	Material and Methods, Figure legends

**Sample definition and in-laboratory replication**	**Information included in the manuscript?**	**In which section is the information available?** (Reagents and Tools Table, Materials and Methods, Figures, Data Availability Section)
In the figure legends: state number of times the experiment was **replicated** in laboratory.	Yes	Figure legends, Material and Methods
In the figure legends: define whether data describe **technical or biological replicates**.	Yes	Figure legends, Material and Methods

Ethics

**Ethics**	**Information included in the manuscript?**	**In which section is the information available?** (Reagents and Tools Table, Materials and Methods, Figures, Data Availability Section)
Studies involving **human participants**: State details of **authority granting ethics approval** (IRB or equivalent committee(s), provide reference number for approval.	Yes	Ethical Approvals, Material and Methods
Studies involving **human participants**: Include a statement confirming that **informed consent** was obtained from all subjects and that the experiments conformed to the principles set out in the WMA Declaration of Helsinki and the Department of Health and Human Services Belmont Report.	Yes	Ethical Approvals
Studies involving **human participants:** For publication of **patient photos**, include a statement confirming that consent to publish was obtained.	Not Applicable	
Studies involving experimental **animals**: State details of **authority granting ethics approval** (IRB or equivalent committee(s), provide reference number for approval. Include a statement of compliance with ethical regulations.	Yes	Material and Methods
Studies involving **specimen and field samples:** State if relevant **permits** obtained, provide details of authority approving study; if none were required, explain why.	Not Applicable	

**Dual Use Research of Concern (DURC)**	**Information included in the manuscript?**	**In which section is the information available?** (Reagents and Tools Table, Materials and Methods, Figures, Data Availability Section)
Could your study fall under dual use research restrictions? Please check biosecurity documents and list of **select agents and toxins** (CDC): https://www.selectagents.gov/sat/list.htm	Not Applicable	
If you used a select agent, is the security level of the lab appropriate and reported in the manuscript?	Not Applicable	
If a study is subject to dual use research of concern regulations, is the name of the **authority granting approval and reference number** for the regulatory approval provided in the manuscript?	Not Applicable	

Reporting

The MDAR framework recommends adoption of discipline-specific guidelines, established and endorsed through community initiatives. Journals have their own policy about requiring specific guidelines and recommendations to complement MDAR.

**Adherence to community standards**	**Information included in the manuscript?**	**In which section is the information available?** (Reagents and Tools Table, Materials and Methods, Figures, Data Availability Section)
State if relevant guidelines or checklists (e.g., **ICMJE, MIBBI, ARRIVE, PRISMA**) have been followed or provided.	Not Applicable	
For **tumor marker prognostic studies,** we recommend that you follow the **REMARK** reporting guidelines (see link list at top right). See author guidelines, under 'Reporting Guidelines'. Please confirm you have followed these guidelines.	Not Applicable	
For **phase II and III randomized controlled trials**, please refer to the **CONSORT** flow diagram (see link list at top right) and submit the CONSORT checklist (see link list at top right) with your submission. See author guidelines, under 'Reporting Guidelines'. Please confirm you have submitted this list.	Not Applicable	

Data Availability

**Data availability**	**Information included in the manuscript?**	**In which section is the information available?** (Reagents and Tools Table, Materials and Methods, Figures, Data Availability Section)
Have **primary datasets** been deposited according to the journal's guidelines (see 'Data Deposition' section) and the respective accession numbers provided in the Data Availability Section?	Yes	Figure files (zipped files) and data availability section (Donders repository)
Were **human clinical and genomic datasets** deposited in a public access-controlled repository in accordance to ethical obligations to the patients and to the applicable consent agreement?	Not Applicable	
Are **computational models** that are central and integral to a study available without restrictions in a machine-readable form? Were the relevant accession numbers or links provided?	Yes	Data Availability section (Donders repository)
If publicly available data were reused, provide the respective **data citations in the reference list**.	Not Applicable	
