# Supplementary material for: Presynaptic accumulation of APP-CTFβ may contribute to synaptic dysfunction in Alzheimer’s disease
Source: EMBO J. 2026 Sep 1;45(19):6914–45. doi: 10.1038/s44318-026-00899-6 (PMC13631370; doi:10.1038/s44318-026-00899-6)
Supplement: Supplementary file 11 [file 44318_2026_899_MOESM3_ESM.zip › Movie EV1.docx]

**Movie EV1. Spontaneous calcium activity in Camk2-GCaMP6-expressing neurons following pharmacological modulation of APP processing**. Primary neurons (DIV21) transduced with AAV-Camk2-GCaMP6 were treated for 4 h with LY2886721 (1 μM; BACE1 inhibitor, BSI), DAPT (10 μM; γ-secretase inhibitor, GSI), or Aftin-4 (5 μM; APP processing activator, APA) to target distinct stages of APP proteolysis. Spontaneous calcium transients were recorded under Ca²⁺-containing Tyrode buffer.
