# Supplementary material for: Presynaptic accumulation of APP-CTFβ may contribute to synaptic dysfunction in Alzheimer’s disease
Source: EMBO J. 2026 Sep 1;45(19):6914–45. doi: 10.1038/s44318-026-00899-6 (PMC13631370; doi:10.1038/s44318-026-00899-6)
Supplement: Supplementary file 12 [file 44318_2026_899_MOESM4_ESM.zip › Movie EV2.docx]

**Movie EV2.** **Experimental workflow for dual imaging of neuronal Ca²⁺ dynamics and synaptic vesicle release.** Neurons expressing AAV.Camk2.GCaMP6 (transduced at DIV3) were treated with pharmacological modulators at DIV21 for 4 h. Synaptic vesicles were then labeled with SynaptoRed (SR), followed by washout and a 5-min rest period. Neurons were stimulated (30 Hz, 4-5 trains starting at 7 s, 30 s recovery), and dual imaging of GCaMP6 (*green*) and SR (*magenta*) signals was performed for ~2 min at 125 ms intervals. Fluorescence changes in individual ROIs (synapses-*magenta* and dendrites-*green*) were quantified.
