# Supplementary material for: Presynaptic accumulation of APP-CTFβ may contribute to synaptic dysfunction in Alzheimer’s disease
Source: EMBO J. 2026 Sep 1;45(19):6914–45. doi: 10.1038/s44318-026-00899-6 (PMC13631370; doi:10.1038/s44318-026-00899-6)
Supplement: Supplementary file 13 [file 44318_2026_899_MOESM5_ESM.zip › Movie EV3.docx]

**Movie EV3.** **APP-CTF–induced neuronal hyperexcitation *via* presynaptic dysfunction.** Neurons expressing AAV.Camk2.GCaMP6 were treated with pharmacological modulators to alter APP processing (as above). Dual imaging of neuronal Ca²⁺ dynamics (GCaMP6, *green*) and synaptic vesicle release (SR, *magenta*) was performed as indicated above. GSI treatment resulted in enhanced Ca²⁺ responses and impaired vesicle release consistent with presynaptic dysfunction.
