# Supplementary material for: Presynaptic accumulation of APP-CTFβ may contribute to synaptic dysfunction in Alzheimer’s disease
Source: EMBO J. 2026 Sep 1;45(19):6914–45. doi: 10.1038/s44318-026-00899-6 (PMC13631370; doi:10.1038/s44318-026-00899-6)
Supplement: Supplementary file 14 [file 44318_2026_899_MOESM6_ESM.zip › Movie EV4.docx]

**Movie EV4.** **Aβ rescues γ-secretase inhibitor (GSI)-induced hyperactivity via postsynaptic mechanisms.** Neurons were treated with γ-secretase inhibitor (GSI) to increase APP-CTF accumulation. GSI pretreated or untreated neurons were subjected to acute treatment with Aβ for 5 min, before imaging. Dual imaging of neuronal Ca²⁺ dynamics (GCaMP6, *green*) and synaptic vesicle release (SR, *magenta*) was performed as indicated above. Data indicate that Aβ treatment normalizes GSI-induced hyperactivity, implicating a postsynaptic mechanism of rescue.
