# Supplementary material for: Presynaptic accumulation of APP-CTFβ may contribute to synaptic dysfunction in Alzheimer’s disease
Source: EMBO J. 2026 Sep 1;45(19):6914–45. doi: 10.1038/s44318-026-00899-6 (PMC13631370; doi:10.1038/s44318-026-00899-6)
Supplement: Supplementary file 15 [file 44318_2026_899_MOESM7_ESM.zip › Movie EV5.docx]

**Movie EV5. All atom molecular dynamics simulation of APP-CTFβ (C99) in a realistic presynaptic membrane.** (**A**) The video depicts an all-atom molecular dynamics simulation of APP-CTFβ embedded in a lipid bilayer that closely reflects the composition of a presynaptic membrane. The simulation models C99 in a physiologically relevant environment over 500 ns. Arg76, located near the C-terminal end, engages in stable interactions with the PIP2 headgroup (red). (**B**) A close-up view highlights the interaction between Arg76 and the PIP2 headgroup, as well as the dipping of the C-terminal helix into the inner leaflet of the membrane. (**C**) Interaction energies between Arg76 and the PIP2 headgroup are shown, separated into Coulombic (red) and Lennard-Jones (blue) contributions.
