# Supplementary material for: Presynaptic accumulation of APP-CTFβ may contribute to synaptic dysfunction in Alzheimer’s disease
Source: EMBO J. 2026 Sep 1;45(19):6914–45. doi: 10.1038/s44318-026-00899-6 (PMC13631370; doi:10.1038/s44318-026-00899-6)
Supplement: Supplementary file 16 [file 44318_2026_899_MOESM8_ESM.zip › Movie EV6.docx]

**Movie EV6. APP-CTFβ forms trimers in a realistic presynaptic membrane.** The video depicts a 5-microsecond molecular dynamics simulation of three APP-CTFβ (C99) motifs embedded in a realistic presynaptic membrane, colored individually in pink, blue, and green. During the simulation, two motifs first associate to form a dimer (*purple*), followed by the emergence of a trimer (*dark green*), consistent with the kinetics illustrated in the accompanying line plot (*right*).
