# Supplementary material for: Presynaptic accumulation of APP-CTFβ may contribute to synaptic dysfunction in Alzheimer’s disease
Source: EMBO J. 2026 Sep 1;45(19):6914–45. doi: 10.1038/s44318-026-00899-6 (PMC13631370; doi:10.1038/s44318-026-00899-6)

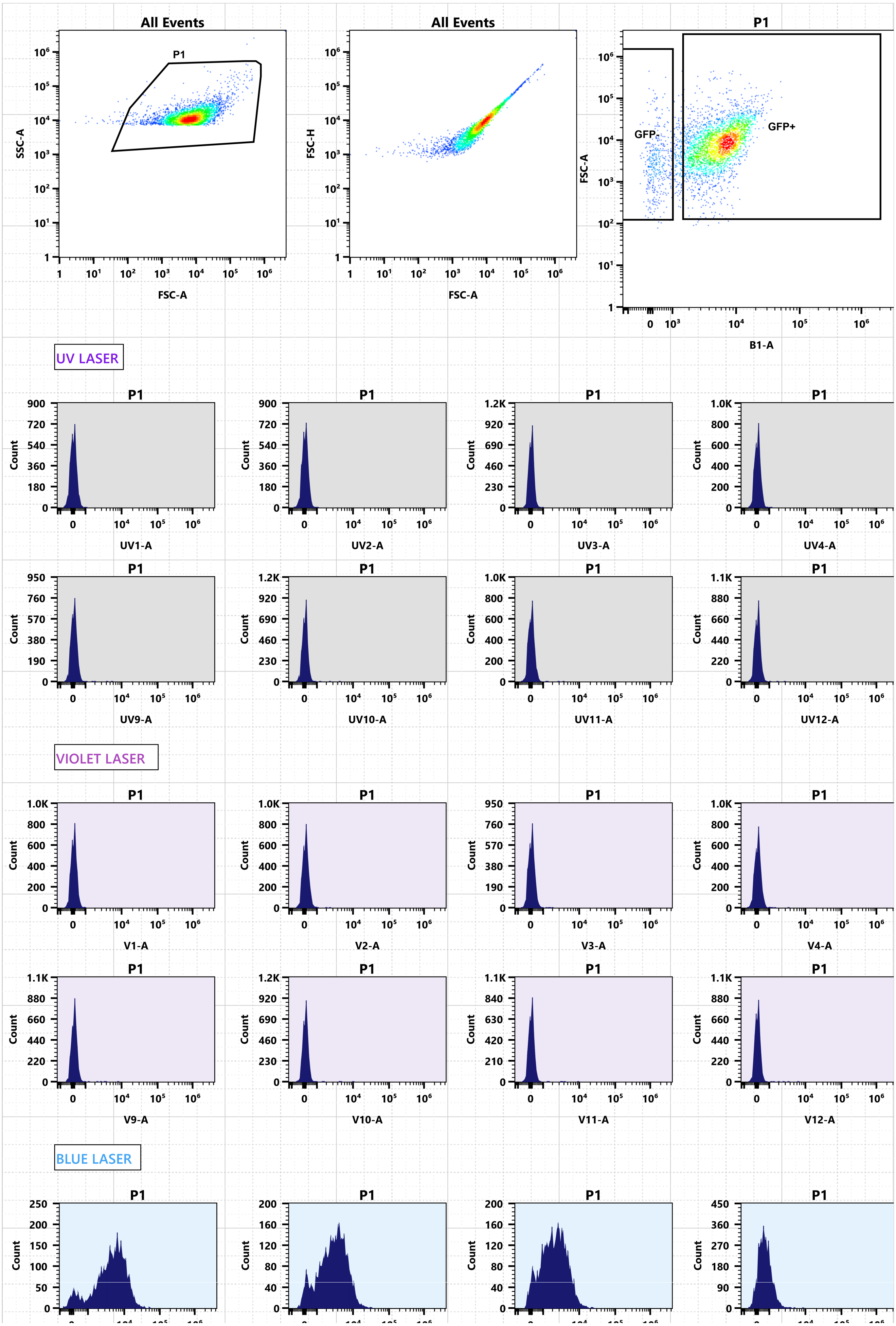

All Events
P1
SSC-A
FSC-A
All Events
FSC-H
FSC-A
P1
GFP-
GFP+
FSC-A
B1-A
UV LASER
P1
Count
UV1-A
UV2-A
UV3-A
UV4-A
UV9-A
UV10-A
UV11-A
UV12-A
VIOLET LASER
V1-A
V2-A
V3-A
V4-A
V9-A
V10-A
V11-A
V12-A
BLUE LASER

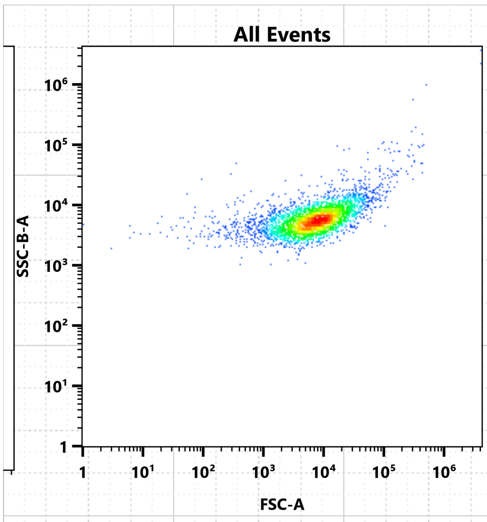

Population Hiera
Synaps CB-20240528-(

Population	% Parent
P1	87.27
P5	86.99
P6	10.54
P7	10.68
GFP+	83.88
GFP-	11.13

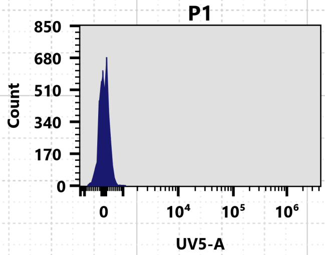

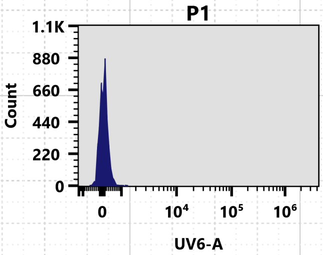

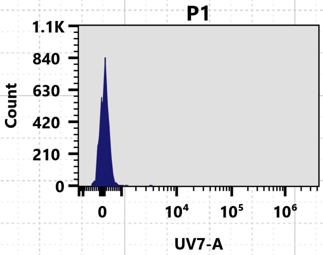

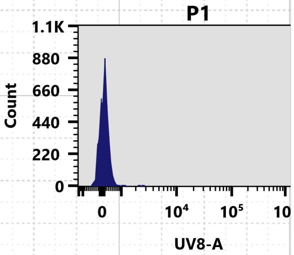

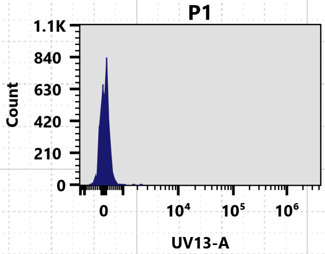

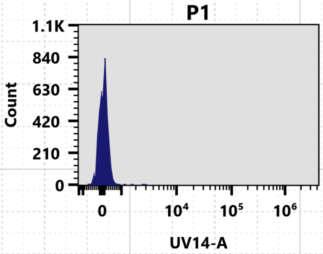

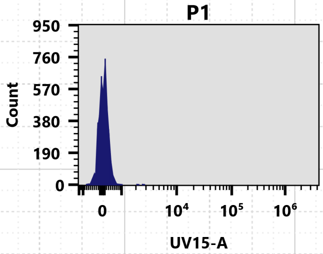

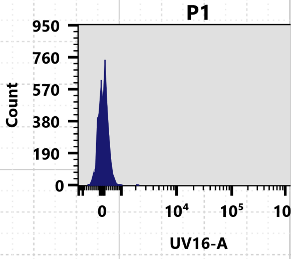

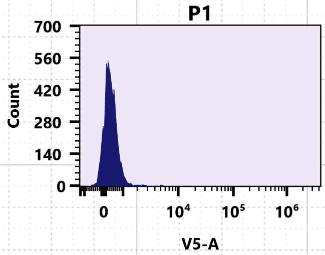

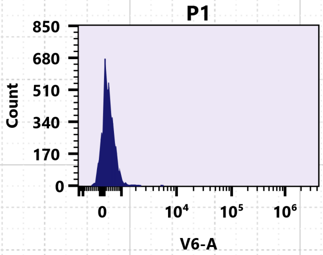

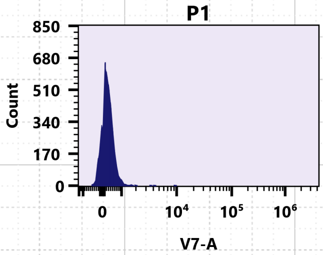

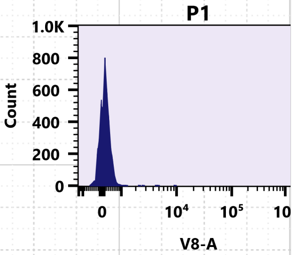

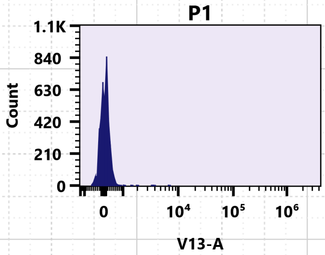

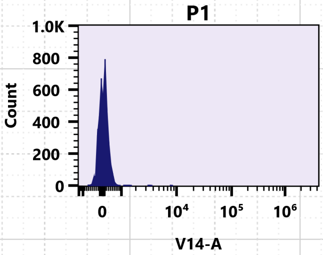

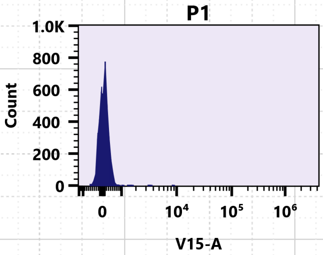

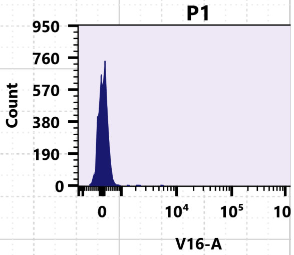

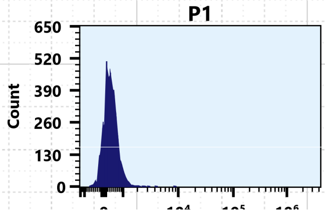

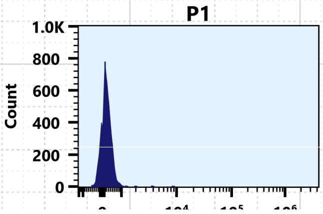

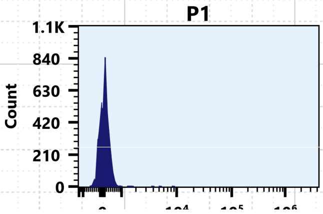

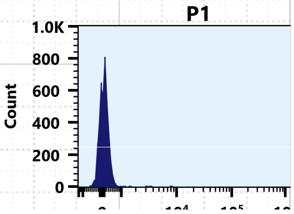

rchy
GFP rerun

	Count
	3,728
	3,243
	393
	398
	3,127
	415

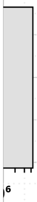

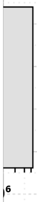

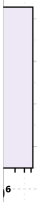

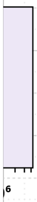

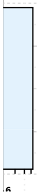

B1-A B2-A B3-A B4-A

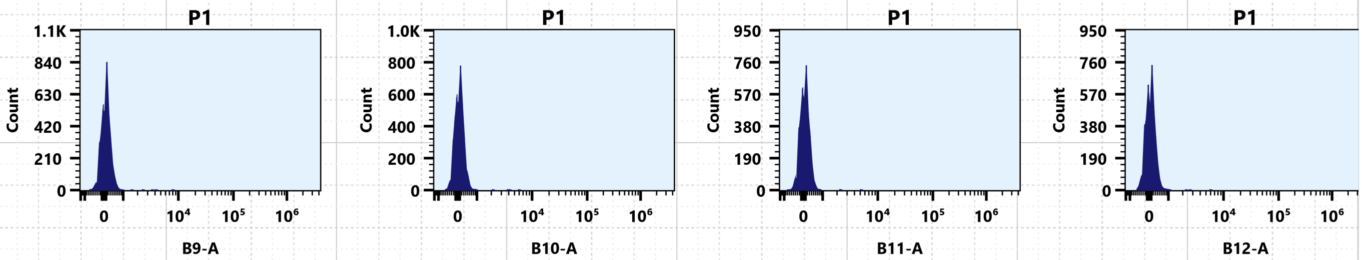

YELLOWGREEN LASER

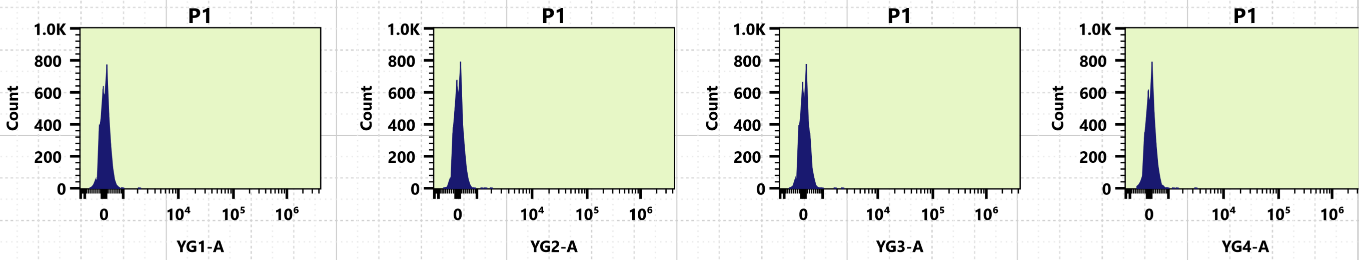

RED LASER

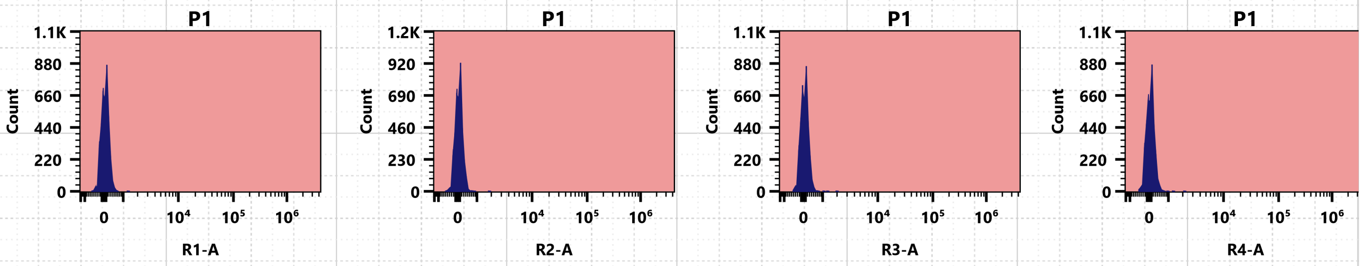

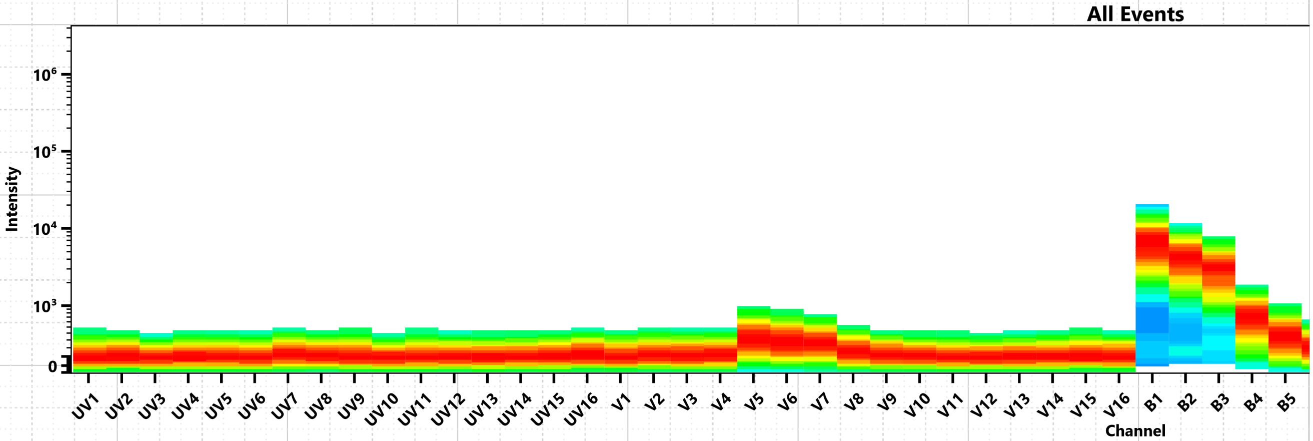

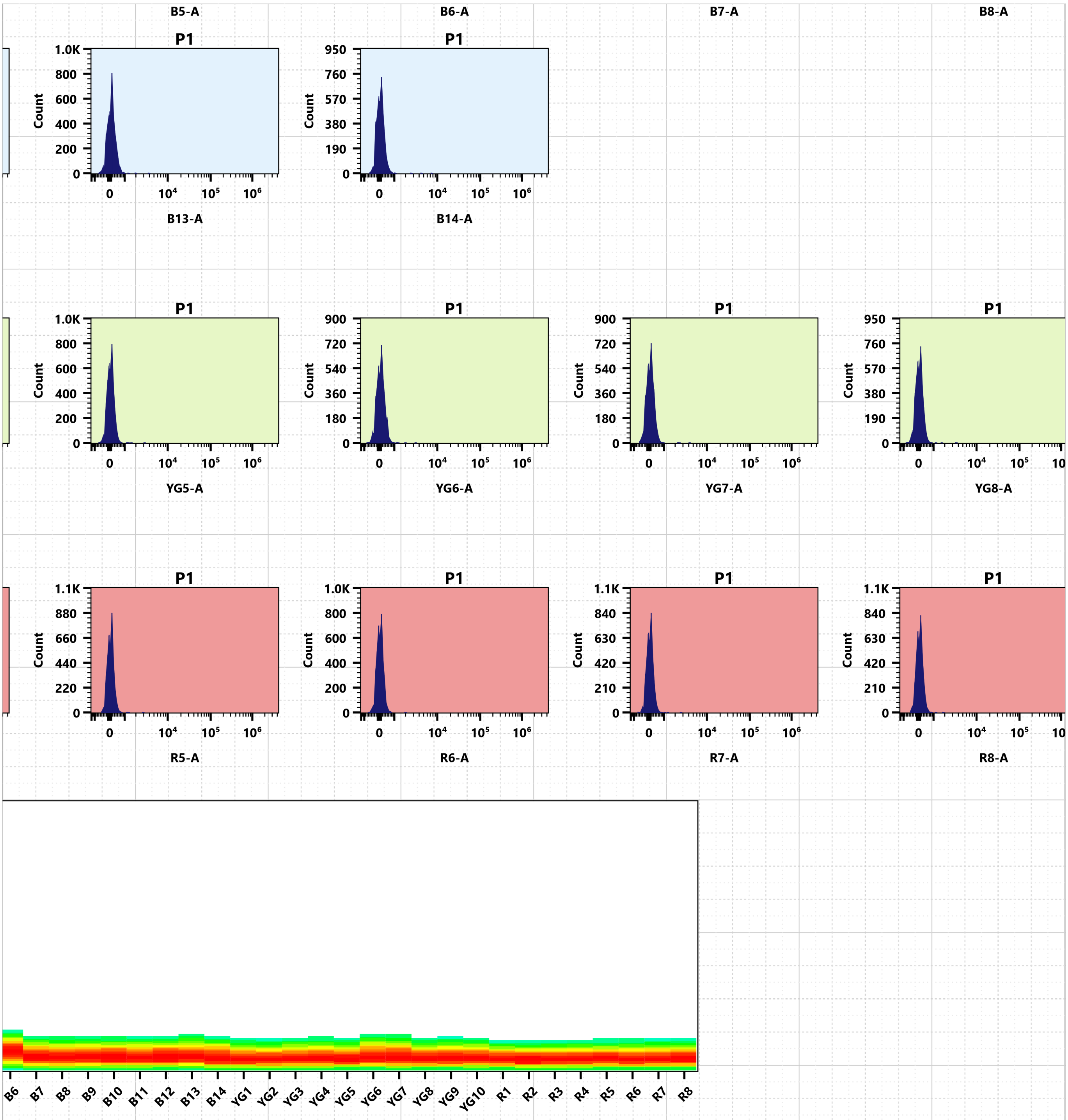

B5-A
B6-A
B7-A
B8-A
P1
Count
B13-A
B14-A
YG5-A
YG6-A
YG7-A
YG8-A
R5-A
R6-A
R7-A
R8-A
B6
B7
B8
B9
B10
B11
B12
B13
B14
YG1
YG2
YG3
YG4
YG5
YG6
YG7
YG8
YG9
YG10
R1
R2
R3
R4
R5
R6
R7
R8

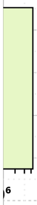

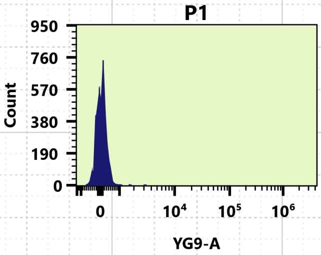
P1
950
760
570
380
190
0
Count
0
YG9-A

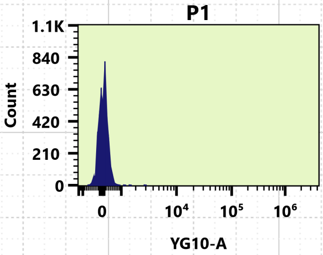
P1
1.1K
840
630
420
210
0
Count
0
YG10-A

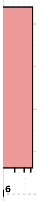

Supplement: Supplementary file 10 [file 44318_2026_899_MOESM16_ESM.zip › Figures EV1-4/Figure EV1/Fig. EV1B-C/data_files.zip!/GFP rerun.pdf]
