# Supplementary material for: Presynaptic accumulation of APP-CTFβ may contribute to synaptic dysfunction in Alzheimer’s disease
Source: EMBO J. 2026 Sep 1;45(19):6914–45. doi: 10.1038/s44318-026-00899-6 (PMC13631370; doi:10.1038/s44318-026-00899-6)
Supplement: Supplementary file 10 [file 44318_2026_899_MOESM16_ESM.zip › Figures EV1-4/Figure EV1/Fig. EV1B-C/data_files.zip!/GFP rerun_end.pdf]

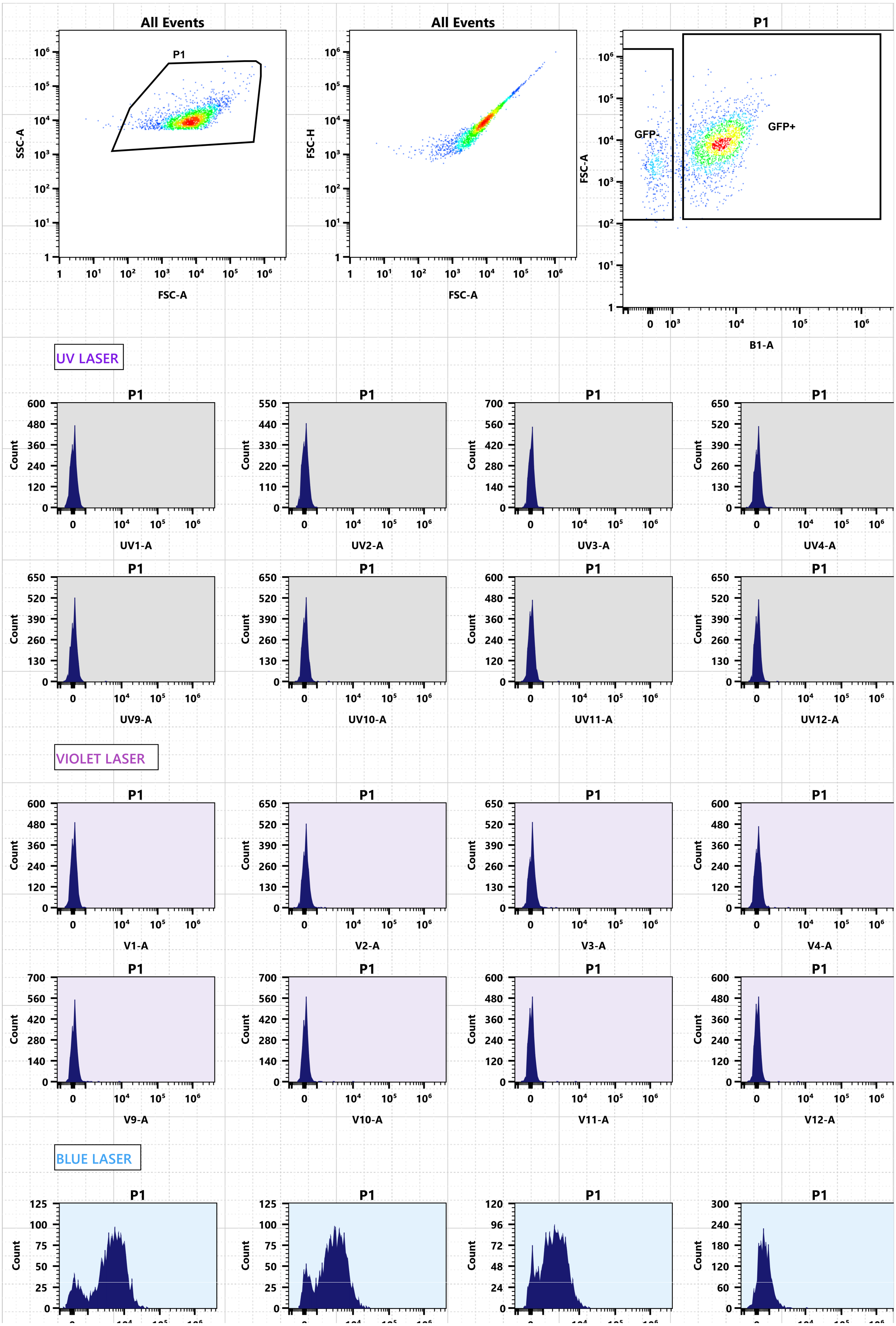

All Events
P1
SSC-A
FSC-A
All Events
FSC-H
FSC-A
P1
GFP-
GFP+
FSC-A
B1-A
UV LASER
P1
Count
UV1-A
UV2-A
UV3-A
UV4-A
UV9-A
UV10-A
UV11-A
UV12-A
VIOLET LASER
V1-A
V2-A
V3-A
V4-A
V9-A
V10-A
V11-A
V12-A
BLUE LASER

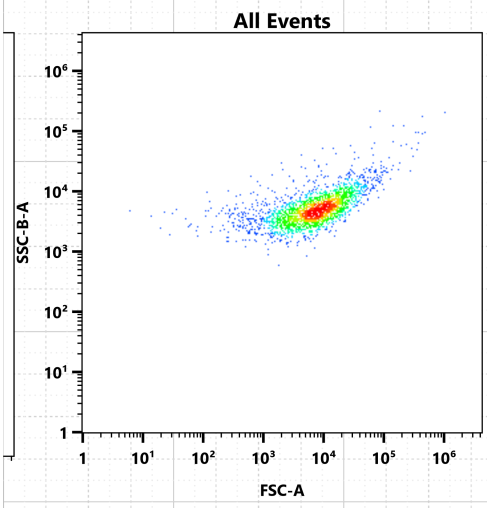

Population Hierarchy
Synaps CB-20240528-GFP rerun_end

Population	% Parent	Count
P1	87.96	2,280
P5	83.55	1,905
P6	14.65	334
P7	8.99	205
GFP+	81.10	1,849
GFP-	15.31	349

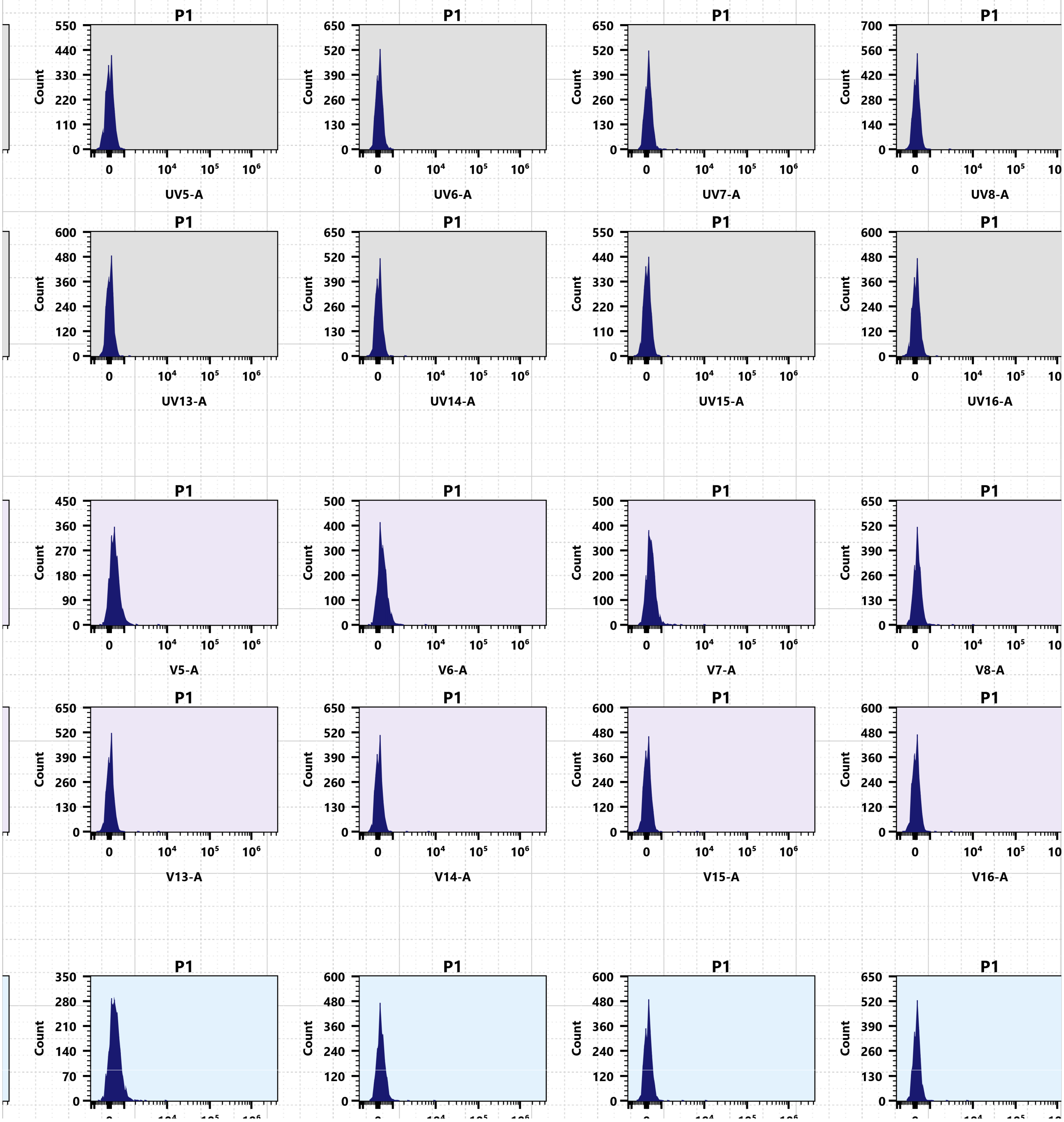

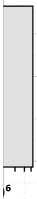
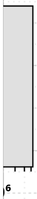
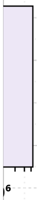
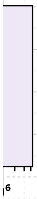
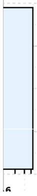

B1-A B2-A B3-A B4-A

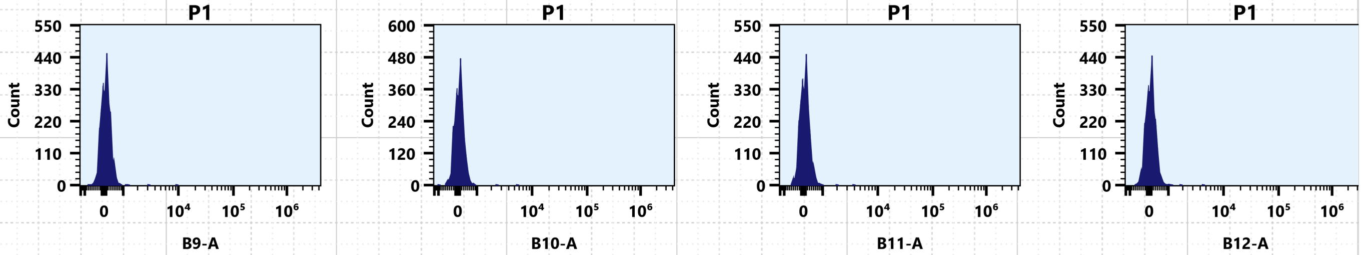

YELLOWGREEN LASER

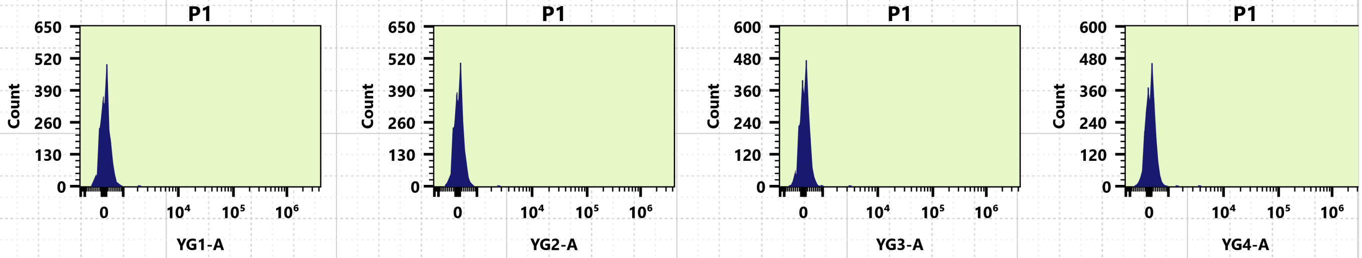

RED LASER

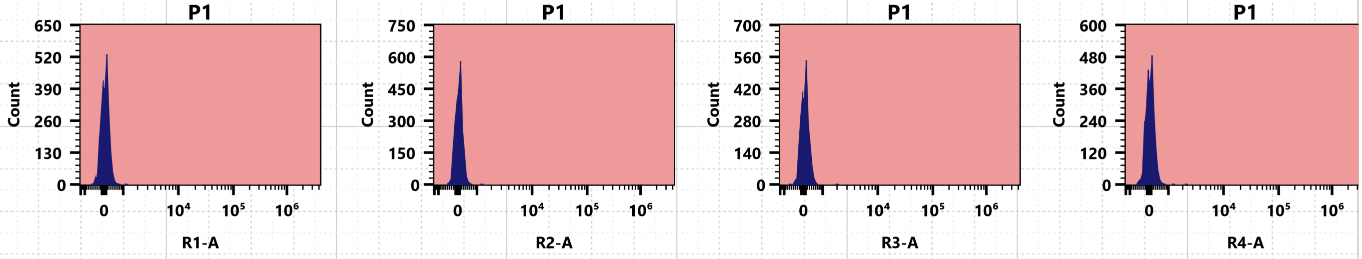

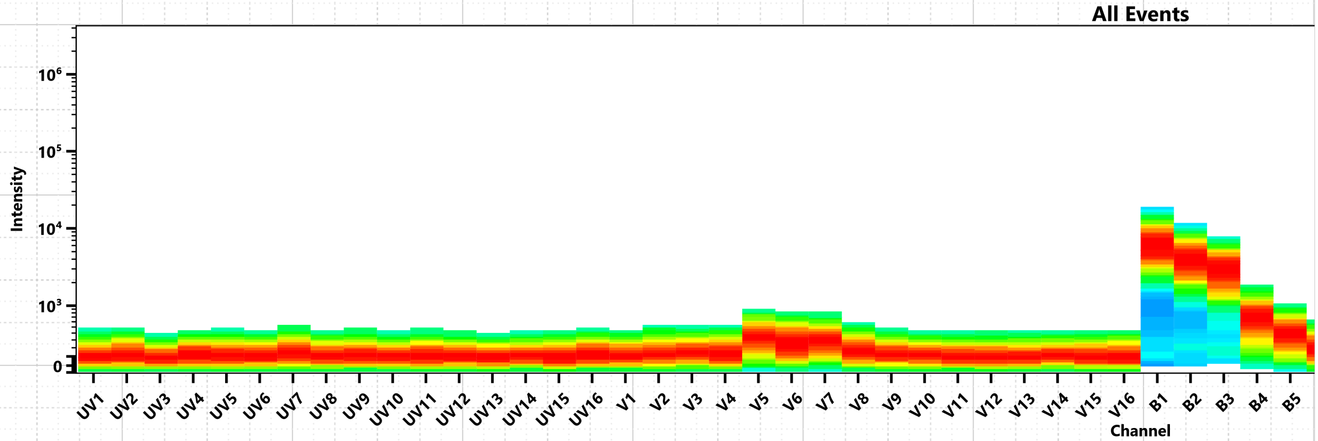

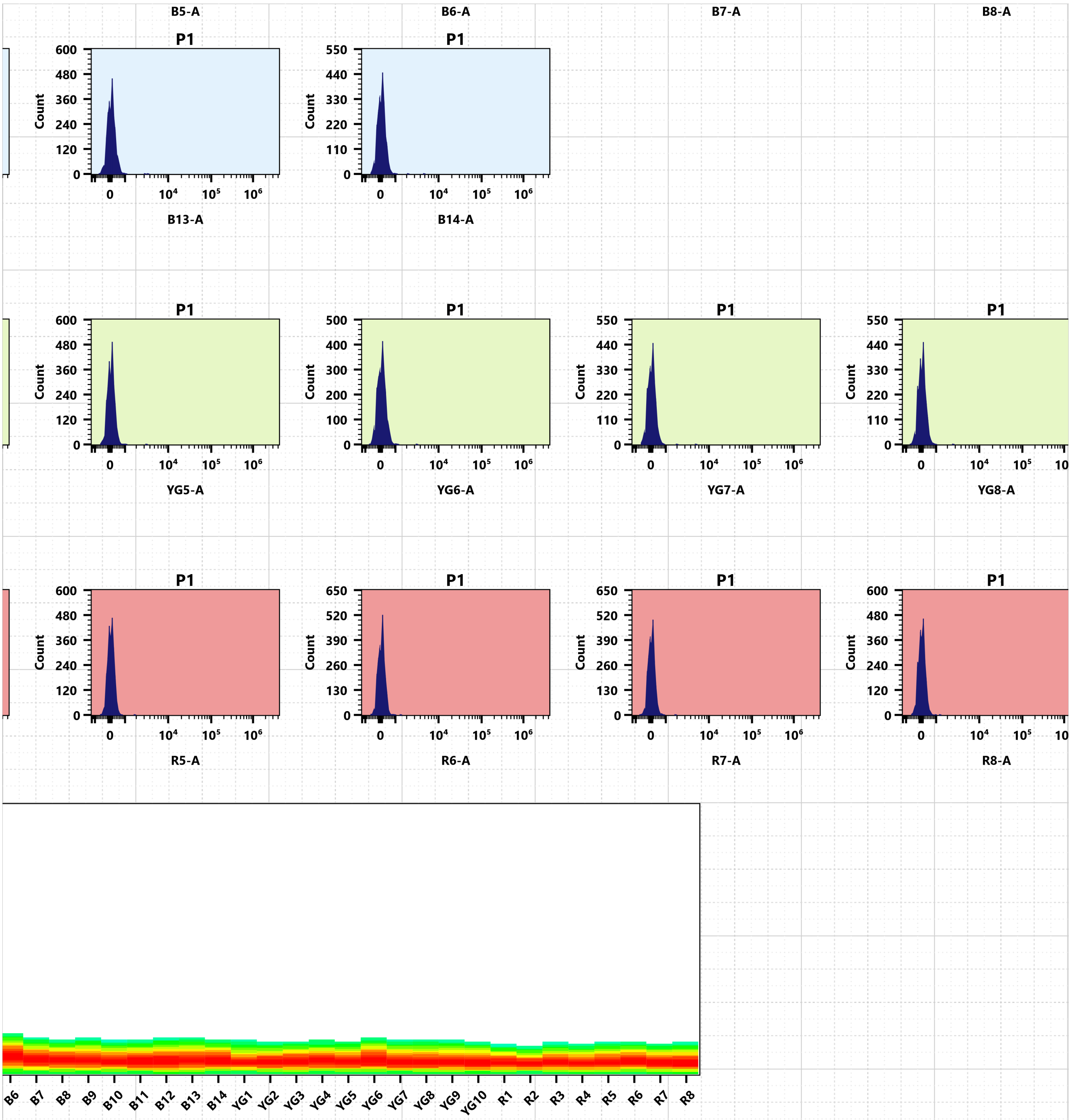
B5-A
B6-A
B7-A
B8-A
P1
Count
B13-A
B14-A
YG5-A
YG6-A
YG7-A
YG8-A
R5-A
R6-A
R7-A
R8-A
B6 B7 B8 B9 B10 B11 B12 B13 B14 YG1 YG2 YG3 YG4 YG5 YG6 YG7 YG8 YG9 YG10 R1 R2 R3 R4 R5 R6 R7 R8

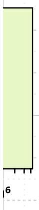

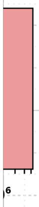

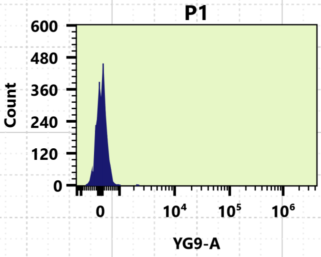
P1
Count
600
480
360
240
120
0
0
10⁴
10⁵
10⁶
YG9-A

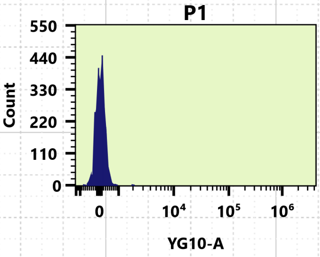
P1
Count
550
440
330
220
110
0
0
10⁴
10⁵
10⁶
YG10-A
