# Supplementary material for: Presynaptic accumulation of APP-CTFβ may contribute to synaptic dysfunction in Alzheimer’s disease
Source: EMBO J. 2026 Sep 1;45(19):6914–45. doi: 10.1038/s44318-026-00899-6 (PMC13631370; doi:10.1038/s44318-026-00899-6)
Supplement: Supplementary file 10 [file 44318_2026_899_MOESM16_ESM.zip › Figures EV1-4/Figure EV1/Fig. EV1B-C/data_files.zip!/sort report 3.pdf]

Synaps CB Report_038

Sort Details

Date	Start Time	Instrument Configuration	Sort Threshold Count	Group Name
May 28, 2024	15:05:17 PM	5-Laser-V16-B14-R8-YG10-UV16	43,135,911	20240528
**Operator**	**Stop Time**	**Instrument Serial Number**	**E-Abort Count**	**Tube Name**
Overig_Project36	15:53:28 PM	S0239	2,524,482	vglut-GFP

Sort Parameters

Nozzle Size and S/N	Sort Mode	Drop Delay Value	Amplitude	Drop Center - Setpoint	Drop Interval - Setpoint	Plate Voltage (V)	Center Stream Optimization	Temperature Control
70 µm K27153	Multiway	4849	22151	263	9	5,000 V	4th drop: 3, 3rd: 5, 2nd: 5, 1st: 18	4
**Nozzle Setting**	**Sort Type**	**Drop Drive Frequency**	**Sort Monitoring**	**Drop Center - Actual**	**Drop Interval - Actual**	**Drop Charge**	**Sheath Pressure**	**Mixing**
Operator (CHL_Operator_Rob)	Tube	79000	On	263	9	L:-88, R:70	64.5 psi	High

Tubes

LEFT

Population Name
GFP-

Total Count
8999774

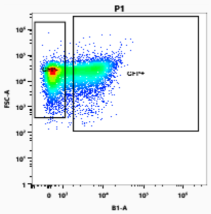

RIGHT

Population Name
GFP+

Total Count
10103734

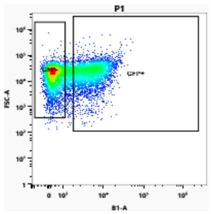

Tube	LEFT		RIGHT	
1	Starting Volume: 0.00 µl	Sorted Volume: 11251.17 µl	Starting Volume: 100.00 µl	Sorted Volume: 11155.45 µl
	Average Sort Rate: 3152 e/s	Count: 8998777	Average Sort Rate: 3472 e/s	Count: 8908119
	Average Abort Rate: 581 e/s	Abort Count: 1658657	Average Abort Rate: 910 e/s	Abort Count: 2334781
	Average Efficiency: 84%	Quick Stop: None	Average Efficiency: 79%	Quick Stop: None
	Sort Threshold Count: 43132519	Waste Tank Full: False	Sort Threshold Count: 38476400	Waste Tank Full: False
	% Total: 18.8372		% Total: 20.6672	
2	Starting Volume: 0.00 µl	Sorted Volume: 1.25 µl	Starting Volume: 100.00 µl	Sorted Volume: 1494.72 µl
	Average Sort Rate: 34 e/s	Count: 997	Average Sort Rate: 3734 e/s	Count: 1195615
	Average Abort Rate: 3 e/s	Abort Count: 90	Average Abort Rate: 1007 e/s	Abort Count: 322320
	Average Efficiency: 92%	Quick Stop: None	Average Efficiency: 79%	Quick Stop: None
	Sort Threshold Count: 3392	Waste Tank Full: False	Sort Threshold Count: 4659511	Waste Tank Full: False
	% Total: 39.9759		% Total: 25.5952	

Comments

Type to add a comment...

Submit Comment
