# Supplementary material for: Presynaptic accumulation of APP-CTFβ may contribute to synaptic dysfunction in Alzheimer’s disease
Source: EMBO J. 2026 Sep 1;45(19):6914–45. doi: 10.1038/s44318-026-00899-6 (PMC13631370; doi:10.1038/s44318-026-00899-6)
Supplement: Supplementary file 10 [file 44318_2026_899_MOESM16_ESM.zip › Figures EV1-4/Figure EV1/Fig. EV1B-C/data_files.zip!/sort report.pdf]

Synaps CB Report_036

Sort Details

Date	Start Time	Instrument Configuration	Sort Threshold Count	Group Name
May 28, 2024	13:16:44 PM	5-Laser-V16-B14-R8-YG10-UV16	11,970,948	20240528
**Operator**	**Stop Time**	**Instrument Serial Number**	**E-Abort Count**	**Tube Name**
Overig_Project36	13:32:05 PM	S0239	626,527	vglut-GFP

Sort Parameters

Nozzle Size and S/N	Sort Mode	Drop Delay Value	Amplitude	Drop Center - Setpoint	Drop Interval - Setpoint	Plate Voltage (V)	Center Stream Optimization	Temperature Control
70 µm K27153	Multiway	4829	20177	267	9	5,000 V	4th drop: 3, 3rd: 5, 2nd: 5, 1st: 18	4
**Nozzle Setting**	**Sort Type**	**Drop Drive Frequency**	**Sort Monitoring**	**Drop Center - Actual**	**Drop Interval - Actual**	**Drop Charge**	**Sheath Pressure**	**Mixing**
Operator (CHL_Operator_Rob)	Tube	79000	On	263	9	L:-88, R:70	64.5 psi	High

Tubes

LEFT

Population Name
P12

Total Count
3360430

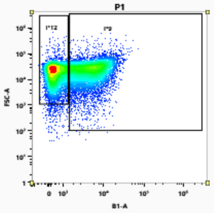

RIGHT

Population Name
P9

Total Count
3018928

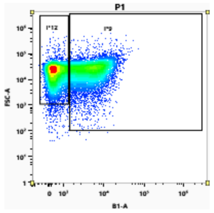

1

	LEFT		RIGHT	
	Starting Volume: 0.00 µl	Sorted Volume: 4197.68 µl	Starting Volume: 100.00 µl	Sorted Volume: 3782.34 µl
	Average Sort Rate: 3661 e/s	Count: 3360430	Average Sort Rate: 3289 e/s	Count: 3018928
	Average Abort Rate: 521 e/s	Abort Count: 478614	Average Abort Rate: 763 e/s	Abort Count: 700540
	Average Efficiency: 88%	Quick Stop: None	Average Efficiency: 81%	Quick Stop: None
	Sort Threshold Count: 11970948	Waste Tank Full: False	Sort Threshold Count: 11970948	Waste Tank Full: False
	% Total: 23.7883		% Total: 21.3709	

Comments

Type to add a comment...

Submit Comment
