# Supplementary material for: Presynaptic accumulation of APP-CTFβ may contribute to synaptic dysfunction in Alzheimer’s disease
Source: EMBO J. 2026 Sep 1;45(19):6914–45. doi: 10.1038/s44318-026-00899-6 (PMC13631370; doi:10.1038/s44318-026-00899-6)
Supplement: Supplementary file 10 [file 44318_2026_899_MOESM16_ESM.zip › Figures EV1-4/Figure EV1/Fig. EV1B-C/data_files.zip!/sortreport 2.pdf]

Synaps CB Report_037

Sort Details

Date	Start Time	Instrument Configuration	Sort Threshold Count	Group Name
May 28, 2024	13:38:38 PM	5-Laser-V16-B14-R8-YG10-UV16	34,287,899	20240528
**Operator**	**Stop Time**	**Instrument Serial Number**	**E-Abort Count**	**Tube Name**
Overig_Project36	14:27:20 PM	S0239	1,683,148	vglut-GFP

Sort Parameters

Nozzle Size and S/N	Sort Mode	Drop Delay Value	Amplitude	Drop Center - Setpoint	Drop Interval - Setpoint	Plate Voltage (V)	Center Stream Optimization	Temperature Control
70 µm K27153	Multiway	4829	21035	267	9	5,000 V	4th drop: 3, 3rd: 5, 2nd: 5, 1st: 18	4
**Nozzle Setting**	**Sort Type**	**Drop Drive Frequency**	**Sort Monitoring**	**Drop Center - Actual**	**Drop Interval - Actual**	**Drop Charge**	**Sheath Pressure**	**Mixing**
Operator (CHL_Operator_Rob)	Tube	79000	On	265	1	L:-88, R:70	64.5 psi	High

Tubes

LEFT

Population Name
P12

Total Count
6633401

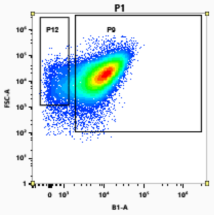

RIGHT

Population Name
P9

Total Count
11082360

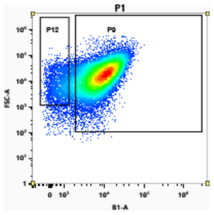

	LEFT		RIGHT	
1	Starting Volume: 0.00 µl	Sorted Volume: 7120.83 µl	Starting Volume: 100.00 µl	Sorted Volume: 11153.30 µl
	Average Sort Rate: 2356 e/s	Count: 5674783	Average Sort Rate: 3697 e/s	Count: 8903206
	Average Abort Rate: 360 e/s	Abort Count: 867490	Average Abort Rate: 733 e/s	Abort Count: 1764243
	Average Efficiency: 87%	Quick Stop: None	Average Efficiency: 83%	Quick Stop: None
	Sort Threshold Count: 28438658	Waste Tank Full: False	Sort Threshold Count: 28438658	Waste Tank Full: False
	% Total: 19.8425		% Total: 31.1311	
2	Starting Volume: 0.00 µl	Sorted Volume: 1204.49 µl	Starting Volume: 100.00 µl	Sorted Volume: 2721.37 µl
	Average Sort Rate: 2195 e/s	Count: 958618	Average Sort Rate: 4989 e/s	Count: 2179154
	Average Abort Rate: 486 e/s	Abort Count: 212216	Average Abort Rate: 1281 e/s	Abort Count: 559562
	Average Efficiency: 82%	Quick Stop: None	Average Efficiency: 80%	Quick Stop: None
	Sort Threshold Count: 5849241	Waste Tank Full: False	Sort Threshold Count: 5849241	Waste Tank Full: False
	% Total: 16.3005		% Total: 37.0546	

Comments

Type to add a comment...

Submit Comment
