# Supplementary material for: Presynaptic accumulation of APP-CTFβ may contribute to synaptic dysfunction in Alzheimer’s disease
Source: EMBO J. 2026 Sep 1;45(19):6914–45. doi: 10.1038/s44318-026-00899-6 (PMC13631370; doi:10.1038/s44318-026-00899-6)

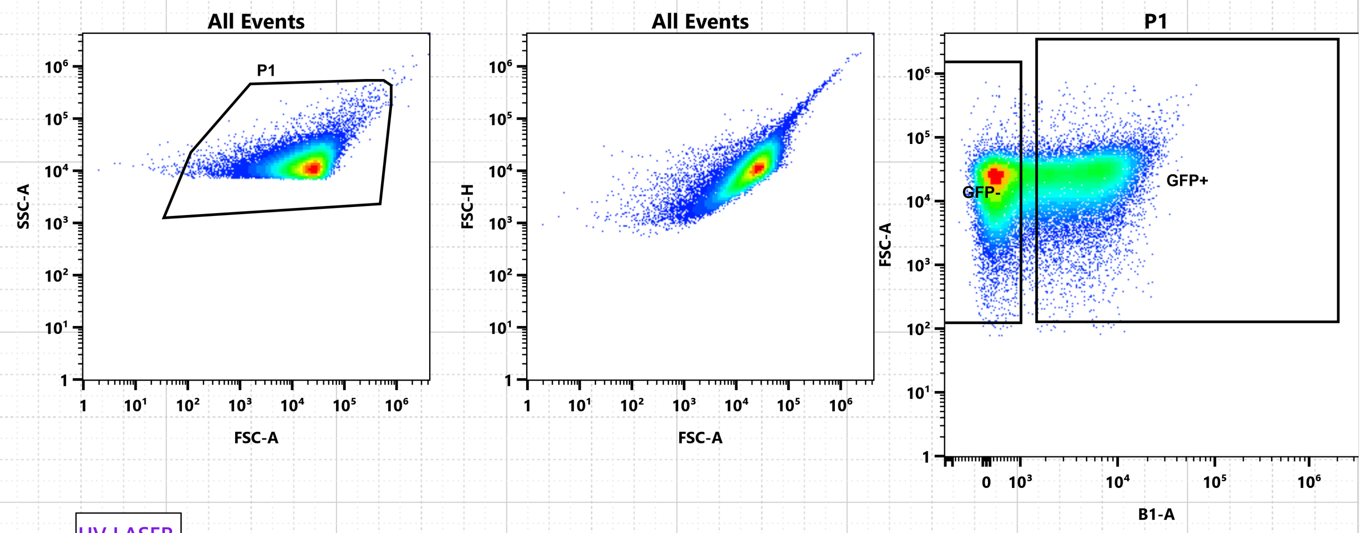

UV LASER

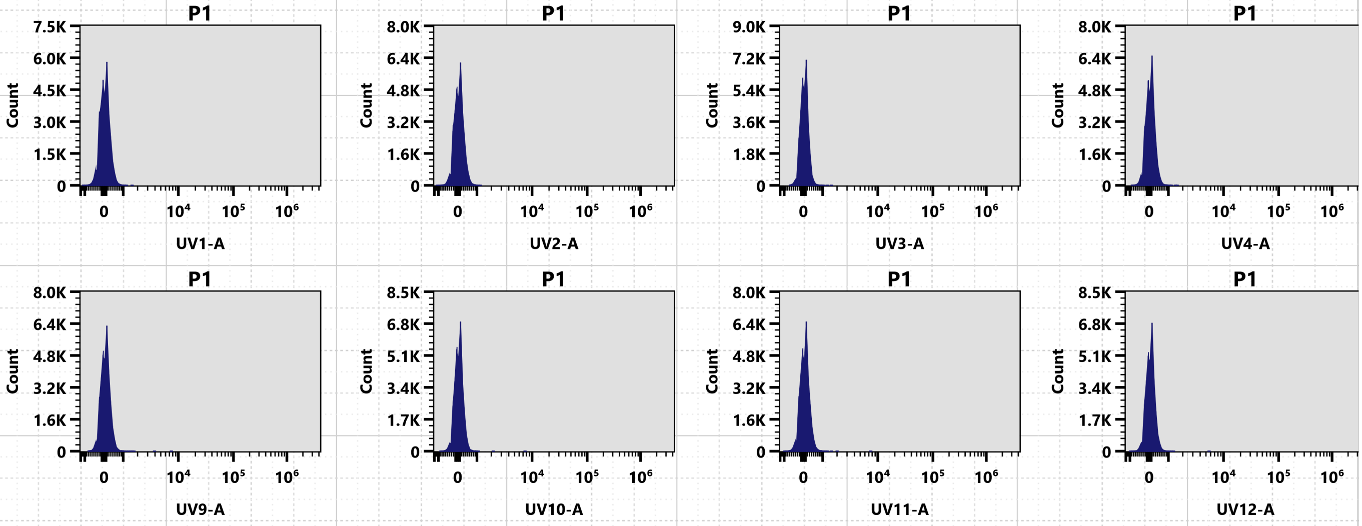

VIOLET LASER

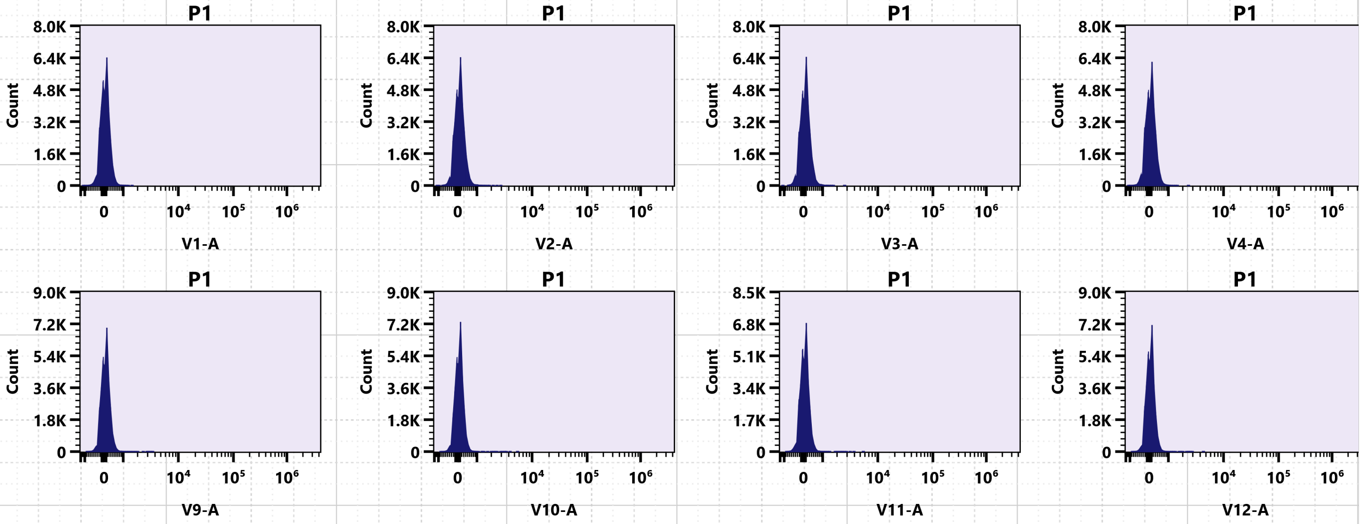

BLUE LASER

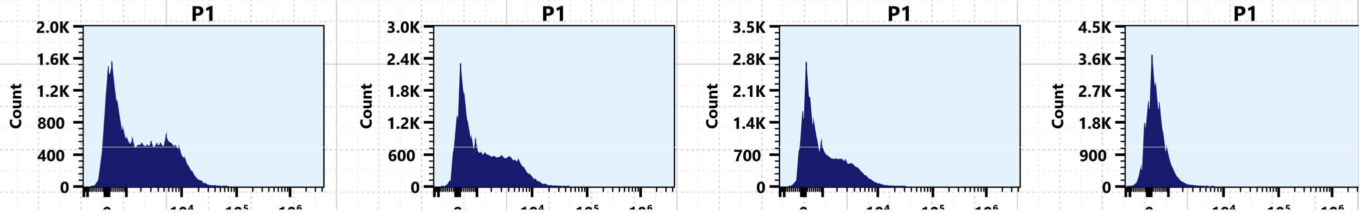

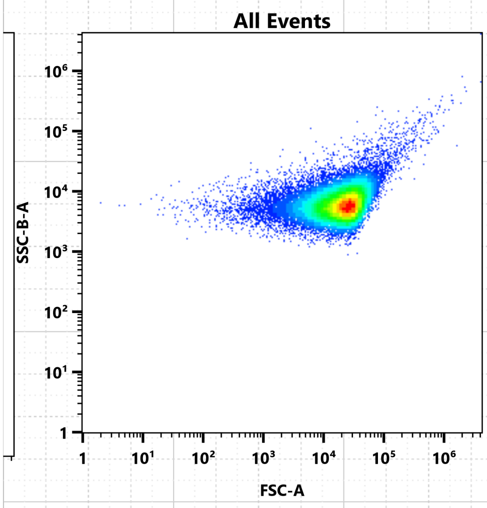

Population Hiera
Synaps CB-20240528-

Population		% Parent
P1		61.08
	P5	51.67
	P6	43.24
	P7	43.61
	GFP+	47.40
	GFP-	44.57

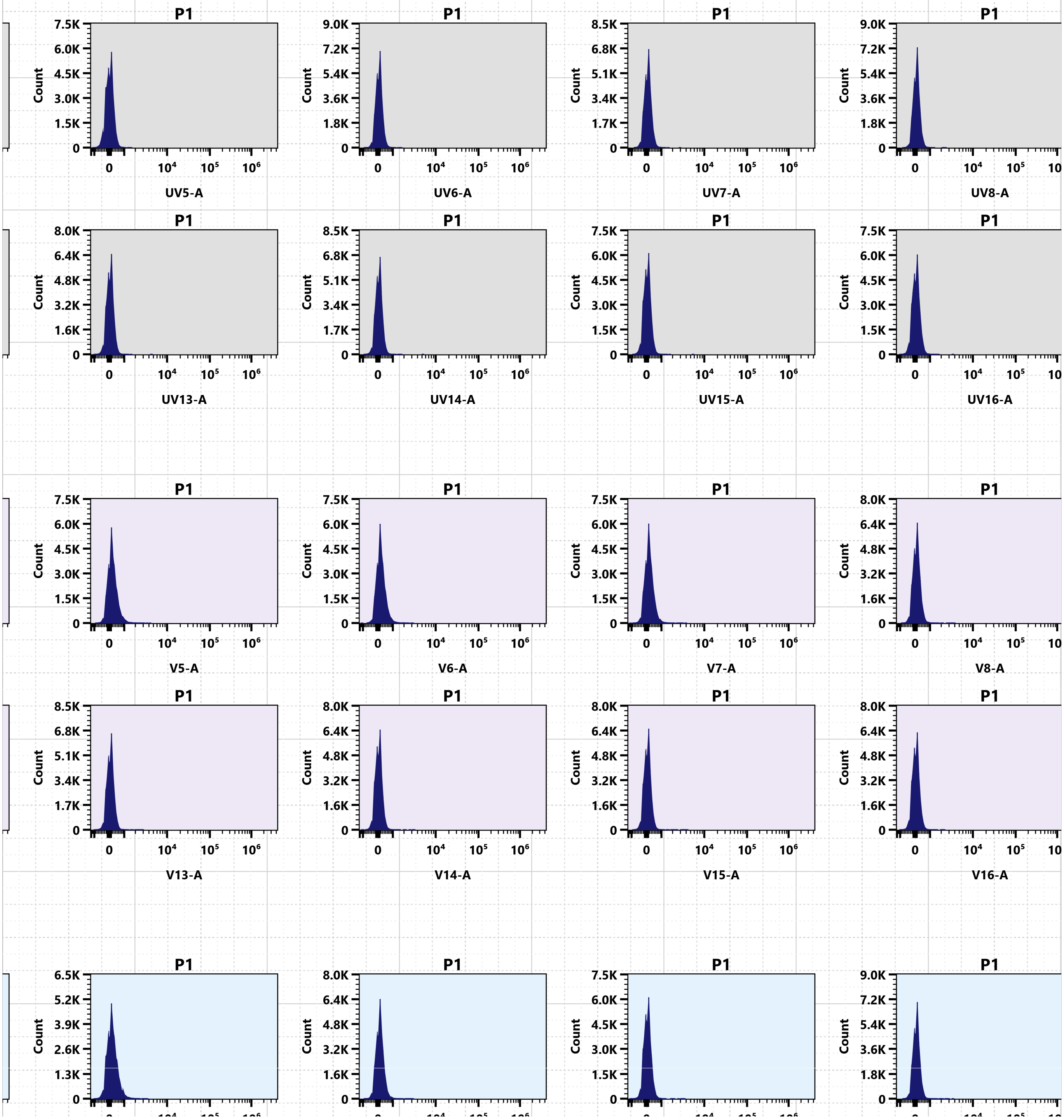

rchy
vglut-GFP

	Count
	30,541
	15,782
	13,205
	13,320
	14,475
	13,613

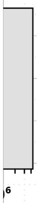

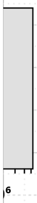

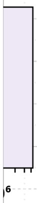

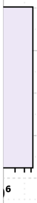

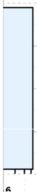

B1-A B2-A B3-A B4-A

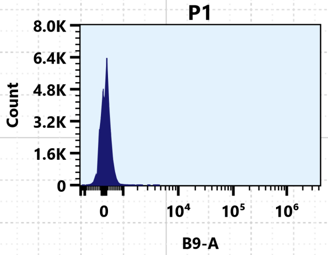

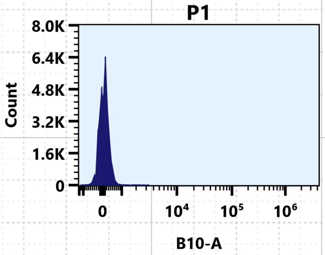

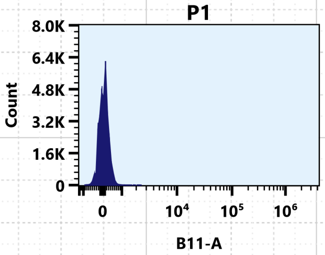

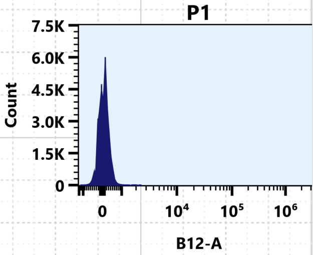

YELLOWGREEN LASER

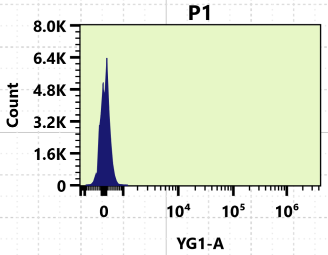

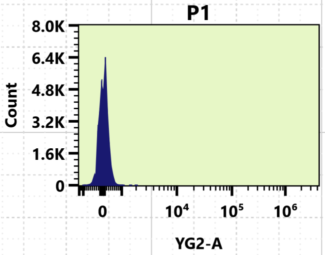

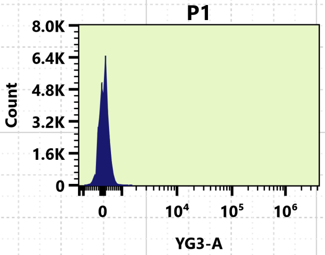

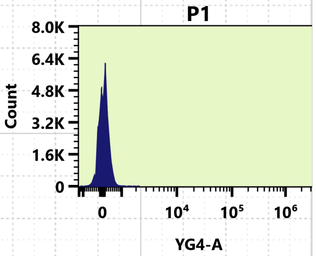

RED LASER

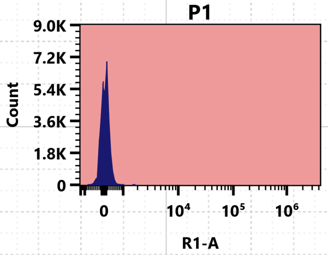

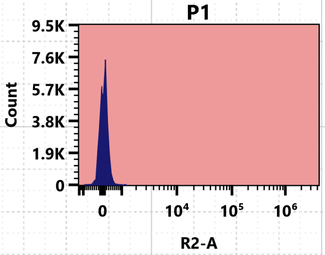

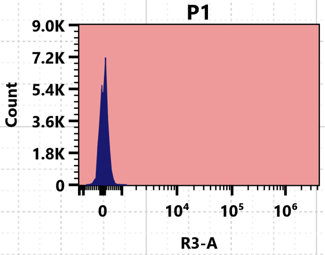

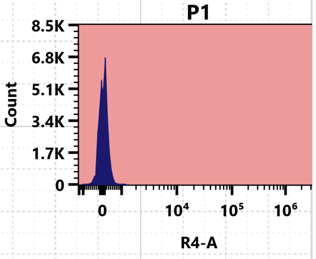

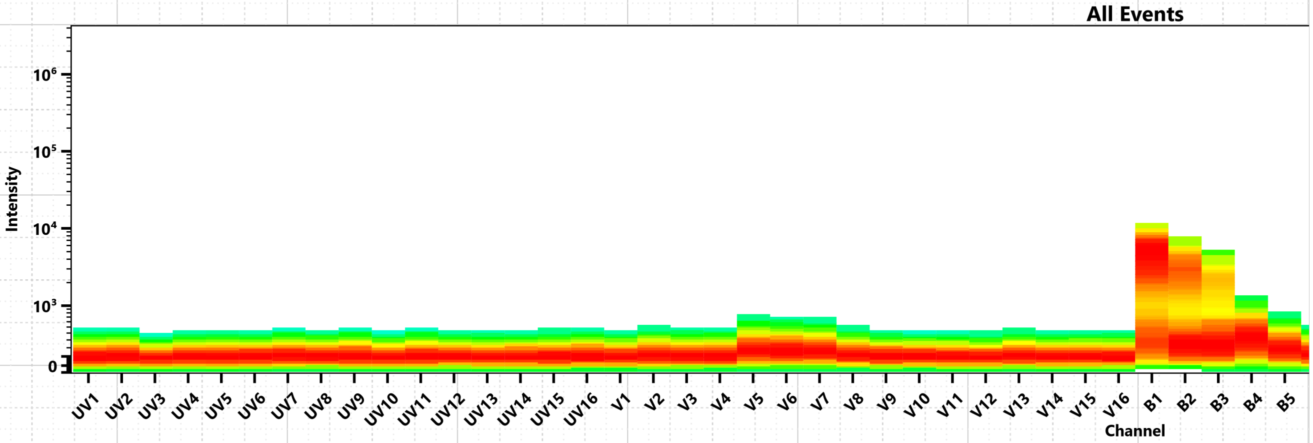

B5-A B6-A B7-A B8-A

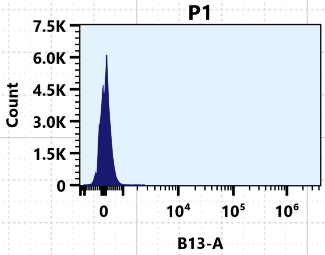

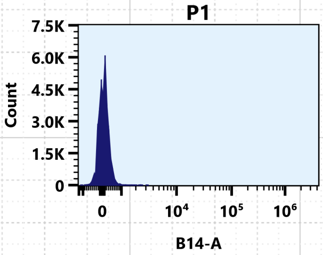

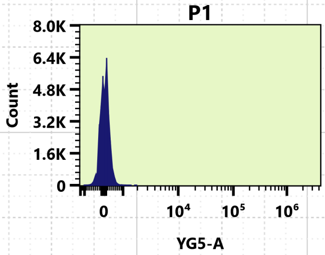

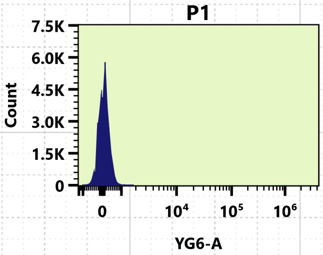

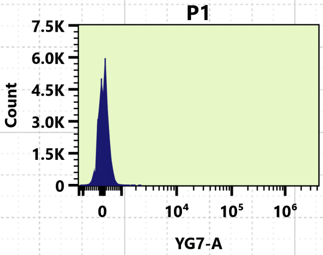

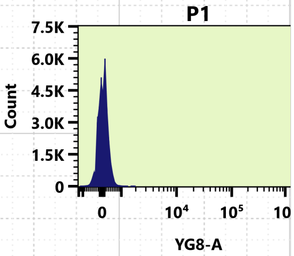

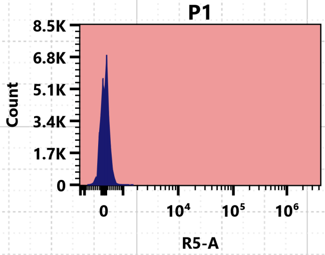

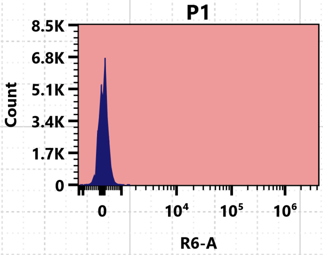

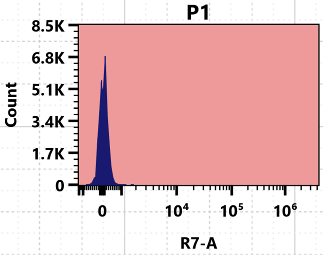

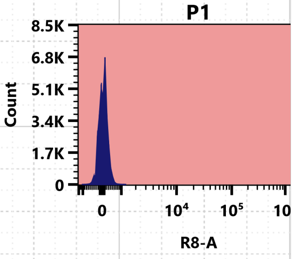

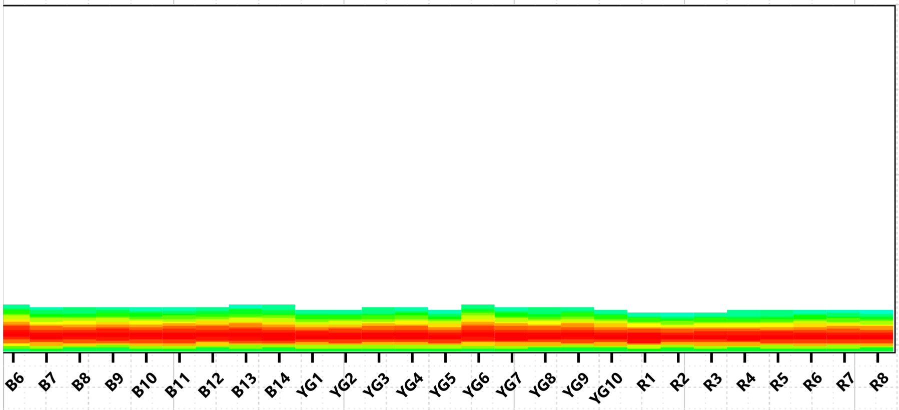

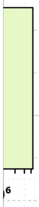

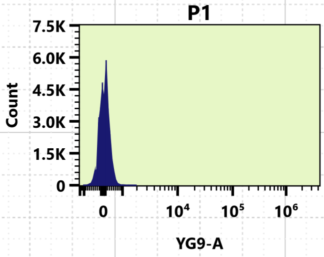
P1
Count
7.5K
6.0K
4.5K
3.0K
1.5K
0
0
10⁴
10⁵
10⁶
YG9-A

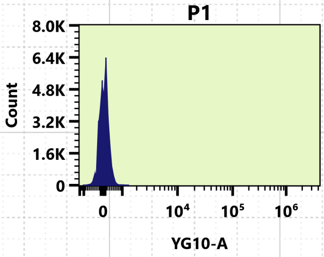
P1
Count
8.0K
6.4K
4.8K
3.2K
1.6K
0
0
10⁴
10⁵
10⁶
YG10-A

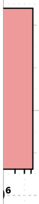

Supplement: Supplementary file 10 [file 44318_2026_899_MOESM16_ESM.zip › Figures EV1-4/Figure EV1/Fig. EV1B-C/data_files.zip!/vglut-GFP.pdf]
